# Female Aging Alters Expression of Human Cumulus Cells Genes that Are Essential for Oocyte Quality

**DOI:** 10.1155/2014/964614

**Published:** 2014-09-03

**Authors:** Tamadir Al-Edani, Said Assou, Alice Ferrières, Sophie Bringer Deutsch, Anna Gala, Charles-Henri Lecellier, Ounissa Aït-Ahmed, Samir Hamamah

**Affiliations:** ^1^UFR de Médecine, Université Montpellier 1, 34295 Montpellier, France; ^2^CHU Montpellier, Institut pour la Médecine Régénérative et Biothérapies, Hôpital Saint-Eloi, INSERM U1040, 34295 Montpellier, France; ^3^ART-PGD Department, CHU Montpellier, Hôpital Arnaud de Villeneuve, 34295 Montpellier, France; ^4^Institute of Molecular Genetics of Montpellier, 34293 Montpellier, France

## Abstract

Impact of female aging is an important issue in human reproduction. There was a need for an extensive analysis of age impact on transcriptome profile of cumulus cells (CCs) to link oocyte quality and developmental potential with patient's age. CCs from patients of three age groups were analyzed individually using microarrays. RT-qPCR validation was performed on independent CC cohorts. We focused here on pathways affected by aging in CCs that may explain the decline of oocyte quality with age. In CCs collected from patients >37 years, angiogenic genes including *ANGPTL4*, *LEPR*, *TGFBR3*, and *FGF2* were significantly overexpressed compared to patients of the two younger groups. In contrast genes implicated in TGF-*β* signaling pathway such as *AMH*, *TGFB1*, inhibin, and activin receptor were underexpressed. CCs from patients whose ages are between 31 and 36 years showed an overexpression of genes related to insulin signaling pathway such as *IGFBP3*, *PIK3R1*, and *IGFBP5*. A bioinformatic analysis was performed to identify the microRNAs that are potential regulators of the differentially expressed genes of the study. It revealed that the pathways impacted by age were potential targets of specific miRNAs previously identified in our CCs small RNAs sequencing.

## 1. Introduction

In developing countries, the first baby is conceived with a delay that keeps increasing. With aging there is natural decline in female fertility, which raises crucial issues for the society. The fertility decline is slow and steady in 30 to 35 years old women. However, this decline accelerates past 35 years due to the decrease in oocyte quality and ovarian reserve [1, 2]. Therefore female age is crucial and oocyte aging is a common cause of assisted reproduction technology failures [3]. MII oocyte stores large quantities of mRNA and proteins and contains a high number of mitochondria [4, 5]. Oocytes from women with an advanced reproductive age may have an increase of oxidative stress with consequences on mitochondrial DNA (mtDNA) integrity, resulting in mitochondrial dysfunction [6, 7]. Interestingly transcriptome profiles showed a substantial difference between younger and older human oocytes [8]. Moreover the increase of aneuploidy due to aging is well documented. Indeed, the link between female age and oocyte aneuploidy prevalence was extensively studied [9]. However both intrinsic (oocyte) and/or extrinsic (follicular) factors may be involved in the oocyte quality decline. The ovarian follicular microenvironment, mediated through cumulus cells (CCs), is crucial for the development of competent oocytes [10]. The CCs are in physical contact with the oocyte; together they form the cumulus-oocyte complex (COC) and undergo a cross-talk [11]. The oocyte controls the differentiation and expansion of CCs, which in turn are responsible for the metabolism of the glucose and pyruvate used for energy production in the oocyte [12]. An aged follicular microenvironment could impact oocytes and leave a characteristic transcriptional footprint in the surrounding CCs. Indeed, the use of human CC gene expression has proved powerful as a noninvasive approach to predict oocyte quality and developmental potential [13–16]. The analysis of gene expression in human CCs in relation to female age is based on the same rationale [17–19]. However, with the exception of one proteomic analysis [17], no high throughput study based on gene expression profile in relation to female age was performed on cumulus cells. Our hypothesis here is based on the assumption that female age may have a wide impact on gene expression and may specifically affect pathways that are critical for oocyte quality and development. The purposes of this study were (i) to thoroughly evaluate impact of maternal age on gene expression profiles using individual CCs isolated from the periovulatory follicles of three age categories of patients, (ii) to characterize the pathways that were significantly affected by female aging, and (iii) to identify their miRNAs regulators.

## 2. Materials and Methods

### 2.1. Sample Characterization and Collection

The Review Board of the Institute of Research in Biotherapy approved this project. All patients provided their written informed consent for the use of CC samples for research.

CC samples were collected from patients who participated to the multicentric trial previously described [20] and from Montpellier ART centre. Patients were stimulated with a combination of GnRH antagonist protocol with recombinant FSH or with HP-hMG before undergoing intracytoplasmic sperm injection (ICSI) procedure for male infertility. Cumulus oocyte complexes (COCs) were recovered under ultrasound echo-guidance 36 h after human Chorionic Gonadotrophin (5,000 UI, hCG) administration. CCs were separated mechanically from the corresponding oocyte as previously described [14]. For microarray 28 individual CC samples obtained from 16 patients were classified into three age groups: <30 years (CC_younger_), 31–34 years (CC_median_), and >37 years (CC_older_). The qRT-PCR analyses were performed on 15 independent CCs from the above groups and 4 CCs from a 35-36 additional group.

### 2.2. RNA Extraction and Microarray Processing

CCs were frozen at −80°C in RLT buffer before RNA extraction. Then the RNeasy Micro kit (ref: 74004; Qiagen) was used to extract total RNA from each CC sample, according to the manufacturers' recommended protocols. The quantity and purity of the total RNAs were determined by using a NanoDrop ND-1000 spectrophotometer (NanoDrop ND-Thermo Fisher Scientific, Wilmington, DE, USA) and their integrity determined by using the Agilent 2100 Bioanalyzer (Agilent Technologies, Palo Alto, CA, http://www.agilent.com). All RNA samples were stored at −80°C. Microarray experiments were performed on the microarray platform of Institute of Research in Biotherapy at the Montpellier University Hospital. The Affymetrix 3′ IVT express protocol (reference 901229) was used as previously described [20].

### 2.3. Microarray Data Analysis

After image processing with the Affymetrix GeneChip Operating 1.4 software, the CEL files were analyzed using the Affymetrix Expression Console Software v1.3.1 and normalized with the MAS5.0 algorithm by scaling each array to a target value of 100 using the global scaling method. This algorithm also determines whether a gene is expressed with a defined “detection call.” This “call” can either be “present” (when the perfect match probes are significantly more hybridized than the mismatch probes, *P* < 0.04), “marginal” (0.04 < *P* < 0.06), or “absent” (*P* > 0.06). Gene annotation was performed using NetAffx (http://www.affymetrix.com; March 2009). A first selection using the detection call (present in at least seven samples) and variation coefficient (≥40%) of CC samples identified 9,802 transcripts. Then, to compare the three groups of CCs according to maternal age, a Significance Analysis of Microarrays-Multiclass (SAM-M) (http://statweb.stanford.edu/~tibs/SAM/) was used. SAM-M handed the significantly expressed genes with a* q*-value <5% in the three age categories. CLUSTER and TREEVIEW software packages were used for the hierarchical clustering analysis. SPSS 12.0 (SPSS, Chicago, IL) software was used for box-and-whisker plots representation of expression levels of specific genes. The miRNA target predictions were performed with GeneGo MetaCore analysis software (St. Joseph, MI). Ingenuity Pathway Analysis software and DAVID (http://david.abcc.ncifcrf.gov/) were used for functional annotation.

### 2.4. Quantitative RT-PCR

Reverse transcription (RT) was performed as recommended by the manufacturer (Invitrogen) with 150 ng of RNA in a 20 *μ*L reaction volume that included Superscript II (ref. 18064-014, Invitrogen), oligo-dT primer, dNTP mixture, MgCl2, and RNase inhibitor. Quantitative PCR was performed using the SYBR Green I Master kit (Roche Diagnostics, Mannheim, Germany) with 2 *μ*L of 1/20 dilution of the RT reaction product and 0.5 mM primer (SIGMA Genosys) in a total volume of 10 *μ*L. The amplification was run in a LightCycler 480 apparatus as follows: after the denaturation step for 10 min at 95°C, cycling conditions were 10 s at 95°C, 30 s at 65°C and 1 s at 72°C for 45 cycles. Gene expression levels were normalized to the housekeeping gene Glyceraldehyde 3-Phosphate Dehydrogenase (*GAPDH*) using the following formula 100/2^ΔΔCt^ where ΔΔCt = ΔCt_unknown_ − ΔCt_positive control_. The primer sequences are shown in (see Table SI in Supplementary Material available online at http://dx.doi.org/10.1155/2014/964614).

### 2.5. Statistical Analysis

Statistical analysis was performed with the GraphPad InStat 3 software. For qRT-PCR, the Kruskal-Wallis nonparametric test was used. The differences among the groups were considered significant when the* P *value is <0.05.

## 3. Results

### 3.1. Gene Expression Profiles of CCs according to Female Age

**Figure 1:**
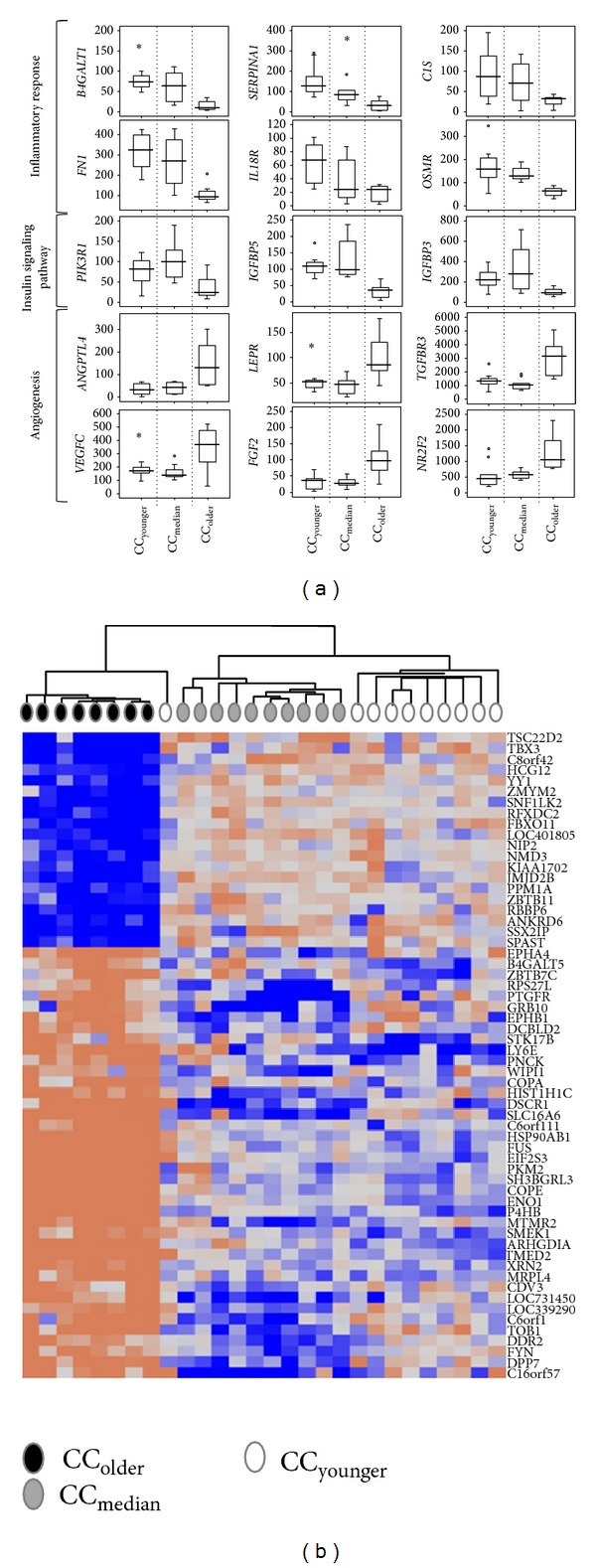
(a) Expression of cumulus cells genes according to female age. Box-and-whisker plots that represent expression of genes implicated in different biological processes and signaling pathways in the three female age categories, CC_younger_, CC_median_, and CC_older_. The signal intensity of each gene is shown on the *y* axis as arbitrary units determined by the Affymetrix GCOS software. (b) Heat map and cluster dendograms of differentially expressed genes. Hierarchical clustering is shown for 20 genes with the highest expression level in each of the 3 age categories of individual CCs. Overexpressed and underexpressed genes were marked in blue and pink, respectively. The three age categories are shown in white for CC_younger_, grey for CC_median_, and black for CC_older_.

In order to gain insight into the molecular basis of age impact on COCs, we analyzed the transcriptomes of CCs from women with different age categories. A first selection based on the detection call and variation coefficient of all the CC samples from aged and young patients delineated 9,802 transcripts. Then, using SAM-M and after having discarded 35 genes that we previously showed to be affected by the COS protocols [20], we identified a total of 2,186 transcripts (corresponding to 1,874 genes) with a* q*-value <5% that significantly distinguished the three CC groups according to female age (Supplementary Table SII). The analysis of the transcriptome data revealed a characteristic molecular signature for each one of the three age categories (Figure 1). The expression patterns of the genes that best represent these categories are illustrated in the box-plots (Figure 1(a)). In CC_younger_ group, overexpression was observed for inflammatory response genes such as* B4GALT1, SERPINA1, C1S, IL18R1, FN1,* and* OSMR*. The CC_median_ group revealed overexpression of genes involved in insulin signaling pathway, the most representative being* IGFBP3, IGFBP5* and* PIK3R1*. Finally the CC_older_ group was significantly enriched with genes that are important for angiogenesis such as* ANGPTL4, LEPR, TGFBR3, VEGFC, FGF2* and* NR2F2*. In addition, a list of 20 genes with the highest contrast and lowest* q*-value according to SAM-M, were chosen for each category to perform the hierarchical clustering (Supplementary Table SIII). Interestingly, CC_older_ samples distantly located from the CC_younger_ and CC_median_ samples (Figure 1(b)).

### 3.2. Validation of Gene Expression by Quantitative RT-PCR

**Figure 2:**
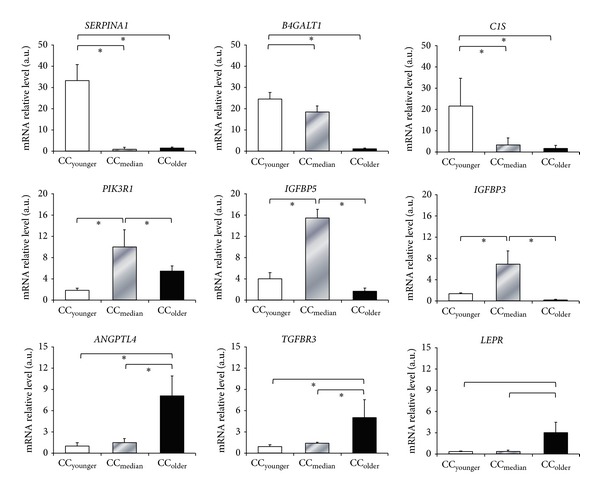
Validation by qRT-PCR of some gene members of key pathways that are differentially expressed in the three age categories. This figure shows the mRNA relative abundance of three genes implicated in inflammatory response (*SERPINA1*,* B4GALT1*, and* C1S*), three genes in insulin signaling (*PIK3R*,* IGFBP3*, and* IGFBP5*), and three genes in angiogenesis process (*ANGPTL4*,* TGFBR3*, and* LEPR*). The signal intensity for each gene is shown on the *y*-axis in arbitrary units determined by RT-qPCR analysis. *  indicates a significant difference of gene expression between CCs categories (*P < 0.05). Results were presented as the mean ± SEM. CC_younger_ (white, age: <30 years), CC_median_ (grey, age: 31–34 years), and CC_older_ (Black, age >37 years).

Nine differentially expressed genes were selected for validation on the basis of relevant functional annotations. Hence, three genes involved in the inflammatory process (*B4GALT1*,* SERPINA1,* and* C1S*), three genes of the insulin signaling (*IGFBP3*,* IGFBP5,* and* PIK3R1*) and three genes of the angiogenesis process (*ANGPTL4*,* LEPR, *and* TGFBR3*) were chosen for qRT-PCR validation. Analysis of the qRT-PCR data on independent cohorts of CCs indicated that all the selected genes were differentially expressed in the three age categories and in agreement with the microarray findings (Figure 2). Using qRT-PCR we aimed to test the expression level of the above genes in individual CCs from 35 and 36 old patients. These CCs clearly displayed an expression pattern similar to the CC_median_ age category (Figure SI) suggesting that the switch for these genes occurs after the age of 36.

### 3.3. Deregulation in CC_older_ Genes that Are Essential for the Oocyte Quality and Competence

**Figure 3:**
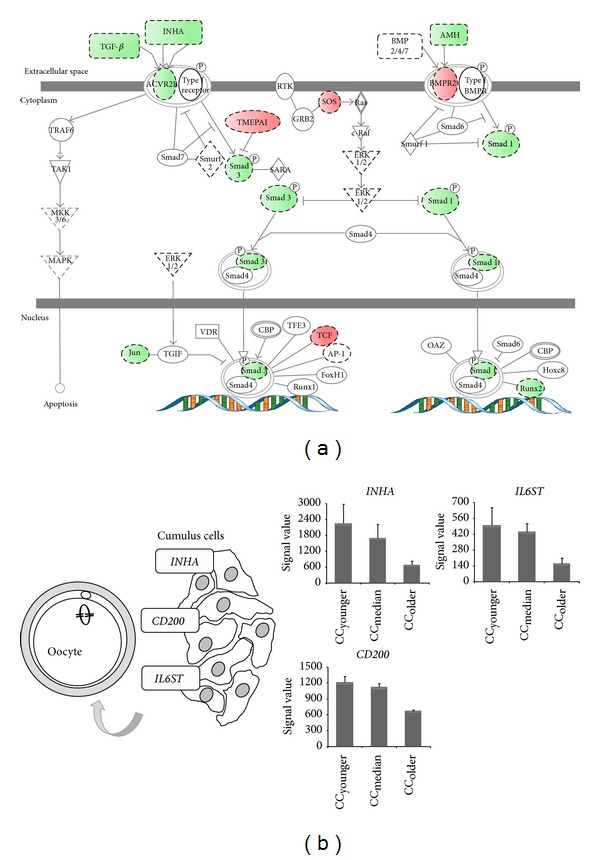
(a) TGF-*β* signaling pathway was deregulated in older CCs. The Ingenuity Pathway Analysis software was used to analyze impact of maternal age on TGF-*β* signaling. Downregulated genes in older CCs are shown in green and upregulated ones in red. Uncolored genes were not differentially expressed by our analysis but were integrated into the computationally generated networks on the basis of the evidence stored in the IPA knowledge memory indicating a relevance to this network. A plain line indicates direct interactions, a dashed line indicates indirect interactions, a line without arrowhead indicates binding only, a line finishing with a vertical line indicates inhibition, and a line with an arrowhead indicates “acts on.” (b) Schematic representation of genes upregulated in CC_younger_ and CC_median_ and downregulated in CC_older_ that are involved in cumulus-oocyte complex and oocyte development. Histograms show signal values of genes (*INHA, CD200, and IL6ST*) that are differentially expressed between age categories. Gene expression is measured by pan-genomic HG-U133 Plus 2.0 Affymetrix oligonucleotides microarrays, and the signal intensity for each gene is shown on the *y*-axis as arbitrary units determined by the GCOS 1.2 software (Affymetrix).

Many biological pathways were reported to be crucial for their impact on the oocyte development. They include transforming growth factor *β* (TGF-*β*) signaling, steroidogenesis and metabolic pathways. Interestingly the key members of these pathways displayed significant changes in their gene expression (Table SII). As shown in Figure 3(a), many genes of the TGF-*β* signaling pathway were underexpressed in CC_older_ compared with CC_younger_ and CC_median_, including* AMH* (Anti-Mullerien Hormone),* TGFB1*, inhibin (*INHA*) and activin receptor (*ACVR2B*). In contrast overexpression was observed in CC_older_ for several genes that are involved in steroidogenesis and fatty acid metabolism (*HSD17B1, HSD17B6, NSDHL, SRA1, CYP19A1, PPARA*), glucose metabolism (*ALG13, GLT8D3*) and glucose transporters (*SLC2A3*,* SLC2A1*,* SLC2A13*,* SLC2A8*). It is noteworthy that several genes that play an essential role in the cumulus-oocyte dialog (*INHA*,* CD200* and* IL6ST*) were downregulated in CC_older_ (Figure 3(b)). Moreover, CC_older_ may be distinguished from the two younger age categories by a downregulation of genes that are essential for genome integrity, in particular* MSRB3, UCHL5IP, POLH, OBFC2B,* and* CHAF1A* that are essential for antioxidative and DNA repair functions.

### 3.4. Potential miRNA Regulators of the Differentially Expressed Genes of the Study

**Figure 4:**
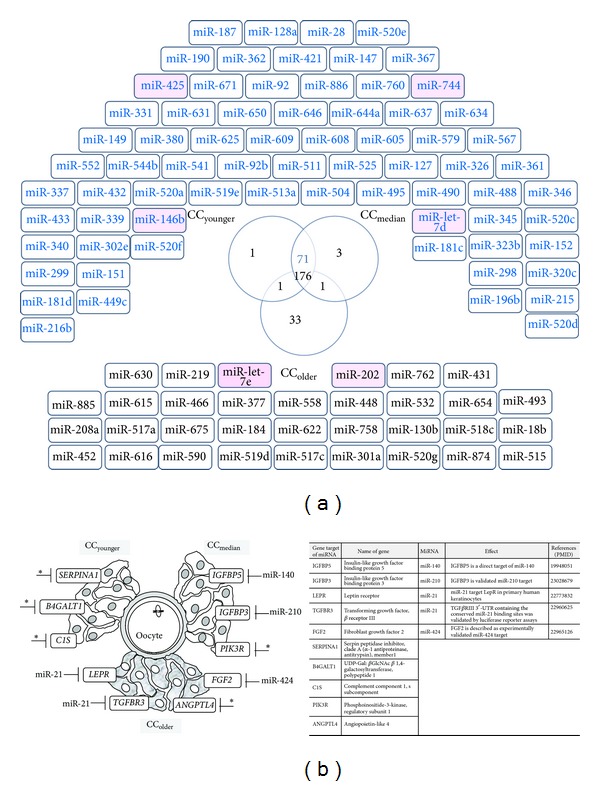
(a) Venn diagram representing the number of miRNAs retrieved from the GenGo analysis. The genes overexpressed in each age category were submitted to GenGo to identify their potential miRNA regulators. 249 miRNAs were retrieved for the CC_younger_ group, 251 for the CC_median_, and 211 for the CC_older_. The Venn diagram drawn after these lists shows that the majority is common to the three age categories. The miRNAs that are detected in the cumulus cells by using deep-sequencing approach [21] are shown in pink. (b) Schematic representation of some of the validated genes of the three pathways and processes discussed in this work and their miRNA regulators. Only the miRNAs that were found in the CCs small RNA sequencing and reported in the literature to be experimentally validated were represented. *  indicates the validated genes with no miRNA regulator that meets these criteria.

Using the GenGo Metacore software, we first aimed to identify which miRNAs regulate the genes that were overexpressed in each of the three age categories, CC_younger_, CC_median_ and CC_older_ (Figure 4(a)). We identified altogether 286 miRNAs that are putative regulators of the differentially expressed genes identified in this study, among which 176 are common putative regulators of the genes overexpressed in the three age categories, 71 for the genes whose expression was higher in CC_younger_ and CC_median_. Only one miRNA was shared by CC_median_ and CC_older_ categories specifically; similarly genes overexpressed in CC_younger_ and CC_older_ had one specific miRNA in common. Interestingly this analysis also discriminates the CC_older_ from CC_younger_ and CC_median_, which may be considered as a super-group with common features. Some miRNAs were specific for one of the three age categories. Thirty-three miRNAs were identified as putative regulators of the genes overexpressed in CC_older_, one for the CC_younger_ and 3 for the CC_median_ categories (for the comprehensive lists, see Supplementary Table SIV). Among all the miRNAs retrieved by GenGo, 87% were identified by sequencing in CCs [21]. The fact that only the differentially expressed genes were submitted to GenGO may account for the missing 13%. There is another discrepancy between the list of the potential regulators and the miRNAs actually present in the CCs as identified in our previous work [21]. It is illustrated in Figure 4(a) for the two categories that stand out in the present study, namely the CC_youger_-CC_median_ super-group (71) on the one hand and the CC_older_ (33) on the other hand. Among these potential miRNA regulators, only 6 are actually expressed in CCs:* MIR425*,* MIR744*,* MIR146b*,* Let-7d* for the CC_youger_-CC_median_ super group and* MIR202, Let-7e* for the CC_older_. This discrepancy might reflect a tissue specific expression of miRNAs. Interestingly* MIR202* is a potential regulator of the hyaluronan synthase-encoding gene* HAS2 *that is related to aging and angiogenesis [22] and* MIR744* is a* TGFB1* validated regulator [23]. The largest set of miRNAs retrieved by GenGo was common to the three age categories (176). This set was crossed with those effectively expressed in CCs [21], resulting in a list of 22 miRNAs. We were interested in those that regulate significant gene members of the pathways and processes impacted by female age and that were also experimentally validated. The results of this analysis are shown in Figure 4(b). None fulfills these criteria for the validated genes of the inflammatory process overexpressed in the CC_younger_. In CC_median_,* IGFBP3, and IGFBP5* of the insulin-signaling pathway are targets of* MIR210* and* MIR140*, respectively. Finally in CC_older_, genes implicated in angiogenesis* LEPR *and TGFBR3 are* MIR21* targets whereas* FGF2* is targeted by* MIR424*. For more details see Table SV.

## 4. Discussion

Acquisition of oocyte competence is a gradual and complex process, which depends on the follicular microenvironment. Within this microenvironment, the bidirectional communication between the CCs and the oocyte plays a crucial role. Therefore gene expression in CCs mirrors the oocyte physiology. In order to gain insight into the mechanisms that underlie oocyte quality decline with age, we first investigated the transcriptome profiles in CCs from women of three age categories. Our objective was to identify molecular signatures characteristic of each age category and investigate their biological relevance to oocyte quality. DNA microarray analysis revealed a significantly distinct molecular signature of 1,874 genes among the three age groups, suggesting a wide impact of female age on the CC gene-expression profile. It is noteworthy that the inflammatory genes emerged in the CC_younger_ group such as* IL18R1*,* IL1R1, IL1R2, SERPINA1,* and* B4GALT1.* Inflammatory reaction is known to induce ovulation through infiltration of leukocytes into the area surrounding the follicle [24]. Cytokines are important in the regulation of ovarian function and oocyte quality [25]. On the other hand interleukins* IL18* and* IL1* *β* were reported to be present in floating granulosa cells of human preovulatory follicles [26]. CC_median_ group may be characterized by an overexpression of gene members of the “insulin-signaling pathway”, such as* IGFBP3 *and* IGFBP5* whereas* INSR *was overexpressed in both the CC_median_ and CC_older_ groups. Several studies have shown that insulin and* IGF* system play an important role in folliculogenesis [27–29] and in oocyte maturation [30]. IGF-binding proteins (*IGFBPs*) that modulate interactions of* IGFs* with* IGF* and insulin receptors [31] have also an antiangiogenic activity [32–34]. Therefore, overexpression of* IGFBPs* in CC_median_ may be to modulate angiogenesis and maintain a balance. Last, the CC_older_ group is precisely characterized by an upregulation of genes associated with angiogenesis (*ANGPTL4, LEPR, TGFBR3, VEGFC, FGF2* and* NR2F2*). Angiogenesis plays a critical role in the late stages of folliculogenesis by providing nutrients and oxygen to the growing follicles. However, it may be associated with pathology and induced by microenvironmental factors like hypoxia. In this context, the follicular cells synthesize several angiogenic factors [26, 35, 36], among which the vascular endothelial growth factor C (*VEGFC*) and angiopoietin-like 4 (*ANGPTL4*), which are induced in response to hypoxic stimuli [37–39]. So, the overexpression of angiogenic factors and hypoxia-inducible protein 2 (*HIG2*) in the CC_older_ group could be caused by insufficiency of oxygen. Similarly* VEGF* that is shown to increase in follicular fluid with age could be enhanced by hypoxia in old follicles [40, 41]. Most interestingly oocytes from hypoxic follicles have disorganized meiotic spindles [42]. These observations added to the reported increase of aneuploidy with female aging [43] may be revisited in light of our results. Hypoxia might be one of the consequences of aging, which in turn would affect chromosome segregation. Adaptive changes to oxygen availability are critical for cell survival and tissue homeostasis. Therefore, augmentation of angiogenesis in the CC_older_ group may be a compensatory process to modulate the deleterious impact of hypoxia. Similarly the upregulation of genes that encode metabolic enzymes (*HSD17B*,* CYP19A1,* ALG13, and GLT8D3) and glucose transporters (*SLC2A3*,* SLC2A1*,* SLC2A13*,* SLC2A8*) in the CC_older_ group could reflect a compensatory mechanism to increase energy production. These results are consistent with the observations reported recently [17, 19]. Indeed, the energy supplied by the CCs is known to be required for oocyte quality [44, 45]. Some members of the *TGF*-*β* superfamily, which are crucial to processes that govern follicle development and oocyte maturation [46], were underexpressed in the CC_older_ group such as* AMH* (Anti-Mullerien Hormone), *TGFB1*, inhibin (*INHA*),and activin receptor (*ACVR2B*). Interestingly* AMH* is produced by early primary follicles and its mRNA level is known to decrease with age. Therefore, it represents an early marker of ovarian follicle growth and a reliable marker of ovarian reserve and oocyte quality [47–49].

Another important question we addressed concerns the regulation of the genes that stand out in our study. We focused on the bioinformatic analysis of miRNAs. MiRNAs are noncoding small RNAs (18–25 nucleotides), which regulate cellular genes through RNA degradation or translational inhibition [50, 51]. Not only miRNAs have been shown to regulate the aging process in different tissues and cells [52], but their importance is also well recognized in the control of human cumulus-oocyte crosstalk and ovarian function and aging [21, 53, 54]. Interestingly, *TGF*-*β* signaling is one of the most significant pathways targeted by miRNAs contained in the follicular fluid [55]. Moreover gene members of this pathway are direct targets of* MIR21* that is the most abundant miRNA in CCs [21]. The role of *MIR21* is essential in ovarian function toprevent apoptosis in mouse periovulatory granulosa cells both *in vivo* and *in vitro* [56]. Moreover, it promotes the follicular cell survival during ovulation and is upregulated during luteinization [57]. Interestingly, a recent work reports a correlation between *MIR21* abundance and women age; a significant decrease was observed in follicular fluid of older women [53]. In the current study, two angiogenic genes (*LEPR* and* TGFBR3*) were upregulated in CC_older_ where* MIR21* is the least abundant [53]. Finally, the process that is central to this study is angiogenesis that may be induced in response to hypoxia, a major issue in aging follicles. Interestingly, miRNAs play a critical role in the cellular response to hypoxia [58].* MIR210* whose overexpressionin hypoxic conditions induces angiogenesis [59, 60] directly targets *IGFBP3*, an inhibitor of angiogenesis [32, 61]. Furthermore,* MIR424 *that is downregulated in response to hypoxia in primary human trophoblasts [62] targets *FGF2*, an angiogenesis inducer [63, 64]. Taken together these data suggest that in aging follicles angiogenesis may be induced in response to hypoxia by the underexpression of* IGFBP3 *and overexpression of* FGF2*.

## 5. Conclusion

The present study reports for the first time an extensive analysis of gene expression in cumulus cells in relation to female age. Specific molecular signatures were characterized for the three age categories. Our findings point to aging as a major player in processes and pathways that are of key biological importance for oocyte growth and genome integrity. Moreover the upregulation of angiogenic genes in CC_older_ is very informative on the way the follicle attempts to buffer the deleterious impact of aging associated hypoxia. In addition to the transcriptomes, the comprehensive characterization of the miRNA regulators of the genes impacted by female age represents a valuable resource for future investigations on the biology of aging oocyte.

## Supplementary Material

Supplementary Material available online includes: (i) Sequence of the forward and reverse primers used for the qRT-PCR analysis. (ii) List of the 1,874 genes whose differential expression in the three age groups was significant (iii) List of the 60 genes (20 for each age category) used for the hierarchical clustering. (iv) exhaustive lists of miRNAs that are putative regulators of genes over-expressed in CC_younger_, CC_median_, and CC_older_, retrieved by GenGo. (v) GenGo predicted miRNAs that target some significant genes implicated in inflammatory response, angiogenesis, insulin and *TGF*-*β* signaling pathways. (vi) qRT-PCR validation on independent cohorts of individual CCs of gene members of the key pathways that are discussed in the manuscript with the addition of a 4th age category (age: 35-36 years).
