# Supplementary material for: Female Aging Alters Expression of Human Cumulus Cells Genes that Are Essential for Oocyte Quality
Source: Biomed Res Int. 2014 Sep 3;2014:964614. doi: 10.1155/2014/964614 (PMC4168028; doi:10.1155/2014/964614)
Supplement: Supplementary file 1 — Supplementary Material available online includes: (i) Sequence of the forward and reverse primers used for the qRT-PCR analysis. (ii) List of the 1,874 genes whose differential expression in the three age groups was significant (iii) List of the 60 genes (20 for each age category) used for the hierarchical clustering. (iv) exhaustive lists of miRNAs that are putative regulators of genes over-expressed in CCyounger, CCmedian, and CColder, retrieved by GenGo. (v) GenGo predicted miRNAs that target some significant genes implicated in inflammatory response, angiogenesis, insulin and TGF-β signaling pathways. (vi) qRT-PCR validation on independent cohorts of individual CCs of gene members of the key pathways that are discussed in the manuscript with the addition of a 4th age category (age: 35-36 years). [file 964614.f1.doc]

Supplementary data


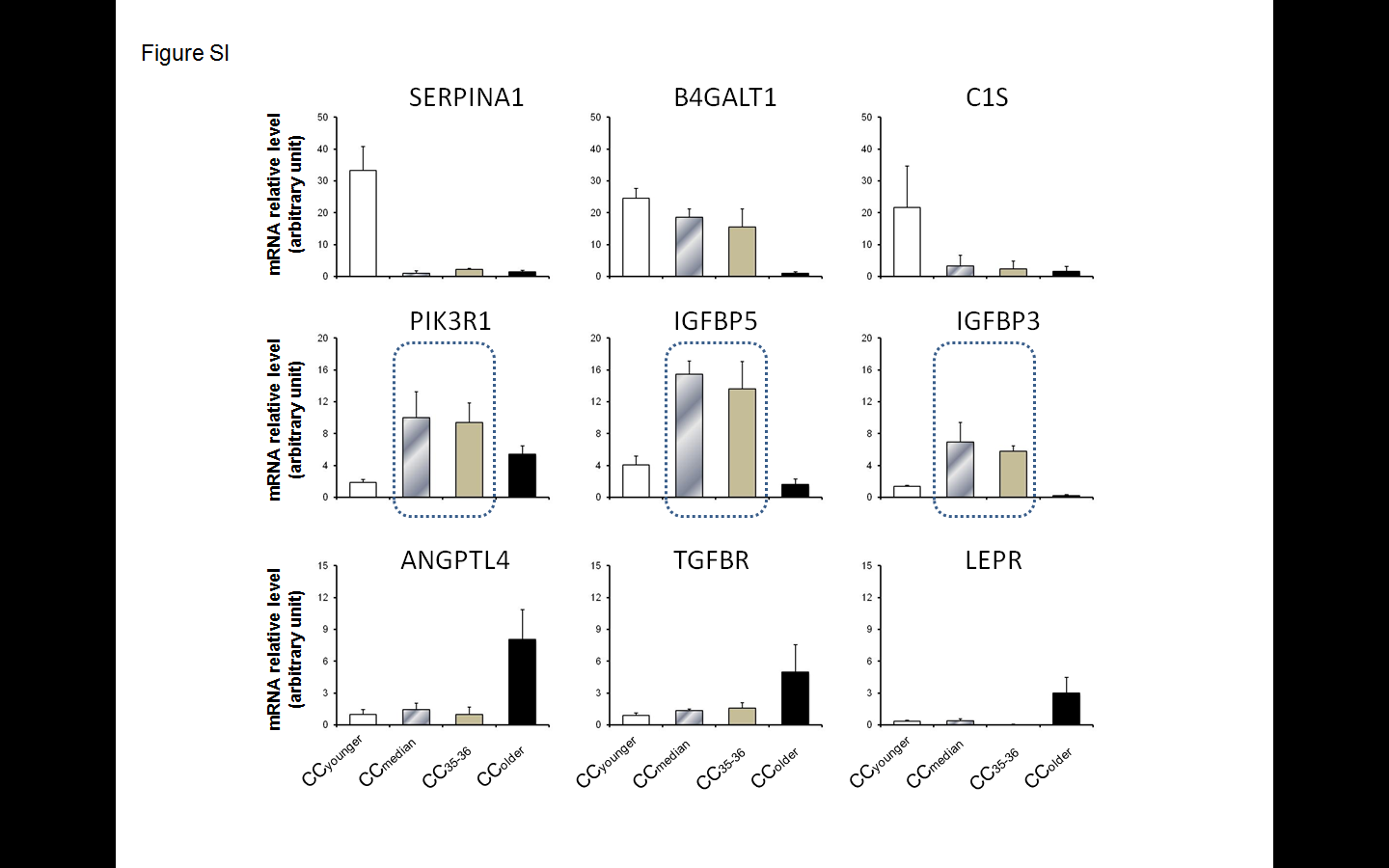


**Table SI:** Sequence of the forward and reverse primers used for qRT-PCR analysis

| **Gene symbol** | **Gene name** | **Primer 5'→3'** |
| --- | --- | --- |
| Forward (F)  Reverse (R) | | |
| C1S | Complement component 1, s subcomponent | F: GTCATCCGCTACACTTGTGAGG |
|  |  | R: CTGGAACACATTTCGGCAGCTC |
| B4GALT1 | UDP-Gal:betaGlcNAc beta 1,4- galactosyltransferase, polypeptide 1 | F: GTATTTTGGAGGTGTCTCTGCTC |
|  |  | R: GGGCGAGATATAGACATGCCTC |
| SERPINA1 | Serpin peptidase inhibitor, clade A (alpha-1 antiproteinase, antitrypsin), member 1 | F: TCTGAAGAGCGTCCTGGGTCAA |
|  |  | R: GATGGTCAGCACAGCCTTATGC |
| IGFBP3 | Insulin-like growth factor binding protein 3 | F: CGCTACAAAGTTGACTACGAGTC |
|  |  | R: GTCTTCCATTTCTCTACGGCAGG |
| PIK3R1 | Phosphoinositide-3-kinase, regulatory subunit 1 (p85 alpha) | F: CGCCTCTTCTTATCAAGCTCGTG |
|  |  | R: GAAGCTGTCGTAATTCTGCCAGG |
| TGFBR3 | Transforming growth factor, beta receptor III (betaglycan, 300kDa) | F: TGGAGTCTCCTCTGAATGGCTG |
|  |  | R: CCATTATCACCTGACTCCAGATC |
| ANGPTL4 | Angiopoietin-like 4 | F: GATGGCTCAGTGGACTTCAACC |
|  |  | R: TGCTATGCACCTTCTCCAGACC |
| LEPR | Leptin receptor | F: GCAGTCTATGCTGTTCAGGTGC |
|  |  | R: CCAAAATTCAGGTCCTCTCATAGG |
| IGFBP5 | Insulin-like growth factor binding protein 5 | F: CGTGCTGTGTACCTGCCCAATT |
|  |  | R: ACTTGTCCACGCACCAGCAGAT |

**Table SII**

| **Gene ID** | **Gene Name** | **Gene Title** | **CC younger (contrast-1)** | **CC median (contrast-2)** | **CC older (contrast-3)** | **q-value(%)** |  |
| --- | --- | --- | --- | --- | --- | --- | --- |
| 222630_at | RFXDC2 | regulatory factor X domain containing 2 | -2,74116 | -3,19246 | 7,41702 | 0 |  |
| 213221_s_at | SNF1LK2 | SNF1-like kinase 2 | -2,174584 | -3,42103 | 6,99451 | 0 |  |
| 203255_at | FBXO11 | F-box protein 11 | -2,335754 | -2,34728 | 5,85379 | 0 |  |
| 212781_at | RBBP6 | retinoblastoma binding protein 6 | -1,69647 | -2,55123 | 5,30962 | 0 |  |
| 231870_s_at | NMD3 | NMD3 homolog (S. cerevisiae) | -2,111731 | -2,04991 | 5,20206 | 0 |  |
| 209748_at | SPAST | spastin | -1,895352 | -2,23637 | 5,16465 | 0 |  |
| 227980_at | HCG12 | HLA complex group 12 | -2,269962 | -1,81057 | 5,10066 | 0 |  |
| 203016_s_at | SSX2IP | synovial sarcoma, X breakpoint 2 interacting protein | -1,854306 | -2,18775 | 5,05256 | 0 |  |
| 238949_at | LOC401805 | hypothetical gene supported by NM_144726 | -1,362221 | -2,65031 | 5,01567 | 0 |  |
| 212496_s_at | JMJD2B | jumonji domain containing 2B | -1,402438 | -2,59193 | 4,99296 | 0 |  |
| 238890_at | WDR9 | WD repeat domain 9 | -1,371108 | -2,62248 | 4,99198 | 0 |  |
| 204671_s_at | ANKRD6 | ankyrin repeat domain 6 | -1,980016 | -1,8365 | 4,77065 | 0 |  |
| 226280_at | NIP2 | CDNA FLJ43545 fis, clone PROST2011631 | -2,067484 | -1,7479 | 4,76924 | 0 |  |
| 224718_at | YY1 | YY1 transcription factor | -1,537327 | -2,21057 | 4,68487 | 0 |  |
| 230903_s_at | C8orf42 | Chromosome 8 open reading frame 42 | -1,478592 | -2,22623 | 4,63103 | 0 |  |
| 203966_s_at | PPM1A | protein phosphatase 1A (formerly 2C), magnesium-dependent, alpha isoform /// protein phosphatase 1A (formerly 2C), magnesium-dependent, alpha isoform | -1,539085 | -2,07646 | 4,51944 | 0 |  |
| 202778_s_at | ZMYM2 | zinc finger, MYM-type 2 | -1,662145 | -1,94703 | 4,51147 | 0 |  |
| 225544_at | TBX3 | T-box 3 (ulnar mammary syndrome) | -2,048939 | -1,54699 | 4,49492 | 0 |  |
| 204847_at | ZBTB11 | zinc finger and BTB domain containing 11 | -1,372618 | -2,15456 | 4,40897 | 0 |  |
| 1556060_a_at | KIAA1702 | KIAA1702 protein | -1,593312 | -1,92468 | 4,39749 | 0 |  |
| 240557_at | TSC22D2 | TSC22 domain family, member 2 | -1,356348 | -2,06269 | 4,2738 | 0 |  |
| 235045_at | RBM7 | RNA binding motif protein 7 | -1,109855 | -2,3041 | 4,26744 | 0 |  |
| 225889_at | AEBP2 | AE binding protein 2 | -1,432728 | -1,96148 | 4,24276 | 0 |  |
| 220933_s_at | ZCCHC6 | zinc finger, CCHC domain containing 6 | -1,51483 | -1,8405 | 4,19416 | 0 |  |
| 228115_at | FAM59A | family with sequence similarity 59, member A | -1,820414 | -1,52603 | 4,18305 | 0 |  |
| 226447_at | ASH1L | ash1 (absent, small, or homeotic)-like (Drosophila) | -1,218795 | -2,12427 | 4,17883 | 0 |  |
| 221766_s_at | FAM46A | family with sequence similarity 46, member A | -1,322133 | -1,99802 | 4,15019 | 0 |  |
| 227247_at | PLEKHA8 | Pleckstrin homology domain containing, family A (phosphoinositide binding specific) member 8 | -1,850099 | -1,39902 | 4,0614 | 0 |  |
| 225308_s_at | TANC1 | tetratricopeptide repeat, ankyrin repeat and coiled-coil containing 1 | -1,379994 | -1,86664 | 4,05829 | 0 |  |
| 238005_s_at | Sin3A | Transcribed locus | -1,046212 | -2,20037 | 4,05822 | 0 |  |
| 224162_s_at | FBXO31 | F-box protein 31 | -2,314928 | -0,90974 | 4,03083 | 0 |  |
| 242669_at | UFM1 | Ubiquitin-fold modifier 1 | -1,412213 | -1,8071 | 4,02414 | 0 |  |
| 214394_x_at | EEF1D /// LOC126037 | eukaryotic translation elongation factor 1 delta (guanine nucleotide exchange protein) /// similar to Elongation factor 1-delta (EF-1-delta) (Antigen NY-CO-4) | -1,598695 | -1,61998 | 4,02334 | 0 |  |
| 202140_s_at | CLK3 | CDC-like kinase 3 | -1,180635 | -1,99558 | 3,97027 | 0 |  |
| 230972_at | ANKRD9 | ankyrin repeat domain 9 | -1,393171 | -1,77799 | 3,96395 | 0 |  |
| 201597_at | COX7A2 | cytochrome c oxidase subunit VIIa polypeptide 2 (liver) | -1,276952 | -1,84966 | 3,90826 | 0 |  |
| 218134_s_at | RBM22 | RNA binding motif protein 22 | -1,590631 | -1,53294 | 3,90447 | 0 |  |
| 204225_at | HDAC4 | histone deacetylase 4 | -1,116559 | -2,00655 | 3,90389 | 0 |  |
| 201061_s_at | STOM | stomatin | -1,441673 | -1,67498 | 3,89581 | 0 |  |
| 1568623_a_at | SLC35E4 | solute carrier family 35, member E4 | -1,334811 | -1,77317 | 3,88497 | 0 |  |
| 228149_at | FLJ31818 | hypothetical protein FLJ31818 | -1,273788 | -1,82267 | 3,87057 | 0 |  |
| 212622_at | TMEM41B | transmembrane protein 41B | -1,24554 | -1,82955 | 3,84386 | 0 |  |
| 211270_x_at | PTBP1 | polypyrimidine tract binding protein 1 | -0,924466 | -2,1321 | 3,82071 | 0 |  |
| 202664_at | WIPF1 | WAS/WASL interacting protein family, member 1 | -1,486214 | -1,54005 | 3,78283 | 0 |  |
| 226045_at | FRS2 | fibroblast growth factor receptor substrate 2 | -1,320719 | -1,68199 | 3,75339 | 0 |  |
| 204068_at | STK3 | serine/threonine kinase 3 (STE20 homolog, yeast) | -0,615575 | -2,36022 | 3,71974 | 0 |  |
| 226297_at | HIPK3 | Homeodomain interacting protein kinase 3 | -1,645434 | -1,31685 | 3,70286 | 0 |  |
| 210721_s_at | PAK7 | p21(CDKN1A)-activated kinase 7 | -1,208155 | -1,74033 | 3,68561 | 0 |  |
| 230722_at | BNC2 | basonuclin 2 | -1,179767 | -1,76507 | 3,68105 | 0 |  |
| 221127_s_at | RIG | regulated in glioma | -1,190866 | -1,7457 | 3,67071 | 0 |  |
| 1554178_a_at | FAM126B | family with sequence similarity 126, member B | -1,302837 | -1,62693 | 3,6622 | 0 |  |
| 214696_at | MGC14376 | hypothetical protein MGC14376 | -1,300615 | -1,62613 | 3,65842 | 0 |  |
| 227020_at | YPEL2 | yippee-like 2 (Drosophila) | -1,621661 | -1,30241 | 3,65509 | 0 |  |
| 214104_at | GPR161 | G protein-coupled receptor 161 | -1,510911 | -1,40773 | 3,6483 | 0 |  |
| 209348_s_at | MAF | v-maf musculoaponeurotic fibrosarcoma oncogene homolog (avian) | -1,761234 | -1,15337 | 3,64326 | 0 |  |
| 230201_at | FXR1 | Transcribed locus | -1,176296 | -1,73271 | 3,63626 | 0 |  |
| 222853_at | FLRT3 | fibronectin leucine rich transmembrane protein 3 | -1,357283 | -1,54326 | 3,62568 | 0 |  |
| 236600_at | SPG20 | spastic paraplegia 20, spartin (Troyer syndrome) | -0,717463 | -2,17156 | 3,61128 | 0 |  |
| 209674_at | CRY1 | cryptochrome 1 (photolyase-like) | -0,992439 | -1,8883 | 3,60092 | 0 |  |
| 207513_s_at | ZNF189 | zinc finger protein 189 | -1,156781 | -1,7173 | 3,59261 | 0 |  |
| 1554452_a_at | HIG2 | hypoxia-inducible protein 2 | -1,713791 | -1,15119 | 3,58123 | 0 |  |
| 213891_s_at | ITF2 | CDNA FLJ11918 fis, clone HEMBB1000272 | -1,324366 | -1,52997 | 3,56792 | 0 |  |
| 212229_s_at | FBXO21 | F-box protein 21 | -1,027627 | -1,81847 | 3,55762 | 0 |  |
| 231175_at | C6orf65 | chromosome 6 open reading frame 65 | -1,285225 | -1,53228 | 3,52188 | 0 |  |
| 228754_at | SLC6A6 | solute carrier family 6 (neurotransmitter transporter, taurine), member 6 | -1,157574 | -1,65896 | 3,52067 | 0 |  |
| 226155_at | KIAA1600 | KIAA1600 | -1,250668 | -1,55359 | 3,50533 | 0 |  |
| 219112_at | RAPGEF6 | Rap guanine nucleotide exchange factor (GEF) 6 | -1,04501 | -1,75606 | 3,50134 | 0 |  |
| 242761_s_at | ZNF420 | zinc finger protein 420 | -0,827992 | -1,96974 | 3,49717 | 0 |  |
| 208935_s_at | LGALS8 | lectin, galactoside-binding, soluble, 8 (galectin 8) | -1,407236 | -1,3866 | 3,4923 | 0 |  |
| 229704_at | APRIN | Androgen-induced proliferation inhibitor | -1,607075 | -1,18426 | 3,48917 | 0 |  |
| 226460_at | KIAA1450 | KIAA1450 protein | -0,969071 | -1,80989 | 3,4737 | 0 |  |
| 31874_at | GAS2L1 | growth arrest-specific 2 like 1 | -2,156901 | -0,61929 | 3,47024 | 0 |  |
| 226119_at | PCMTD1 | protein-L-isoaspartate (D-aspartate) O-methyltransferase domain containing 1 | -1,717861 | -1,05718 | 3,4688 | 0 |  |
| 225445_at | FLJ25778 | Hypothetical protein FLJ25778 | -1,324405 | -1,44104 | 3,45681 | 0 |  |
| 225138_at | ZRANB1 | zinc finger, RAN-binding domain containing 1 | -1,579574 | -1,17905 | 3,44828 | 0 |  |
| 223182_s_at | AGPAT3 | 1-acylglycerol-3-phosphate O-acyltransferase 3 | -1,069316 | -1,68807 | 3,44674 | 0 |  |
| 225996_at | LONRF2 | LON peptidase N-terminal domain and ring finger 2 | -0,898892 | -1,84832 | 3,43402 | 0 |  |
| 218252_at | CKAP2 | cytoskeleton associated protein 2 | -0,893084 | -1,84649 | 3,42447 | 0 |  |
| 235338_s_at | SETDB2 | SET domain, bifurcated 2 | -0,934999 | -1,80081 | 3,41976 | 0 |  |
| 226844_at | MOBKL2B | MOB1, Mps One Binder kinase activator-like 2B (yeast) | -1,359857 | -1,37335 | 3,41651 | 0 |  |
| 226441_at | MAP3K2 (MEKK2) | CDNA FLJ36574 fis, clone TRACH2012376 | -0,885547 | -1,84401 | 3,41194 | 0 |  |
| 223437_at | PPARA | peroxisome proliferator-activated receptor alpha | -1,067688 | -1,66138 | 3,41134 | 0 |  |
| 225486_at | ARID2 | AT rich interactive domain 2 (ARID, RFX-like) | -1,692289 | -1,02979 | 3,40259 | 0 |  |
| 212774_at | ZNF238 | zinc finger protein 238 | -1,133729 | -1,58698 | 3,40089 | 0 |  |
| 228335_at | CLDN11 | claudin 11 (oligodendrocyte transmembrane protein) | -1,010603 | -1,70302 | 3,39203 | 0 |  |
| 220448_at | KCNK12 | potassium channel, subfamily K, member 12 | -1,511145 | -1,20118 | 3,39041 | 0 |  |
| 213164_at | SLC5A3 | solute carrier family 5 (inositol transporters), member 3 | -1,552419 | -1,10502 | 3,3218 | 0 |  |
| 201427_s_at | SEPP1 | selenoprotein P, plasma, 1 | -1,344985 | -1,31106 | 3,32005 | 0 |  |
| 226505_x_at | USP32 | ubiquitin specific peptidase 32 | -0,94525 | -1,71073 | 3,31997 | 0 |  |
| 206020_at | SOCS6 | suppressor of cytokine signaling 6 | -1,441254 | -1,19475 | 3,295 | 0 |  |
| 228662_at | SOCS7 | Transcribed locus, strongly similar to NP_001035149.1 phosphoprotein 1 isoform c [Homo sapiens] | -1,324659 | -1,30039 | 3,28131 | 0 |  |
| 226561_at | LOC285086 | hypothetical protein LOC285086 | -1,308519 | -1,31616 | 3,28085 | 0 |  |
| 216210_x_at | TRIOBP | TRIO and F-actin binding protein | -0,755886 | -1,86594 | 3,27729 | 0 |  |
| 214057_at | MCL1 | Myeloid cell leukemia sequence 1 (BCL2-related) | -1,564055 | -1,04768 | 3,26467 | 0 |  |
| 201250_s_at | SLC2A1 | solute carrier family 2 (facilitated glucose transporter), member 1 | -1,342064 | -1,26862 | 3,26336 | 0 |  |
| 228007_at | C6orf204 | Chromosome 6 open reading frame 204 | -1,765436 | -0,84041 | 3,2573 | 0 |  |
| 226317_at | PPP4R2 | protein phosphatase 4, regulatory subunit 2 | -1,000692 | -1,60449 | 3,25648 | 0 |  |
| 220244_at | LOH3CR2A | loss of heterozygosity, 3, chromosomal region 2, gene A | -1,103242 | -1,48235 | 3,23199 | 0 |  |
| 221506_s_at | TNPO2 | transportin 2 (importin 3, karyopherin beta 2b) | -1,177473 | -1,40599 | 3,22933 | 0 |  |
| 203097_s_at | RAPGEF2 | Rap guanine nucleotide exchange factor (GEF) 2 | -1,566551 | -1,01658 | 3,22891 | 0 |  |
| 203791_at | DMXL1 | Dmx-like 1 | -1,117347 | -1,46273 | 3,22509 | 0 |  |
| 203477_at | COL15A1 | collagen, type XV, alpha 1 | -1,129035 | -1,4499 | 3,22367 | 0 |  |
| 226006_at | LOC554363 | hypothetical gene LOC554363 | -1,374903 | -1,18475 | 3,19957 | 0 |  |
| 212804_s_at | GAPVD1 | GTPase activating protein and VPS9 domains 1 | -1,422201 | -1,13641 | 3,19826 | 0 |  |
| 203038_at | PTPRK | protein tyrosine phosphatase, receptor type, K | -1,295385 | -1,26056 | 3,19493 | 0 |  |
| 212723_at | PTDSR | phosphatidylserine receptor | -1,402731 | -1,15127 | 3,1925 | 0 |  |
| 203566_s_at | AGL | amylo-1, 6-glucosidase, 4-alpha-glucanotransferase (glycogen debranching enzyme, glycogen storage disease type III) | -1,222331 | -1,33097 | 3,19163 | 0 |  |
| 224864_at | SRA1 | steroid receptor RNA activator 1 | -1,533013 | -1,01125 | 3,18033 | 0 |  |
| 212989_at | TMEM23 | transmembrane protein 23 | -1,064618 | -1,47728 | 3,17738 | 0 |  |
| 227948_at | FGD4 | FYVE, RhoGEF and PH domain containing 4 | -1,141201 | -1,39369 | 3,16861 | 0 |  |
| 210346_s_at | CLK4 | CDC-like kinase 4 | -1,165758 | -1,3592 | 3,15619 | 0 |  |
| 229419_at | FBXW7 | F-box and WD-40 domain protein 7 (archipelago homolog, Drosophila) | -1,149229 | -1,37408 | 3,15414 | 0 |  |
| 212838_at | DNMBP | dynamin binding protein | -0,94672 | -1,56824 | 3,1437 | 0 |  |
| 202530_at | MAPK14 | mitogen-activated protein kinase 14 | -0,943872 | -1,5649 | 3,13596 | 0 |  |
| 202815_s_at | HEXIM1 | hexamethylene bis-acetamide inducible 1 | -0,937968 | -1,57058 | 3,13569 | 0 |  |
| 229043_at | PAPD5 | PAP associated domain containing 5 | -1,361425 | -1,14199 | 3,12927 | 0 |  |
| 226977_at | LOC492311 | similar to bovine IgA regulatory protein | -1,20265 | -1,29529 | 3,12243 | 0 |  |
| 37512_at | HSD17B6 | hydroxysteroid (17-beta) dehydrogenase 6 homolog (mouse) | -1,208742 | -1,27525 | 3,105 | 0 |  |
| 222906_at | FLVCR | feline leukemia virus subgroup C cellular receptor | -0,568941 | -1,91249 | 3,10178 | 0 |  |
| 225845_at | ZBTB44 | zinc finger and BTB domain containing 44 | -0,964041 | -1,51576 | 3,09976 | 0 |  |
| 229860_x_at | LOC401115 | hypothetical gene supported by BC038466; BC062790 | -1,207534 | -1,27221 | 3,09968 | 0 |  |
| 215716_s_at | ATP2B1 | ATPase, Ca++ transporting, plasma membrane 1 | -1,147432 | -1,3294 | 3,09604 | 0 |  |
| 227444_at | ARMCX4 | Armadillo repeat containing, X-linked 4 | -0,933126 | -1,53157 | 3,08087 | 0 |  |
| 225171_at | ARHGAP18 | Rho GTPase activating protein 18 | -0,727057 | -1,73698 | 3,08005 | 0 |  |
| 226431_at | ALS2CR13 | amyotrophic lateral sclerosis 2 (juvenile) chromosome region, candidate 13 | -1,5962 | -0,86597 | 3,07772 | 0 |  |
| 222273_at | PAPOLG | poly(A) polymerase gamma | -0,802056 | -1,65374 | 3,06974 | 0 |  |
| 202512_s_at | ATG5 | ATG5 autophagy related 5 homolog (S. cerevisiae) | -1,408466 | -1,04061 | 3,06135 | 0 |  |
| 208158_s_at | OSBPL1A | oxysterol binding protein-like 1A /// oxysterol binding protein-like 1A | -1,112462 | -1,33171 | 3,05521 | 0 |  |
| 219286_s_at | RBM15 | RNA binding motif protein 15 | -1,236787 | -1,20033 | 3,0464 | 0 |  |
| 204172_at | CPOX | coproporphyrinogen oxidase | -0,652144 | -1,78418 | 3,0454 | 0 |  |
| 212765_at | CAMSAP1L1 | calmodulin regulated spectrin-associated protein 1-like 1 | -1,031217 | -1,37441 | 3,00704 | 0 |  |
| 212761_at | TCF7L2 | transcription factor 7-like 2 (T-cell specific, HMG-box) | -1,209252 | -1,19574 | 3,00624 | 0 |  |
| 210093_s_at | MAGOH | mago-nashi homolog, proliferation-associated (Drosophila) | -0,861155 | -1,54264 | 3,00475 | 0 |  |
| 228006_at | PTEN | Phosphatase and tensin homolog | -1,698318 | -0,70437 | 3,00336 | 0 |  |
| 209459_s_at | ABAT | 4-aminobutyrate aminotransferase | -0,929896 | -1,47247 | 3,00296 | 0 |  |
| 231853_at | TUBD1 | tubulin, delta 1 | -0,793759 | -1,60856 | 3,00289 | 0 |  |
| 210111_s_at | KIAA0265 | KIAA0265 protein | -0,755841 | -1,63892 | 2,99346 | 0 |  |
| 203196_at | ABCC4 | ATP-binding cassette, sub-family C (CFTR/MRP), member 4 | -1,089337 | -1,30228 | 2,98952 | 0 |  |
| 224759_s_at | C12orf23 | chromosome 12 open reading frame 23 | -0,647987 | -1,74186 | 2,98731 | 0 |  |
| 228523_at | NANOS1 | nanos homolog 1 (Drosophila) | -1,273601 | -1,11461 | 2,98527 | 0 |  |
| 203906_at | IQSEC1 | IQ motif and Sec7 domain 1 | -0,890844 | -1,49266 | 2,97939 | 0 |  |
| 211458_s_at | GABARAPL1 /// GABARAPL3 | GABA(A) receptor-associated protein like 1 /// GABA(A) receptors associated protein like 3 | -1,343972 | -1,03601 | 2,97498 | 0 |  |
| 244455_at | KCNT2 | potassium channel, subfamily T, member 2 | -0,925297 | -1,45271 | 2,97251 | 0 |  |
| 226863_at | FAM110C | family with sequence similarity 110 member C | -0,832512 | -1,5435 | 2,97002 | 0 |  |
| 212190_at | SERPINE2 | serpin peptidase inhibitor, clade E (nexin, plasminogen activator inhibitor type 1), member 2 | -1,151278 | -1,21206 | 2,95417 | 0 |  |
| 202271_at | FBXO28 | F-box protein 28 | -0,877461 | -1,48249 | 2,94993 | 0 |  |
| 203069_at | SV2A | synaptic vesicle glycoprotein 2A | -1,453673 | -0,90326 | 2,94616 | 0 |  |
| 212900_at | SEC24A | SEC24 related gene family, member A (S. cerevisiae) | -0,779689 | -1,57433 | 2,94253 | 0 |  |
| 203044_at | CHSY1 | carbohydrate (chondroitin) synthase 1 | -1,244265 | -1,1057 | 2,93746 | 0 |  |
| 203323_at | CAV2 | caveolin 2 | -1,14903 | -1,19085 | 2,92485 | 0 |  |
| 237690_at | GPR115 | G protein-coupled receptor 115 | -1,189539 | -1,13633 | 2,90734 | 0 |  |
| 201088_at | KPNA2 /// LOC728860 | karyopherin alpha 2 (RAG cohort 1, importin alpha 1) /// similar to Importin alpha-2 subunit (Karyopherin alpha-2 subunit) (SRP1-alpha) (RAG cohort protein 1) | -0,755177 | -1,56279 | 2,89746 | 0 |  |
| 205281_s_at | PIGA | phosphatidylinositol glycan anchor biosynthesis, class A (paroxysmal nocturnal hemoglobinuria) /// phosphatidylinositol glycan anchor biosynthesis, class A (paroxysmal nocturnal hemoglobinuria) | -0,496617 | -1,81713 | 2,89219 | 0 |  |
| 226821_at | RIF1 | RAP1 interacting factor homolog (yeast) | -0,65381 | -1,65036 | 2,88021 | 0 |  |
| 228454_at | LCOR | ligand dependent nuclear receptor corepressor | -0,66144 | -1,63492 | 2,87045 | 0 |  |
| 225968_at | PRICKLE2 | prickle homolog 2 (Drosophila) | -1,175693 | -1,11023 | 2,8574 | 0 |  |
| 213090_s_at | TAF4 | TAF4 RNA polymerase II, TATA box binding protein (TBP)-associated factor, 135kDa | -0,93398 | -1,34824 | 2,85277 | 0 |  |
| 239300_at | PIK3C3 | Phosphoinositide-3-kinase, class 3 | -0,670121 | -1,60651 | 2,84579 | 0 |  |
| 228915_at | DACH1 | dachshund homolog 1 (Drosophila) | -1,573885 | -0,69663 | 2,83814 | 0 |  |
| 225910_at | LOC284019 | hypothetical protein LOC284019 | -1,244109 | -1,01922 | 2,82916 | 0 |  |
| 221556_at | CDC14B | CDC14 cell division cycle 14 homolog B (S. cerevisiae) | -0,748584 | -1,51358 | 2,8277 | 0 |  |
| 227777_at | C10orf18 | Chromosome 10 open reading frame 18 | -0,980106 | -1,28175 | 2,82732 | 0 |  |
| 39549_at | NPAS2 | neuronal PAS domain protein 2 | -0,961763 | -1,29983 | 2,82699 | 0 |  |
| 235648_at | ZNF567 | zinc finger protein 567 | -1,159846 | -1,09984 | 2,82461 | 0 |  |
| 229309_at | ADRB1 | adrenergic, beta-1-, receptor | -1,016234 | -1,23962 | 2,81981 | 0 |  |
| 205978_at | KL | klotho | -0,938426 | -1,31544 | 2,81733 | 0 |  |
| 229026_at | CDC42SE2 | CDC42 small effector 2 | -0,978768 | -1,27131 | 2,8126 | 0 |  |
| 204669_s_at | RNF24 | ring finger protein 24 | -0,192931 | -2,05518 | 2,81014 | 0 |  |
| 213793_s_at | HOMER1 | homer homolog 1 (Drosophila) | -0,804524 | -1,43904 | 2,80445 | 0 |  |
| 204731_at | TGFBR3 | transforming growth factor, beta receptor III (betaglycan, 300kDa) | -0,812908 | -1,42854 | 2,8018 | 0 |  |
| 227492_at | NAIP | similar to Occludin | -1,162961 | -1,07679 | 2,79969 | 0 |  |
| 208881_x_at | IDI1 | isopentenyl-diphosphate delta isomerase 1 | -0,531232 | -1,69224 | 2,77934 | 0 |  |
| 238035_at | SP3 | Sp3 transcription factor | -1,366667 | -0,85292 | 2,77448 | 0 |  |
| 203064_s_at | FOXK2 | forkhead box K2 | -0,694637 | -1,52129 | 2,76991 | 0 |  |
| 201196_s_at | AMD1 | adenosylmethionine decarboxylase 1 | -0,741789 | -1,46838 | 2,76271 | 0 |  |
| 227539_at | G-protein alpha-13 | Full-length cDNA clone CS0DB005YH06 of Neuroblastoma Cot 10-normalized of Homo sapiens (human) | -0,8899 | -1,31628 | 2,75773 | 0 |  |
| 241348_at | ZNF654 | zinc finger protein 654 | -0,951635 | -1,2396 | 2,73905 | 0 |  |
| 218087_s_at | SORBS1 | sorbin and SH3 domain containing 1 | -1,197228 | -0,99075 | 2,73497 | 0 |  |
| 225916_at | ZNF131 | zinc finger protein 131 | -0,869062 | -1,31213 | 2,72649 | 0 |  |
| 209866_s_at | LPHN3 | latrophilin 3 | -0,811538 | -1,36891 | 2,72556 | 0 |  |
| 209340_at | UAP1 | UDP-N-acteylglucosamine pyrophosphorylase 1 | -0,58422 | -1,59197 | 2,72024 | 0 |  |
| 215017_s_at | FNBP1L | formin binding protein 1-like | -0,727042 | -1,44787 | 2,71864 | 0 |  |
| 227859_at | RBJ | Ras-associated protein Rap1 | -0,156828 | -2,017 | 2,71729 | 0,06315 |  |
| 226225_at | MCC | mutated in colorectal cancers | -0,899157 | -1,27117 | 2,71291 | 0 |  |
| 203848_at | AKAP8 | A kinase (PRKA) anchor protein 8 | -1,064451 | -1,09726 | 2,70214 | 0 |  |
| 240592_at | LCORL | ligand dependent nuclear receptor corepressor-like | -1,074676 | -1,08647 | 2,70143 | 0 |  |
| 219251_s_at | WDR60 | WD repeat domain 60 | -0,601683 | -1,5593 | 2,70123 | 0 |  |
| 230029_x_at | ZNF650 | zinc finger protein 650 | -1,042051 | -1,10691 | 2,68621 | 0 |  |
| 228499_at | PFKFB4 | 6-phosphofructo-2-kinase/fructose-2,6-biphosphatase 4 | -0,929043 | -1,21217 | 2,67651 | 0 |  |
| 204719_at | ABCA8 | ATP-binding cassette, sub-family A (ABC1), member 8 | -1,075935 | -1,05963 | 2,66946 | 0 |  |
| 212274_at | LPIN1 | lipin 1 | -0,471334 | -1,65534 | 2,65834 | 0 |  |
| 227288_at | P18SRP | P18SRP protein | -1,147989 | -0,97709 | 2,65635 | 0 |  |
| 223288_at | USP38 | ubiquitin specific peptidase 38 | -0,872979 | -1,24602 | 2,64875 | 0 |  |
| 227282_at | PCDH19 | protocadherin 19 | -0,890948 | -1,22599 | 2,64617 | 0 |  |
| 204170_s_at | CKS2 | CDC28 protein kinase regulatory subunit 2 | -0,875636 | -1,24082 | 2,64558 | 0 |  |
| 204422_s_at | FGF2 | fibroblast growth factor 2 (basic) | -1,012431 | -1,10349 | 2,6449 | 0 |  |
| 209884_s_at | SLC4A7 | solute carrier family 4, sodium bicarbonate cotransporter, member 7 | -0,936501 | -1,17585 | 2,64044 | 0 |  |
| 209366_x_at | CYB5A | cytochrome b5 type A (microsomal) | -0,603209 | -1,50717 | 2,63797 | 0 |  |
| 238067_at | TBC1D8B | TBC1 domain family, member 8B (with GRAM domain) | -0,419041 | -1,68947 | 2,63564 | 0,06315 |  |
| 212710_at | CAMSAP1 | calmodulin regulated spectrin-associated protein 1 | -0,820575 | -1,28233 | 2,62863 | 0 |  |
| 230730_at | SGCD | sarcoglycan, delta (35kDa dystrophin-associated glycoprotein) | -1,107981 | -0,98825 | 2,62028 | 0,06315 |  |
| 221900_at | COL8A2 | collagen, type VIII, alpha 2 | -0,991524 | -1,0906 | 2,60266 | 0 |  |
| 235151_at | LOC283357 | hypothetical protein LOC283357 | -0,968436 | -1,10987 | 2,59789 | 0 |  |
| 227432_s_at | INSR | Insulin receptor | -0,586079 | -1,49223 | 2,59788 | 0 |  |
| 228562_at | ZBTB10 | Transcribed locus | -1,064017 | -1,008 | 2,59002 | 0 |  |
| 238436_s_at | ZNF805 | zinc finger protein 805 | -0,644939 | -1,42433 | 2,58659 | 0 |  |
| 239133_at | CTDSPL2 | CTD (carboxy-terminal domain, RNA polymerase II, polypeptide A) small phosphatase like 2 | -0,819401 | -1,2474 | 2,5835 | 0,06315 |  |
| 226682_at | LOC283666 | hypothetical protein LOC283666 | -0,635124 | -1,42929 | 2,58052 | 0 |  |
| 228280_at | ZC3HAV1L | zinc finger CCCH-type, antiviral 1-like | -0,761649 | -1,29883 | 2,5756 | 0 |  |
| 242100_at | CSS3 | chondroitin sulfate synthase 3 | -0,783349 | -1,2695 | 2,56606 | 0 |  |
| 225396_at | ZBTB8OS | Zinc finger and BTB domain containing 8 opposite strand | -0,745994 | -1,30672 | 2,56589 | 0,06315 |  |
| 223261_at | POLK | polymerase (DNA directed) kappa | -0,869628 | -1,18301 | 2,5658 | 0 |  |
| 242195_x_at | NUMBL | numb homolog (Drosophila)-like | -0,786643 | -1,26135 | 2,55999 | 0 |  |
| 225704_at | KIAA1545 | KIAA1545 protein | -1,027947 | -1,01125 | 2,549 | 0 |  |
| 209570_s_at | D4S234E | DNA segment on chromosome 4 (unique) 234 expressed sequence | -1,315384 | -0,71657 | 2,53994 | 0 |  |
| 229757_at | LOC92345 | Hypothetical protein BC008207 | -0,717265 | -1,31194 | 2,53651 | 0 |  |
| 225421_at | ACY1L2 | aminoacylase 1-like 2 | -0,567117 | -1,46096 | 2,5351 | 0 |  |
| 203075_at | SMAD2 | SMAD family member 2 | -0,733893 | -1,28712 | 2,52627 | 0 |  |
| 231964_at | BICD1 | MRNA; cDNA DKFZp564H1663 (from clone DKFZp564H1663) | -0,800466 | -1,21841 | 2,52359 | 0 |  |
| 244563_at | QSER1 | glutamine and serine rich 1 | -0,694791 | -1,32141 | 2,52025 | 0,33143 |  |
| 203642_s_at | COBLL1 | COBL-like 1 | -0,383679 | -1,63178 | 2,51932 | 0 |  |
| 214722_at | NOTCH2NL | Notch homolog 2 (Drosophila) N-terminal like | -1,047115 | -0,966 | 2,51639 | 0 |  |
| 225893_at | Roquin | Clone TESTIS-724 mRNA sequence | -0,924816 | -1,07607 | 2,50111 | 0 |  |
| 210997_at | HGF | hepatocyte growth factor (hepapoietin A; scatter factor) | 0,0899578 | -2,09052 | 2,5007 | 0 |  |
| 218940_at | C14orf138 | chromosome 14 open reading frame 138 | -0,938767 | -1,06107 | 2,49979 | 0,06315 |  |
| 207030_s_at | CSRP2 | cysteine and glycine-rich protein 2 | -0,72803 | -1,26447 | 2,49063 | 0 |  |
| 225633_at | DPY19L3 | dpy-19-like 3 (C. elegans) | -0,927695 | -1,06156 | 2,48657 | 0 |  |
| 1569040_s_at | FLJ40330 | similar to protein immuno-reactive with anti-PTH polyclonal antibodies | -1,314332 | -0,67402 | 2,48544 | 1,06427 |  |
| 219117_s_at | FKBP11 | FK506 binding protein 11, 19 kDa | -0,563977 | -1,41732 | 2,47662 | 0 |  |
| 221749_at | YTHDF3 | YTH domain family, member 3 | -0,846148 | -1,13349 | 2,47455 | 0 |  |
| 208760_at | UBE2I | Ubiquitin-conjugating enzyme E2I (UBC9 homolog, yeast) | -0,952274 | -1,02616 | 2,47304 | 0,06315 |  |
| 227866_at | LOC729436 | Hypothetical protein LOC729436 | -1,062157 | -0,91571 | 2,47233 | 0,25444 |  |
| 226370_at | KLHL15 | kelch-like 15 (Drosophila) | -1,135247 | -0,84101 | 2,47032 | 0 |  |
| 201303_at | EIF4A3 | eukaryotic translation initiation factor 4A, isoform 3 | -1,003711 | -0,97104 | 2,46844 | 0 |  |
| 224588_at | XIST | X (inactive)-specific transcript | -0,457195 | -1,51552 | 2,4659 | 0 |  |
| 214247_s_at | DKK3 | dickkopf homolog 3 (Xenopus laevis) | -0,655543 | -1,31541 | 2,46369 | 0 |  |
| 222449_at | TMEPAI | transmembrane, prostate androgen induced RNA | -1,098258 | -0,86596 | 2,45527 | 0 |  |
| 202798_at | SEC24B | SEC24 related gene family, member B (S. cerevisiae) | -0,688621 | -1,27269 | 2,45163 | 0 |  |
| 243463_s_at | RIT1 | Ras-like without CAAX 1 | -0,783549 | -1,17775 | 2,45163 | 0 |  |
| 226588_at | KIAA1604 | KIAA1604 protein | 0,2213217 | -2,18216 | 2,45105 | 0 |  |
| 222790_s_at | RSBN1 | round spermatid basic protein 1 | -0,432277 | -1,52782 | 2,45013 | 0 |  |
| 204396_s_at | GRK5 | G protein-coupled receptor kinase 5 | -0,905798 | -1,05408 | 2,44985 | 0 |  |
| 226003_at | KIF21A | kinesin family member 21A | -0,312676 | -1,64194 | 2,44327 | 0 |  |
| 221009_s_at | ANGPTL4 | angiopoietin-like 4 | -1,092329 | -0,85996 | 2,44036 | 0 |  |
| 217878_s_at | CDC27 | cell division cycle 27 homolog (S. cerevisiae) | -1,263909 | -0,68816 | 2,44009 | 0 |  |
| 208003_s_at | NFAT5 | nuclear factor of activated T-cells 5, tonicity-responsive | -0,699094 | -1,25174 | 2,43854 | 0 |  |
| 218847_at | IGF2BP2 | insulin-like growth factor 2 mRNA binding protein 2 | -0,68908 | -1,26153 | 2,43827 | 0 |  |
| 218421_at | CERK | ceramide kinase | -0,898582 | -1,04343 | 2,42752 | 0 |  |
| 214934_at | ATP9B | ATPase, Class II, type 9B | -0,862461 | -1,07407 | 2,42066 | 0 |  |
| 229376_at | PROX1 | Prospero homeobox 1 | -0,894975 | -1,0359 | 2,41359 | 0,11715 |  |
| 238199_x_at | LOC440552 | OK/SW-cl.16 | -0,850809 | -1,07914 | 2,41243 | 0 |  |
| 205241_at | SCO2 | SCO cytochrome oxidase deficient homolog 2 (yeast) | -0,570872 | -1,35676 | 2,40954 | 0 |  |
| 202219_at | SLC6A8 | solute carrier family 6 (neurotransmitter transporter, creatine), member 8 | -0,595526 | -1,33082 | 2,40793 | 0 |  |
| 230782_at | LOC653381 | similar to Sorbitol dehydrogenase (L-iditol 2-dehydrogenase) | -1,1303 | -0,79573 | 2,40754 | 0,06315 |  |
| 235542_at | MGC22014 | hypothetical protein MGC22014 | -0,521885 | -1,40179 | 2,40459 | 0 |  |
| 1556283_s_at | FGFR1OP2 | FGFR1 oncogene partner 2 | -1,033548 | -0,88925 | 2,40349 | 0 |  |
| 222731_at | ZDHHC2 | zinc finger, DHHC-type containing 2 | -0,970088 | -0,94715 | 2,39654 | 0 |  |
| 204500_s_at | AGTPBP1 | ATP/GTP binding protein 1 | -0,87704 | -1,038 | 2,3938 | 0 |  |
| 229590_at | RPL13 | Ribosomal protein L13 | -0,714497 | -1,19865 | 2,39143 | 0,06315 |  |
| 213166_x_at | FAM128B | family with sequence similarity 128, member B | -1,0512 | -0,86069 | 2,38986 | 0 |  |
| 203753_at | TCF4 | transcription factor 4 | -0,842098 | -1,06954 | 2,38955 | 0 |  |
| 212919_at | DCP2 | DCP2 decapping enzyme homolog (S. cerevisiae) | -0,678872 | -1,23231 | 2,38898 | 0 |  |
| 225188_at | RAPH1 | Ras association (RalGDS/AF-6) and pleckstrin homology domains 1 | -0,459903 | -1,45001 | 2,38739 | 0 |  |
| 218683_at | PTBP2 | polypyrimidine tract binding protein 2 | -0,849823 | -1,05241 | 2,37779 | 0,06315 |  |
| 238076_at | p66beta |  | -0,966351 | -0,93474 | 2,37636 | 0,11715 |  |
| 206105_at | AFF2 | AF4/FMR2 family, member 2 | -1,136739 | -0,7642 | 2,37617 | 0 |  |
| 202466_at | POLS | polymerase (DNA directed) sigma | -1,21337 | -0,68679 | 2,3752 | 0,06315 |  |
| 225290_at | ETNK1 | ethanolamine kinase 1 | -0,646936 | -1,2484 | 2,36917 | 0 |  |
| 225898_at | WDR54 | WD repeat domain 54 | -0,673519 | -1,21779 | 2,36414 | 0 |  |
| 209683_at | FAM49A | family with sequence similarity 49, member A | -0,637472 | -1,25305 | 2,36315 | 0 |  |
| 229513_at | STRBP | Spermatid perinuclear RNA binding protein | -0,81495 | -1,07221 | 2,35895 | 0,06315 |  |
| 205880_at | PRKD1 | protein kinase D1 | -1,298133 | -0,5866 | 2,35592 | 0 |  |
| 210053_at | TAF5 | TAF5 RNA polymerase II, TATA box binding protein (TBP)-associated factor, 100kDa | -0,282133 | -1,59957 | 2,35213 | 0,25444 |  |
| 229127_at | ATP5J | ATP synthase, H+ transporting, mitochondrial F0 complex, subunit F6 | -0,800321 | -1,08011 | 2,35054 | 0,11715 |  |
| 235170_at | ZNF92 | zinc finger protein 92 | -0,44906 | -1,4237 | 2,34095 | 0 |  |
| 226464_at | C3orf58 | chromosome 3 open reading frame 58 | -0,549259 | -1,32326 | 2,34064 | 0 |  |
| 227034_at | ANKRD57 | ankyrin repeat domain 57 | -0,817687 | -1,05396 | 2,33956 | 0 |  |
| 225540_at | MAP2 | microtubule-associated protein 2 | -0,968756 | -0,90018 | 2,33616 | 0 |  |
| 229377_at | GRTP1 | growth hormone regulated TBC protein 1 | -1,041038 | -0,82587 | 2,33363 | 0 |  |
| 201540_at | FHL1 | four and a half LIM domains 1 | -0,915183 | -0,95042 | 2,33201 | 0 |  |
| 221960_s_at | RAB2 | RAB2, member RAS oncogene family | -1,143818 | -0,71977 | 2,32949 | 0,33143 |  |
| 230098_at | PHF20L1 | PHD finger protein 20-like 1 | -0,957239 | -0,90624 | 2,32934 | 0,06315 |  |
| 1565823_at | Septin 7 | Transcribed locus | -1,19579 | -0,66608 | 2,32734 | 0,16318 |  |
| 219624_at | BAG4 | BCL2-associated athanogene 4 | -0,458032 | -1,39975 | 2,32223 | 1,06427 |  |
| 224763_at | RPL37 | Ribosomal protein L37 | -0,663352 | -1,19137 | 2,3184 | 0,33143 |  |
| 229624_at | OPA3 | Optic atrophy 3 (autosomal recessive, with chorea and spastic paraplegia) | -0,712711 | -1,13689 | 2,312 | 0,16318 |  |
| 224838_at | FOXP1 | forkhead box P1 | -0,872861 | -0,97495 | 2,30976 | 0 |  |
| 219157_at | KLHL2 | kelch-like 2, Mayven (Drosophila) | -0,676879 | -1,17021 | 2,30886 | 0 |  |
| 204573_at | CROT | carnitine O-octanoyltransferase | -0,683452 | -1,15761 | 2,30132 | 0 |  |
| 204393_s_at | ACPP | acid phosphatase, prostate | -0,35273 | -1,47747 | 2,28775 | 0 |  |
| 221794_at | DOCK6 | dedicator of cytokinesis 6 | -1,320809 | -0,50577 | 2,28323 | 0,16318 |  |
| 212927_at | SMC5 | Structural maintenance of chromosomes 5 | -0,874358 | -0,95183 | 2,28274 | 0 |  |
| 225181_at | ARID1B /// LOC729446 | AT rich interactive domain 1B (SWI1-like) /// similar to AT rich interactive domain 1B (SWI1-like) isoform 1 | -0,802099 | -1,02287 | 2,28121 | 0 |  |
| 225785_at | REEP3 | Receptor accessory protein 3 | -0,808151 | -1,01564 | 2,27974 | 0 |  |
| 213469_at | PGAP1 | GPI deacylase | -0,838595 | -0,98453 | 2,2789 | 0 |  |
| 214317_x_at | RPS9 | Ribosomal protein S9 | -0,831752 | -0,98945 | 2,27651 | 0 |  |
| 212665_at | TIPARP | TCDD-inducible poly(ADP-ribose) polymerase | -0,839896 | -0,98106 | 2,2762 | 0 |  |
| 227203_at | FBXL17 | F-box and leucine-rich repeat protein 17 | -0,590876 | -1,22985 | 2,27591 | 0 |  |
| 39966_at | CSPG5 | chondroitin sulfate proteoglycan 5 (neuroglycan C) | -1,016957 | -0,80297 | 2,27491 | 0,06315 |  |
| 205934_at | PLCL1 | phospholipase C-like 1 | -0,694384 | -1,12031 | 2,26837 | 0,06315 |  |
| 223467_at | RASD1 | RAS, dexamethasone-induced 1 | -0,992128 | -0,82203 | 2,2677 | 0 |  |
| 218181_s_at | MAP4K4 | mitogen-activated protein kinase kinase kinase kinase 4 | -0,631949 | -1,18128 | 2,26653 | 0 |  |
| 235278_at | C20orf133 | chromosome 20 open reading frame 133 | -0,535232 | -1,26978 | 2,25626 | 0,33143 |  |
| 224772_at | NAV1 | neuron navigator 1 | -1,156863 | -0,63511 | 2,23997 | 0,16318 |  |
| 235112_at | KIAA1958 | KIAA1958 | -0,365272 | -1,42664 | 2,23989 | 0 |  |
| 221014_s_at | RAB33B | RAB33B, member RAS oncogene family /// RAB33B, member RAS oncogene family | -0,789765 | -1,00073 | 2,23812 | 0,06315 |  |
| 225319_s_at | FAM104A | family with sequence similarity 104, member A | -0,798674 | -0,98831 | 2,23373 | 0 |  |
| 202235_at | SLC16A1 | solute carrier family 16, member 1 (monocarboxylic acid transporter 1) | -0,882778 | -0,89823 | 2,22626 | 0,25444 |  |
| 212456_at | KIAA0664 | KIAA0664 | -1,170464 | -0,60542 | 2,21985 | 0,06315 |  |
| 201843_s_at | EFEMP1 | EGF-containing fibulin-like extracellular matrix protein 1 | -0,809736 | -0,96583 | 2,21946 | 0 |  |
| 212538_at | DOCK9 | dedicator of cytokinesis 9 | -0,656171 | -1,1184 | 2,21821 | 0,16318 |  |
| 223216_x_at | ZNF395 /// FBXO16 | zinc finger protein 395 /// F-box protein 16 | -0,419813 | -1,35414 | 2,21745 | 0 |  |
| 1552897_a_at | KCNG3 | potassium voltage-gated channel, subfamily G, member 3 | -1,109737 | -0,66401 | 2,21718 | 0,33143 |  |
| 228220_at | FCHO2 | FCH domain only 2 | -0,575183 | -1,19785 | 2,2163 | 0,06315 |  |
| 231960_at | BRWD1 | bromodomain and WD repeat domain containing 1 | -0,79822 | -0,97203 | 2,21281 | 0,25444 |  |
| 226908_at | LRIG3 | leucine-rich repeats and immunoglobulin-like domains 3 | 0,2126197 | -1,98251 | 2,21237 | 0 |  |
| 228029_at | ZNF721 | zinc finger protein 721 | -0,813529 | -0,95514 | 2,21084 | 0,11715 |  |
| 222557_at | STMN3 | stathmin-like 3 | -0,834202 | -0,93334 | 2,20943 | 0 |  |
| 227059_at | GPC6 | Glypican 6 | -1,149934 | -0,61672 | 2,20832 | 0 |  |
| 1559827_at | LOC401074 | hypothetical LOC401074 | -0,810555 | -0,9559 | 2,20807 | 0,11715 |  |
| 202133_at | WWTR1 | WW domain containing transcription regulator 1 | -0,264457 | -1,50183 | 2,20786 | 0 |  |
| 221123_x_at | ZNF395 | zinc finger protein 395 | -0,314136 | -1,45145 | 2,20699 | 0 |  |
| 212565_at | STK38L | serine/threonine kinase 38 like | -0,717581 | -1,03916 | 2,19593 | 0,82259 |  |
| 204918_s_at | MLLT3 | myeloid/lymphoid or mixed-lineage leukemia (trithorax homolog, Drosophila); translocated to, 3 | -0,97474 | -0,78123 | 2,19497 | 0,60662 |  |
| 242794_at | MAML3 | mastermind-like 3 (Drosophila) | -1,01326 | -0,7411 | 2,19295 | 0,11715 |  |
| 223310_x_at | PNPLA8 | patatin-like phospholipase domain containing 8 | -0,479427 | -1,27217 | 2,18949 | 0 |  |
| 209120_at | NR2F2 | nuclear receptor subfamily 2, group F, member 2 | -0,915079 | -0,83303 | 2,18513 | 0 |  |
| 227533_at | RALGPS2 | Ral GEF with PH domain and SH3 binding motif 2 | -0,834365 | -0,91353 | 2,18486 | 0,11715 |  |
| 226392_at | RASA2 | RAS p21 protein activator 2 | -0,583315 | -1,16383 | 2,18393 | 0 |  |
| 210367_s_at | PTGES | prostaglandin E synthase | -0,257425 | -1,48669 | 2,18015 | 0 |  |
| 229830_at | PDGF-A | Transcribed locus | -0,922019 | -0,81908 | 2,17638 | 0,11715 |  |
| 231869_at | KIAA1586 | KIAA1586 | -0,634301 | -1,10676 | 2,17633 | 0,33143 |  |
| 232254_at | FBXO25 | F-box protein 25 | -0,722031 | -1,01779 | 2,17478 | 1,43745 |  |
| 213567_at | Karyopherin alpha 4 | Clone 23728 mRNA sequence | -0,897218 | -0,841 | 2,17277 | 0,11715 |  |
| 226208_at | ZSWIM6 | zinc finger, SWIM-type containing 6 | -0,690272 | -1,04747 | 2,17217 | 0 |  |
| 225813_at | MNAB | Membrane associated DNA binding protein | -0,3198 | -1,41022 | 2,16253 | 0 |  |
| 228098_s_at | MYLIP | myosin regulatory light chain interacting protein | -0,341759 | -1,38809 | 2,16231 | 0,60662 |  |
| 230570_at | eIF3S3 | Transcribed locus | -0,641237 | -1,08502 | 2,15782 | 1,06427 |  |
| 215093_at | NSDHL | NAD(P) dependent steroid dehydrogenase-like | -0,539003 | -1,18655 | 2,15695 | 0 |  |
| 239024_at | SLC12A8 | Solute carrier family 12 (potassium/chloride transporters), member 8 | -0,852372 | -0,86846 | 2,15104 | 0,33143 |  |
| 225223_at | SMAD5 | SMAD family member 5 | -0,970152 | -0,74659 | 2,14593 | 0,06315 |  |
| 235240_at | ATXN3 | Ataxin 3 | -0,142894 | -1,56751 | 2,138 | 0,16318 |  |
| 230657_at | CLOCK | Clock homolog (mouse) | -1,08508 | -0,62363 | 2,13589 | 0,33143 |  |
| 227621_at | SOD2 | Superoxide dismutase 2, mitochondrial | -0,589076 | -1,11843 | 2,13439 | 0 |  |
| 205862_at | GREB1 | GREB1 protein | -0,806911 | -0,89892 | 2,13229 | 0 |  |
| 218849_s_at | PPP1R13L | protein phosphatase 1, regulatory (inhibitor) subunit 13 like | -0,915304 | -0,78814 | 2,1293 | 0,06315 |  |
| 241392_at | TMEM39A | Transmembrane protein 39A | -0,80541 | -0,89387 | 2,1241 | 0,33143 |  |
| 1569107_s_at | ZNF642 | zinc finger protein 642 | -0,768812 | -0,92998 | 2,12349 | 1,90223 |  |
| 209894_at | LEPR | leptin receptor | -0,57029 | -1,12845 | 2,12343 | 0,43903 |  |
| 209263_x_at | TSPAN4 | tetraspanin 4 | -0,214245 | -1,48444 | 2,12335 | 0,06315 |  |
| 227200_at | ETV3 | Ets variant 3 | -0,548334 | -1,14993 | 2,12283 | 0,06315 |  |
| 224866_at | MLSTD2 | male sterility domain containing 2 | -0,627372 | -1,06862 | 2,11999 | 0,06315 |  |
| 238447_at | RBMS3 | RNA binding motif, single stranded interacting protein | -0,776569 | -0,9191 | 2,11959 | 0,06315 |  |
| 1556773_at | PTHLH | Parathyroid hormone-like hormone | -0,75644 | -0,93782 | 2,11783 | 0,06315 |  |
| 226335_at | RPS6KA3 | ribosomal protein S6 kinase, 90kDa, polypeptide 3 | -0,214109 | -1,47983 | 2,11742 | 0 |  |
| 203365_s_at | MMP15 | matrix metallopeptidase 15 (membrane-inserted) | -0,948901 | -0,74295 | 2,11482 | 0,11715 |  |
| 217986_s_at | BAZ1A | bromodomain adjacent to zinc finger domain, 1A | -0,895485 | -0,79593 | 2,11426 | 0,06315 |  |
| 201626_at | INSIG1 | insulin induced gene 1 | -0,268124 | -1,4228 | 2,11366 | 0 |  |
| 235165_at | PARD6B | par-6 partitioning defective 6 homolog beta (C. elegans) | -0,418719 | -1,26543 | 2,10519 | 1,06427 |  |
| 1552789_at | TLOC1 | translocation protein 1 | -0,709346 | -0,97274 | 2,10261 | 1,43745 |  |
| 222077_s_at | RACGAP1 | Rac GTPase activating protein 1 | -0,646727 | -1,03342 | 2,10018 | 0,25444 |  |
| 227105_at | CSPP1 | centrosome and spindle pole associated protein 1 | -0,351229 | -1,32892 | 2,10018 | 0,16318 |  |
| 222416_at | ALDH18A1 | aldehyde dehydrogenase 18 family, member A1 | -0,54229 | -1,13687 | 2,09896 | 0,06315 |  |
| 219129_s_at | SAP30L | SAP30-like | -0,568168 | -1,10645 | 2,09327 | 0,33143 |  |
| 200071_at | SMNDC1 | survival motor neuron domain containing 1 /// survival motor neuron domain containing 1 | -0,883027 | -0,79128 | 2,09289 | 0,06315 |  |
| 228967_at | EIF1 | Eukaryotic translation initiation factor 1 | -0,449189 | -1,21644 | 2,08204 | 0,06315 |  |
| 1561286_a_at | DIP2A | DIP2 disco-interacting protein 2 homolog A (Drosophila) | -0,51102 | -1,15393 | 2,08119 | 0,60662 |  |
| 213310_at | EIF2C2 | Eukaryotic translation initiation factor 2C, 2 | -1,027198 | -0,63609 | 2,07911 | 0,33143 |  |
| 200884_at | CKB | creatine kinase, brain | -0,887209 | -0,77577 | 2,07872 | 0 |  |
| 216550_x_at | ANKRD12 | ankyrin repeat domain 12 | -0,759161 | -0,90196 | 2,0764 | 0,11715 |  |
| 217824_at | UBE2J1 | ubiquitin-conjugating enzyme E2, J1 (UBC6 homolog, yeast) | -0,435796 | -1,22473 | 2,07565 | 0,33143 |  |
| 229160_at | MUM1L1 | melanoma associated antigen (mutated) 1-like 1 | -1,240415 | -0,41877 | 2,07398 | 0 |  |
| 213610_s_at | KLHL23 | kelch-like 23 (Drosophila) | -0,705739 | -0,95192 | 2,07208 | 0,11715 |  |
| 1554455_at | LINS1 | lines homolog 1 (Drosophila) | -0,587798 | -1,06865 | 2,07056 | 0,60662 |  |
| 200880_at | DNAJA1 | DnaJ (Hsp40) homolog, subfamily A, member 1 | -0,393171 | -1,25963 | 2,066 | 0 |  |
| 225293_at | COL27A1 | collagen, type XXVII, alpha 1 | -0,533133 | -1,11792 | 2,06382 | 0,33143 |  |
| 200635_s_at | PTPRF | protein tyrosine phosphatase, receptor type, F | -0,538358 | -1,1126 | 2,0637 | 0,06315 |  |
| 228565_at | KIAA1804 | mixed lineage kinase 4 | -0,222605 | -1,42677 | 2,06172 | 0,06315 |  |
| 222494_at | CHES1 | checkpoint suppressor 1 | -0,925515 | -0,7229 | 2,06052 | 0,06315 |  |
| 235591_at | SSTR1 | somatostatin receptor 1 | -0,755651 | -0,89007 | 2,05715 | 0,11715 |  |
| 225989_at | HERC4 | hect domain and RLD 4 | 0,1494867 | -1,79201 | 2,05316 | 0 |  |
| 212609_s_at | AKT3 | V-akt murine thymoma viral oncogene homolog 3 (protein kinase B, gamma) | -0,857806 | -0,78085 | 2,04832 | 0,16318 |  |
| 235015_at | ZDHHC9 | Full-length cDNA clone CS0DI071YF17 of Placenta Cot 25-normalized of Homo sapiens (human) | -0,376089 | -1,26221 | 2,04787 | 0,43903 |  |
| 209153_s_at | TCF3 | transcription factor 3 (E2A immunoglobulin enhancer binding factors E12/E47) | -0,704531 | -0,93039 | 2,04365 | 0,25444 |  |
| 213212_x_at | LOC161527 /// FLJ40113 /// LOC642346 /// LOC643696 /// LOC728636 | hypothetical protein LOC161527 /// golgi autoantigen, golgin subfamily a-like pseudogene /// similar to golgi autoantigen, golgin subfamily a-like /// similar to golgi autoantigen, golgin subfamily a-like /// similar to golgi autoantigen, golgin subfamily | -0,262971 | -1,37139 | 2,04295 | 0,06315 |  |
| 213024_at | TMF1 | TATA element modulatory factor 1 | -0,59204 | -1,03498 | 2,03377 | 0,16318 |  |
| 204824_at | ENDOG | endonuclease G | -0,763542 | -0,86207 | 2,03202 | 0,16318 |  |
| 235683_at | SESN3 | sestrin 3 | -0,841273 | -0,78398 | 2,03157 | 2,53333 |  |
| 207345_at | FST | follistatin | -0,318149 | -1,30301 | 2,02644 | 0 |  |
| 236236_at | WNK3 | CDNA FLJ30437 fis, clone BRACE2009045 | -0,619968 | -0,99987 | 2,0248 | 0,06315 |  |
| 204206_at | MNT | MAX binding protein | -0,637845 | -0,9758 | 2,01706 | 0,06315 |  |
| 224952_at | TANC2 | tetratricopeptide repeat, ankyrin repeat and coiled-coil containing 2 | -0,693395 | -0,91841 | 2,01476 | 0,06315 |  |
| 225367_at | PGM2 | phosphoglucomutase 2 | -0,504849 | -1,10627 | 2,0139 | 0,06315 |  |
| 217842_at | LUC7L2 | LUC7-like 2 (S. cerevisiae) | -0,555607 | -1,05442 | 2,01254 | 0,43903 |  |
| 203271_s_at | UNC119 | unc-119 homolog (C. elegans) | -0,366592 | -1,24279 | 2,01173 | 0,33143 |  |
| 1555259_at | ZAK | sterile alpha motif and leucine zipper containing kinase AZK | -0,938884 | -0,6703 | 2,01148 | 0,33143 |  |
| 230141_at | ARID4A | AT rich interactive domain 4A (RBP1-like) | -0,505602 | -1,0971 | 2,00337 | 0,60662 |  |
| 1555579_s_at | PTPRM | protein tyrosine phosphatase, receptor type, M | -0,515745 | -1,08165 | 1,99674 | 0,06315 |  |
| 226899_at | UNC5B | unc-5 homolog B (C. elegans) | -0,459624 | -1,13557 | 1,994 | 0,11715 |  |
| 222149_x_at | GOLGA8G | golgi autoantigen, golgin subfamily a, 8G | -1,364556 | -0,22574 | 1,98788 | 0,60662 |  |
| 235620_x_at | ZMYM5 | zinc finger, MYM-type 5 | -0,714748 | -0,87553 | 1,98785 | 0,82259 |  |
| 200781_s_at | RPS15A | ribosomal protein S15a | -0,700621 | -0,88913 | 1,98719 | 0,06315 |  |
| 201849_at | BNIP3 | BCL2/adenovirus E1B 19kDa interacting protein 3 | -0,109154 | -1,47969 | 1,98605 | 0 |  |
| 219093_at | FLJ20701 | hypothetical protein FLJ20701 /// hypothetical protein FLJ20701 | -0,570094 | -1,0166 | 1,98337 | 0,11715 |  |
| 36829_at | PER1 | period homolog 1 (Drosophila) | -1,16065 | -0,4253 | 1,98244 | 0,16318 |  |
| 243423_at | TNIP1 | Transcribed locus | -1,327068 | -0,25876 | 1,98229 | 0,33143 |  |
| 204435_at | NUPL1 | nucleoporin like 1 | -0,553971 | -1,02937 | 1,97918 | 0,11715 |  |
| 225196_s_at | MRPS26 | mitochondrial ribosomal protein S26 | -0,560495 | -1,02149 | 1,97748 | 0,25444 |  |
| 226345_at | ARL5B | CDNA FLJ12853 fis, clone NT2RP2003456 | -0,627825 | -0,95245 | 1,97535 | 0,11715 |  |
| 228993_s_at | LOC92482 | hypothetical protein LOC92482 | -0,787461 | -0,79142 | 1,9736 | 0,11715 |  |
| 200799_at | HSPA1A | heat shock 70kDa protein 1A | -0,247603 | -1,3281 | 1,96963 | 0 |  |
| 238065_at | TPM3 | tropomyosin 3 | -0,726726 | -0,84661 | 1,96666 | 1,43745 |  |
| 235228_at | CCDC85A | coiled-coil domain containing 85A | -0,46054 | -1,11144 | 1,96498 | 0,33143 |  |
| 221510_s_at | GLS | glutaminase | -0,499775 | -1,07188 | 1,96456 | 0,06315 |  |
| 34858_at | KCTD2 | potassium channel tetramerisation domain containing 2 | -0,632615 | -0,93851 | 1,96391 | 0,33143 |  |
| 235556_at | LOC153222 | Adult retina protein | -0,6987 | -0,86923 | 1,95991 | 0,33143 |  |
| 235125_x_at | FAM73A | family with sequence similarity 73, member A | -0,77671 | -0,79094 | 1,95956 | 0,43903 |  |
| 204639_at | ADA | adenosine deaminase | -0,128847 | -1,43782 | 1,95833 | 0,06315 |  |
| 228145_s_at | ZNF398 | zinc finger protein 398 | -0,67695 | -0,88716 | 1,95514 | 0,43903 |  |
| 209946_at | VEGFC | vascular endothelial growth factor C | -0,497706 | -1,06554 | 1,95405 | 0,16318 |  |
| 205829_at | HSD17B1 | hydroxysteroid (17-beta) dehydrogenase 1 | -0,325731 | -1,23435 | 1,95011 | 0,06315 |  |
| 220251_at | C1orf107 | chromosome 1 open reading frame 107 | -1,598119 | 0,04158 | 1,94568 | 0,60662 |  |
| 230100_x_at | PAK1 | p21/Cdc42/Rac1-activated kinase 1 (STE20 homolog, yeast) | -0,965136 | -0,59111 | 1,94531 | 0,43903 |  |
| 225342_at | AK3L1 | adenylate kinase 3-like 1 | -0,474939 | -1,07834 | 1,9416 | 0,06315 |  |
| 213048_s_at | SET | SET translocation (myeloid leukemia-associated) | -0,784704 | -0,76803 | 1,94092 | 0,11715 |  |
| 221566_s_at | NOL3 | nucleolar protein 3 (apoptosis repressor with CARD domain) | -0,748445 | -0,79859 | 1,93379 | 0,25444 |  |
| 222237_s_at | ZNF228 | zinc finger protein 228 | -0,983923 | -0,5615 | 1,93178 | 0,82259 |  |
| 232165_at | EPPK1 | epiplakin 1 | -1,3866 | -0,1562 | 1,9285 | 0,33143 |  |
| 218723_s_at | RGC32 | response gene to complement 32 | -0,456903 | -1,08579 | 1,92837 | 0,11715 |  |
| 203917_at | CXADR | coxsackie virus and adenovirus receptor | -0,091883 | -1,44954 | 1,92678 | 0,06315 |  |
| 224910_at | CARHSP1 | calcium regulated heat stable protein 1, 24kDa | -0,278344 | -1,26295 | 1,92662 | 0,25444 |  |
| 226069_at | PRICKLE1 | prickle homolog 1 (Drosophila) | -1,112146 | -0,42907 | 1,92651 | 1,90223 |  |
| 228477_at | FLJ10154 | Hypothetical protein FLJ10154 | -0,548742 | -0,98968 | 1,92303 | 0,11715 |  |
| 213451_x_at | TNXB | tenascin XB | -0,709062 | -0,82858 | 1,92205 | 1,06427 |  |
| 207738_s_at | NCKAP1 | NCK-associated protein 1 | -0,535849 | -1,00142 | 1,92159 | 0,06315 |  |
| 202540_s_at | HMGCR | 3-hydroxy-3-methylglutaryl-Coenzyme A reductase | -0,092069 | -1,44426 | 1,92041 | 0 |  |
| 202850_at | ABCD3 | ATP-binding cassette, sub-family D (ALD), member 3 | -0,695014 | -0,84107 | 1,9201 | 0,11715 |  |
| 213136_at | PTPN2 | protein tyrosine phosphatase, non-receptor type 2 | -0,322858 | -1,2114 | 1,91783 | 0,16318 |  |
| 213452_at | ZNF184 | zinc finger protein 184 | -0,499824 | -1,03346 | 1,91661 | 1,06427 |  |
| 222447_at | METTL9 | methyltransferase like 9 | -0,492299 | -1,03658 | 1,9111 | 0,82259 |  |
| 213686_at | VPS13A | Vacuolar protein sorting 13 homolog A (S. cerevisiae) | -0,791541 | -0,73662 | 1,91021 | 1,90223 |  |
| 213664_at | SLC1A1 | solute carrier family 1 (neuronal/epithelial high affinity glutamate transporter, system Xag), member 1 | -0,411991 | -1,11596 | 1,90994 | 0,82259 |  |
| 212221_x_at | IDS | iduronate 2-sulfatase (Hunter syndrome) | -0,569847 | -0,95793 | 1,90972 | 0,11715 |  |
| 243539_at | KIAA1841 | KIAA1841 | -0,784464 | -0,73948 | 1,90493 | 0,82259 |  |
| 218035_s_at | FLJ20273 | RNA-binding protein | -0,490296 | -1,02893 | 1,89903 | 0,11715 |  |
| 201810_s_at | SH3BP5 | SH3-domain binding protein 5 (BTK-associated) | -0,972591 | -0,54659 | 1,89898 | 0,11715 |  |
| 219213_at | JAM2 | junctional adhesion molecule 2 | -0,841523 | -0,67759 | 1,89889 | 0,60662 |  |
| 226099_at | ELL2 | elongation factor, RNA polymerase II, 2 | -0,641167 | -0,87682 | 1,89749 | 0,11715 |  |
| 228471_at | NEIL1 /// ANKRD44 | Nei endonuclease VIII-like 1 (E. coli) /// Ankyrin repeat domain 44 | -1,056763 | -0,46107 | 1,89729 | 0,43903 |  |
| 213446_s_at | IQGAP1 | IQ motif containing GTPase activating protein 1 | -1,072088 | -0,44571 | 1,89725 | 0,16318 |  |
| 227426_at | SOS1 | Transcribed locus | -0,445988 | -1,07158 | 1,89696 | 0,43903 |  |
| 222819_at | CTPS2 | CTP synthase II | -0,40874 | -1,10025 | 1,88624 | 0,82259 |  |
| 230380_at | THAP2 | THAP domain containing, apoptosis associated protein 2 | -0,600326 | -0,90792 | 1,8853 | 0,25444 |  |
| 207002_s_at | PLAGL1 | pleiomorphic adenoma gene-like 1 | -0,254896 | -1,25221 | 1,88388 | 0,11715 |  |
| 218626_at | EIF4ENIF1 | eukaryotic translation initiation factor 4E nuclear import factor 1 | -0,709073 | -0,79351 | 1,87823 | 0,25444 |  |
| 200815_s_at | PAFAH1B1 | platelet-activating factor acetylhydrolase, isoform Ib, alpha subunit 45kDa | -0,418136 | -1,08213 | 1,87533 | 0,25444 |  |
| 226077_at | FLJ31951 | hypothetical protein FLJ31951 | -0,289249 | -1,209 | 1,87281 | 0,06315 |  |
| 202499_s_at | SLC2A3 | solute carrier family 2 (facilitated glucose transporter), member 3 | -0,750365 | -0,74755 | 1,8724 | 0,16318 |  |
| 213178_s_at | MAPK8IP3 | mitogen-activated protein kinase 8 interacting protein 3 | -0,569792 | -0,92783 | 1,87202 | 0,43903 |  |
| 225814_at | XRN1 | 5'-3' exoribonuclease 1 | -0,759443 | -0,7358 | 1,86905 | 0,43903 |  |
| 207725_at | POU4F2 | POU domain, class 4, transcription factor 2 | -0,085141 | -1,40959 | 1,86842 | 0,25444 |  |
| 226023_at | MAP2K7 | mitogen-activated protein kinase kinase 7 | -1,218804 | -0,27411 | 1,86614 | 1,06427 |  |
| 229114_at | GAB1 | CDNA clone IMAGE:4801326 | -0,876666 | -0,61595 | 1,86577 | 1,43745 |  |
| 224345_x_at | C3orf28 | chromosome 3 open reading frame 28 /// chromosome 3 open reading frame 28 | -0,346198 | -1,14477 | 1,8637 | 0,06315 |  |
| 225768_at | NR1D2 | nuclear receptor subfamily 1, group D, member 2 | -0,66104 | -0,82753 | 1,86071 | 0,25444 |  |
| 227176_at | SLC2A13 | solute carrier family 2 (facilitated glucose transporter), member 13 | -0,573929 | -0,91395 | 1,85985 | 0,43903 |  |
| 205560_at | PCSK5 | proprotein convertase subtilisin/kexin type 5 | -0,34688 | -1,14025 | 1,85892 | 0,33143 |  |
| 230930_at | LOC338620 | hypothetical protein LOC338620 | -1,203819 | -0,28311 | 1,85866 | 0,33143 |  |
| 211018_at | LSS | lanosterol synthase (2,3-oxidosqualene-lanosterol cyclase) | -1,134931 | -0,35149 | 1,85803 | 0,82259 |  |
| 219230_at | TMEM100 | transmembrane protein 100 | -0,98669 | -0,49658 | 1,85409 | 1,43745 |  |
| 220161_s_at | EPB41L4B | erythrocyte membrane protein band 4.1 like 4B | -0,762707 | -0,718 | 1,85088 | 0,25444 |  |
| 1554628_at | ZNF57 | zinc finger protein 57 | -0,848084 | -0,6326 | 1,85086 | 0,82259 |  |
| 214683_s_at | CLK1 | CDC-like kinase 1 | -0,530225 | -0,95012 | 1,85042 | 0,33143 |  |
| 227345_at | TNFRSF10D | tumor necrosis factor receptor superfamily, member 10d, decoy with truncated death domain | -0,673376 | -0,80615 | 1,84941 | 1,06427 |  |
| 238462_at | STS-1 | Cbl-interacting protein Sts-1 | -0,206836 | -1,27112 | 1,84744 | 0,33143 |  |
| 209211_at | KLF5 | Kruppel-like factor 5 (intestinal) | -0,275625 | -1,2003 | 1,8449 | 0,16318 |  |
| 205747_at | CBLN1 | cerebellin 1 precursor | -1,049134 | -0,42588 | 1,84377 | 0,43903 |  |
| 202923_s_at | GCLC | glutamate-cysteine ligase, catalytic subunit | -0,511584 | -0,96131 | 1,84112 | 0,16318 |  |
| 228925_at | TMEM116 | Transmembrane protein 116 | -0,110643 | -1,36141 | 1,84006 | 1,90223 |  |
| 228401_at | ATAD2 | ATPase family, AAA domain containing 2 | -1,087324 | -0,38346 | 1,83847 | 0,33143 |  |
| 238865_at | LOC132430 | similar to poly(A) binding protein, cytoplasmic 4 (inducible form) | -0,822594 | -0,6471 | 1,83712 | 4,2202 |  |
| 225735_at | ANKRD50 | ankyrin repeat domain 50 | -0,901696 | -0,56756 | 1,83657 | 1,43745 |  |
| 221974_at | PWCR1 | Prader-Willi syndrome chromosome region 1 | -0,991897 | -0,47619 | 1,8351 | 0,33143 |  |
| 231017_at | STK11 | serine/threonine kinase 11 | -0,583154 | -0,8839 | 1,83382 | 1,06427 |  |
| 203407_at | PPL | periplakin | -0,423436 | -1,04207 | 1,83188 | 0,60662 |  |
| 203282_at | GBE1 | glucan (1,4-alpha-), branching enzyme 1 (glycogen branching enzyme, Andersen disease, glycogen storage disease type IV) | -0,503645 | -0,96115 | 1,831 | 0,16318 |  |
| 217901_at | DSG2 | Desmoglein 2 | -0,843783 | -0,62058 | 1,83045 | 0,25444 |  |
| 230777_s_at | PRDM15 /// LOC728766 | PR domain containing 15 /// similar to PR domain containing 15 | -0,657747 | -0,80647 | 1,83027 | 0,25444 |  |
| 236128_at | ZNF91 | zinc finger protein 91 | -0,541205 | -0,92291 | 1,83014 | 1,06427 |  |
| 241399_at | FAM19A2 | family with sequence similarity 19 (chemokine (C-C motif)-like), member A2 | -0,506423 | -0,95657 | 1,82874 | 2,53333 |  |
| 226043_at | GPSM1 | G-protein signalling modulator 1 (AGS3-like, C. elegans) | -0,440468 | -1,02248 | 1,82868 | 0,25444 |  |
| 226625_at | TGF-beta receptor type III (betaglycan) |  | -0,385592 | -1,07593 | 1,8269 | 0,11715 |  |
| 226650_at | ZFAND2A | zinc finger, AN1-type domain 2A | -0,112096 | -1,34765 | 1,82469 | 0,16318 |  |
| 209625_at | PIGH | phosphatidylinositol glycan anchor biosynthesis, class H | -0,800682 | -0,65806 | 1,82343 | 0,60662 |  |
| 242828_at | FIGN | Fidgetin | -0,782995 | -0,67398 | 1,82122 | 1,06427 |  |
| 214829_at | AASS | aminoadipate-semialdehyde synthase | -0,355032 | -1,09901 | 1,81756 | 0,60662 |  |
| 214053_at | ERBB4 | v-erb-a erythroblastic leukemia viral oncogene homolog 4 (avian) | -0,810077 | -0,64385 | 1,81741 | 1,90223 |  |
| 218371_s_at | PSPC1 | paraspeckle component 1 | -0,914622 | -0,53609 | 1,81339 | 1,43745 |  |
| 209422_at | PHF20 | PHD finger protein 20 | -0,332681 | -1,11612 | 1,811 | 0,25444 |  |
| 242245_at | SYDE2 | Synapse defective 1, Rho GTPase, homolog 2 (C. elegans) | -0,578437 | -0,87035 | 1,81098 | 1,43745 |  |
| 201965_s_at | SETX | senataxin | -0,560932 | -0,88583 | 1,80845 | 0,60662 |  |
| 205109_s_at | ARHGEF4 | Rho guanine nucleotide exchange factor (GEF) 4 | -0,803645 | -0,64277 | 1,80802 | 0,33143 |  |
| 217073_x_at | APOA1 | apolipoprotein A-I | -0,496212 | -0,94975 | 1,80746 | 0,33143 |  |
| 1553392_at | EFCAB3 | EF-hand calcium binding domain 3 | -0,916717 | -0,52724 | 1,80495 | 2,53333 |  |
| 224897_at | WDR26 | WD repeat domain 26 | -0,77976 | -0,66414 | 1,80488 | 0,60662 |  |
| 232033_at | USP37 | ubiquitin specific peptidase 37 | -0,456654 | -0,98716 | 1,80477 | 1,90223 |  |
| 226750_at | LARP2 | La ribonucleoprotein domain family, member 2 | -0,138937 | -1,30453 | 1,80434 | 0,82259 |  |
| 205191_at | RP2 | retinitis pigmentosa 2 (X-linked recessive) | -0,193755 | -1,24907 | 1,80353 | 0,43903 |  |
| 218985_at | SLC2A8 | solute carrier family 2, (facilitated glucose transporter) member 8 | -0,446064 | -0,9966 | 1,80333 | 1,90223 |  |
| 202492_at | ATG9A | ATG9 autophagy related 9 homolog A (S. cerevisiae) | -1,27522 | -0,16521 | 1,80054 | 0,60662 |  |
| 226641_at | ANKRD44 | Ankyrin repeat domain 44 | -0,831724 | -0,60392 | 1,79455 | 0,60662 |  |
| 205527_s_at | GEMIN4 | gem (nuclear organelle) associated protein 4 | -0,266037 | -1,16862 | 1,79332 | 1,43745 |  |
| 1558404_at | LOC644242 | Hypothetical protein LOC644242 | -0,604 | -0,82836 | 1,79046 | 0,43903 |  |
| 229819_at | A1BG | alpha-1-B glycoprotein | -0,911524 | -0,51977 | 1,78911 | 1,43745 |  |
| 214719_at | LOC283537 | hypothetical protein LOC283537 | -1,111778 | -0,31805 | 1,78728 | 0,16318 |  |
| 227376_at | GLI3 = GLI-3R | Transcribed locus | -1,369332 | -0,05909 | 1,78552 | 0,16318 |  |
| 228131_at | ERCC1 | Excision repair cross-complementing rodent repair deficiency, complementation group 1 (includes overlapping antisense sequence) | -0,779144 | -0,64901 | 1,7852 | 1,43745 |  |
| 231111_at | C1orf112 | Chromosome 1 open reading frame 112 | -0,787649 | -0,64031 | 1,78495 | 3,25444 |  |
| 204748_at | PTGS2 | prostaglandin-endoperoxide synthase 2 (prostaglandin G/H synthase and cyclooxygenase) | -0,637939 | -0,78838 | 1,7829 | 0,25444 |  |
| 203789_s_at | SEMA3C | sema domain, immunoglobulin domain (Ig), short basic domain, secreted, (semaphorin) 3C | -0,517974 | -0,90826 | 1,78279 | 0,25444 |  |
| 218326_s_at | LGR4 | leucine-rich repeat-containing G protein-coupled receptor 4 | -0,452984 | -0,97158 | 1,78071 | 0,25444 |  |
| 203413_at | NELL2 | NEL-like 2 (chicken) /// NEL-like 2 (chicken) | -0,491787 | -0,93276 | 1,78068 | 0,33143 |  |
| 213085_s_at | WWC1 | WW and C2 domain containing 1 | -0,487759 | -0,93348 | 1,77654 | 1,43745 |  |
| 1556239_a_at | HERPUD2 | HERPUD family member 2 | -0,671935 | -0,74893 | 1,77609 | 2,53333 |  |
| 226217_at | SLC30A7 | solute carrier family 30 (zinc transporter), member 7 | -0,365309 | -1,05294 | 1,77281 | 0,25444 |  |
| 1556228_a_at | VCPIP1 | Valosin containing protein (p97)/p47 complex interacting protein 1 | -0,940617 | -0,47742 | 1,77255 | 1,43745 |  |
| 225306_s_at | SLC25A29 | solute carrier family 25, member 29 | -0,194222 | -1,22373 | 1,77243 | 1,06427 |  |
| 235850_at | WDR5B | WD repeat domain 5B | -0,366089 | -1,0517 | 1,77224 | 1,06427 |  |
| 230069_at | SFXN1 | sideroflexin 1 | -0,762368 | -0,65481 | 1,77148 | 1,06427 |  |
| 227148_at | PLEKHH2 | pleckstrin homology domain containing, family H (with MyTH4 domain) member 2 | -0,902655 | -0,51282 | 1,76935 | 0,82259 |  |
| 226649_at | PANK1 | pantothenate kinase 1 | -0,729035 | -0,68508 | 1,76764 | 1,90223 |  |
| 1556151_at | ITFG1 | Integrin alpha FG-GAP repeat containing 1 | -0,993967 | -0,41932 | 1,76661 | 0,25444 |  |
| 236192_at | FNTA | Farnesyltransferase, CAAX box, alpha | -0,43238 | -0,97998 | 1,76545 | 0,82259 |  |
| 202620_s_at | PLOD2 | procollagen-lysine, 2-oxoglutarate 5-dioxygenase 2 | -0,434527 | -0,97571 | 1,7628 | 0,25444 |  |
| 231931_at | PRDM15 | PR domain containing 15 | -0,428876 | -0,98083 | 1,76213 | 0,43903 |  |
| 219062_s_at | ZCCHC2 | zinc finger, CCHC domain containing 2 | -0,034887 | -1,3748 | 1,76212 | 0,33143 |  |
| 223584_s_at | KBTBD2 | kelch repeat and BTB (POZ) domain containing 2 | -0,698508 | -0,71115 | 1,76207 | 2,53333 |  |
| 213935_at | ABHD5 | abhydrolase domain containing 5 | -0,320802 | -1,08857 | 1,76172 | 0,82259 |  |
| 219676_at | ZNF435 | zinc finger protein 435 | -0,887003 | -0,51651 | 1,75439 | 3,25444 |  |
| 222633_at | TBL1XR1 | transducin (beta)-like 1X-linked receptor 1 | -0,526217 | -0,87679 | 1,75376 | 0,33143 |  |
| 226832_at | RNF168 /// LOC727900 | Ring finger protein 168 /// Hypothetical protein LOC727900 | -0,37498 | -1,0278 | 1,75348 | 0,43903 |  |
| 212334_at | GNS | glucosamine (N-acetyl)-6-sulfatase (Sanfilippo disease IIID) | -0,722821 | -0,67929 | 1,75263 | 0,33143 |  |
| 218840_s_at | NADSYN1 | NAD synthetase 1 | -0,554051 | -0,8479 | 1,75244 | 1,90223 |  |
| 222162_s_at | ADAMTS1 | ADAM metallopeptidase with thrombospondin type 1 motif, 1 | -0,296677 | -1,10212 | 1,7485 | 0,16318 |  |
| 215499_at | MAP2K3 | mitogen-activated protein kinase kinase 3 /// mitogen-activated protein kinase kinase 3 | -0,691136 | -0,70489 | 1,74504 | 0,82259 |  |
| 212192_at | KCTD12 | potassium channel tetramerisation domain containing 12 | -0,972146 | -0,4213 | 1,7418 | 0,25444 |  |
| 204457_s_at | GAS1 | growth arrest-specific 1 | -0,487317 | -0,90467 | 1,73998 | 0,25444 |  |
| 218889_at | NOC3L | nucleolar complex associated 3 homolog (S. cerevisiae) | -0,316528 | -1,07347 | 1,73749 | 0,43903 |  |
| 213286_at | ZFR | zinc finger RNA binding protein | -0,749822 | -0,63567 | 1,73187 | 1,06427 |  |
| 227503_at | PVRL3 | Poliovirus receptor-related 3 | -0,555915 | -0,82813 | 1,73006 | 3,25444 |  |
| 242064_at | SDK2 | sidekick homolog 2 (chicken) | -1,456295 | 0,07401 | 1,72786 | 0,25444 |  |
| 212706_at | RASA4 | RAS p21 protein activator 4 | 0,1397042 | -1,52178 | 1,7276 | 0,43903 |  |
| 201513_at | TSN | translin | -0,798719 | -0,58272 | 1,7268 | 1,43745 |  |
| 224704_at | TNRC6A | trinucleotide repeat containing 6A | -0,632668 | -0,74762 | 1,72536 | 0,60662 |  |
| 209292_at | ID4 | Inhibitor of DNA binding 4, dominant negative helix-loop-helix protein | -0,655192 | -0,72312 | 1,72289 | 0,33143 |  |
| 202970_at | DYRK2 | MRNA; cDNA DKFZp667B0924 (from clone DKFZp667B0924) | -0,749901 | -0,62784 | 1,72218 | 1,43745 |  |
| 221841_s_at | KLF4 | Kruppel-like factor 4 (gut) | -0,55384 | -0,82363 | 1,72184 | 0,60662 |  |
| 201795_at | LBR | lamin B receptor | -0,481585 | -0,89481 | 1,72049 | 0,82259 |  |
| 229410_at | PAEP | Progestagen-associated endometrial protein (placental protein 14, pregnancy-associated endometrial alpha-2-globulin, alpha uterine protein) | -0,548294 | -0,82776 | 1,72006 | 0,60662 |  |
| 218319_at | PELI1 | pellino homolog 1 (Drosophila) | -0,468327 | -0,90765 | 1,71997 | 0,60662 |  |
| 228749_at | KIAA1571 | KIAA1571 protein | -0,480631 | -0,89391 | 1,71817 | 1,06427 |  |
| 235497_at | LOC643837 | hypothetical protein LOC643837 | -0,584776 | -0,78851 | 1,71661 | 3,25444 |  |
| 205668_at | LY75 | lymphocyte antigen 75 | -0,184015 | -1,18779 | 1,71475 | 0,60662 |  |
| 225048_at | PHF10 | PHD finger protein 10 | -0,193487 | -1,17511 | 1,71075 | 0,25444 |  |
| 91816_f_at | RKHD1 | ring finger and KH domain containing 1 | -0,694276 | -0,67354 | 1,70977 | 1,06427 |  |
| 230403_at | RFX3 | CDNA FLJ45905 fis, clone OCBBF3026576 | -0,384157 | -0,98205 | 1,70776 | 1,43745 |  |
| 230480_at | PIWIL4 | piwi-like 4 (Drosophila) | -0,281585 | -1,08304 | 1,70578 | 1,90223 |  |
| 224015_s_at | MRPS25 | mitochondrial ribosomal protein S25 | -0,269494 | -1,09406 | 1,70445 | 2,53333 |  |
| 1569459_a_at | ZNF451 | Zinc finger protein 451 | -0,403383 | -0,95934 | 1,70341 | 0,82259 |  |
| 235348_at | ABHD13 | abhydrolase domain containing 13 | -0,560039 | -0,80195 | 1,70249 | 3,25444 |  |
| 219888_at | SPAG4 | sperm associated antigen 4 | -0,13925 | -1,2218 | 1,70132 | 0,43903 |  |
| 221175_at | C3orf36 | chromosome 3 open reading frame 36 | -1,499769 | 0,14078 | 1,69874 | 0,60662 |  |
| 225330_at | IGF1R | insulin-like growth factor 1 receptor | -0,251869 | -1,10598 | 1,69732 | 0,25444 |  |
| 212824_at | FUBP3 | far upstream element (FUSE) binding protein 3 | -0,28957 | -1,06803 | 1,697 | 0,33143 |  |
| 203739_at | ZNF217 | zinc finger protein 217 | -0,71746 | -0,63708 | 1,69317 | 0,60662 |  |
| 243278_at | FOXP2 | Forkhead box P2 | -0,581439 | -0,77267 | 1,69263 | 3,25444 |  |
| 221217_s_at | A2BP1 | ataxin 2-binding protein 1 | -0,257864 | -1,09437 | 1,6903 | 0,82259 |  |
| 208415_x_at | ING1 | inhibitor of growth family, member 1 | -0,576511 | -0,77455 | 1,68883 | 0,60662 |  |
| 228167_at | KLHL6 | kelch-like 6 (Drosophila) | -0,845767 | -0,5019 | 1,68458 | 3,25444 |  |
| 230875_s_at | ATP11A | ATPase, Class VI, type 11A | -0,88376 | -0,46045 | 1,68026 | 0,60662 |  |
| 200685_at | SFRS11 | splicing factor, arginine/serine-rich 11 | -0,438067 | -0,90526 | 1,67916 | 1,06427 |  |
| 209681_at | SLC19A2 | solute carrier family 19 (thiamine transporter), member 2 | -0,446431 | -0,89541 | 1,6773 | 0,43903 |  |
| 241986_at | BMPER | BMP binding endothelial regulator | -0,951804 | -0,38984 | 1,67705 | 3,25444 |  |
| 1569030_s_at | NUB1 | negative regulator of ubiquitin-like proteins 1 | 0,8615297 | -2,203 | 1,67684 | 0 |  |
| 212931_at | TCF20 | transcription factor 20 (AR1) | -0,591125 | -0,74789 | 1,67377 | 1,43745 |  |
| 204679_at | KCNK1 | potassium channel, subfamily K, member 1 | -0,191833 | -1,14589 | 1,67216 | 0,25444 |  |
| 205174_s_at | QPCT | glutaminyl-peptide cyclotransferase (glutaminyl cyclase) | -0,468192 | -0,86944 | 1,67204 | 0,82259 |  |
| 203505_at | ABCA1 | ATP-binding cassette, sub-family A (ABC1), member 1 | -0,457714 | -0,87383 | 1,66443 | 0,43903 |  |
| 201631_s_at | IER3 | immediate early response 3 | -0,466457 | -0,86469 | 1,66393 | 0,43903 |  |
| 238205_at | WDR40B | WD repeat domain 40B | -0,654739 | -0,67595 | 1,66336 | 1,43745 |  |
| 229991_s_at | SYTL4 | Synaptotagmin-like 4 (granuphilin-a) | -0,7808 | -0,54976 | 1,6632 | 1,43745 |  |
| 203299_s_at | AP1S2 /// LOC653653 /// LOC654127 | adaptor-related protein complex 1, sigma 2 subunit /// similar to adaptor-related protein complex 1 sigma 2 subunit /// similar to adaptor-related protein complex 1 sigma 2 subunit | -0,881467 | -0,44898 | 1,66306 | 4,2202 |  |
| 203206_at | FAM53B | family with sequence similarity 53, member B | -0,556721 | -0,77185 | 1,66072 | 0,82259 |  |
| 219489_s_at | NXN | nucleoredoxin | -0,51232 | -0,81338 | 1,65712 | 1,43745 |  |
| 229831_at | CNTN3 | contactin 3 (plasmacytoma associated) | -1,203788 | -0,11682 | 1,65076 | 0,82259 |  |
| 204646_at | DPYD | dihydropyrimidine dehydrogenase | -0,165805 | -1,15354 | 1,64918 | 0,25444 |  |
| 218868_at | ACTR3B | ARP3 actin-related protein 3 homolog B (yeast) | -0,92883 | -0,39001 | 1,64855 | 4,2202 |  |
| 228519_x_at | CIRBP | cold inducible RNA binding protein | -0,316963 | -1,00028 | 1,64655 | 1,43745 |  |
| 223239_at | C14orf129 | chromosome 14 open reading frame 129 | -0,331385 | -0,98508 | 1,64558 | 0,60662 |  |
| 208682_s_at | MAGED2 | melanoma antigen family D, 2 | -0,96415 | -0,35153 | 1,6446 | 0,43903 |  |
| 226970_at | FBXO33 | F-box protein 33 | -0,181006 | -1,13418 | 1,64399 | 1,06427 |  |
| 228415_at | AP1S2 | Adaptor-related protein complex 1, sigma 2 subuni | -0,694558 | -0,61737 | 1,6399 | 1,06427 |  |
| 204619_s_at | CSPG2 | chondroitin sulfate proteoglycan 2 (versican) | -0,914863 | -0,39592 | 1,63848 | 0,43903 |  |
| 202364_at | MXI1 | MAX interactor 1 /// MAX interactor 1 | -0,616957 | -0,69032 | 1,6341 | 0,60662 |  |
| 213350_at | RPS11 | Ribosomal protein S11 | -0,688853 | -0,61791 | 1,63346 | 0,43903 |  |
| 232235_at | C18orf4 | chromosome 18 open reading frame 4 | -0,380093 | -0,92503 | 1,6314 | 0,82259 |  |
| 235603_at | HNRPU | heterogeneous nuclear ribonucleoprotein U (scaffold attachment factor A) | -0,565514 | -0,73611 | 1,62703 | 2,53333 |  |
| 1554241_at | COCH | coagulation factor C homolog, cochlin (Limulus polyphemus) | -0,99696 | -0,30349 | 1,62556 | 1,06427 |  |
| 235301_at | KIAA1324L | KIAA1324-like | -0,450237 | -0,84917 | 1,62426 | 0,82259 |  |
| 227812_at | TNFRSF19 | tumor necrosis factor receptor superfamily, member 19 | -1,152661 | -0,14632 | 1,62373 | 1,90223 |  |
| 229465_s_at | PTPRS | Protein tyrosine phosphatase, receptor type, S | -0,845489 | -0,44934 | 1,61854 | 1,43745 |  |
| 217039_x_at | IGHG1 | Immunoglobulin heavy constant gamma 1 (G1m marker) | -0,187544 | -1,10647 | 1,61751 | 0,82259 |  |
| 229018_at | C12orf26 | chromosome 12 open reading frame 26 | -0,060118 | -1,2322 | 1,61539 | 2,53333 |  |
| 226612_at | FLJ25076 | similar to CG4502-PA | -0,90311 | -0,38596 | 1,61134 | 0,82259 |  |
| 213894_at | THSD7A | thrombospondin, type I, domain containing 7A | -0,976295 | -0,31229 | 1,61074 | 2,53333 |  |
| 227153_at | IMMP2L | IMP2 inner mitochondrial membrane peptidase-like (S. cerevisiae) | 0,4452001 | -1,73319 | 1,60999 | 0,25444 |  |
| 223773_s_at | C1orf79 | chromosome 1 open reading frame 79 | -0,503572 | -0,78342 | 1,60874 | 1,90223 |  |
| 218414_s_at | NDE1 | nudE nuclear distribution gene E homolog 1 (A. nidulans) | 0,0991207 | -1,38603 | 1,60864 | 1,43745 |  |
| 211038_s_at | CROCC /// MGC12760 /// LOC729559 | ciliary rootlet coiled-coil, rootletin /// ciliary rootlet coiled-coil, rootletin /// hypothetical protein MGC12760 /// hypothetical protein MGC12760 /// similar to ciliary rootlet coiled-coil, rootletin /// similar to ciliary rootlet coiled-coil, rootlet | -0,345268 | -0,94102 | 1,60786 | 2,53333 |  |
| 225634_at | ZC3HAV1 | zinc finger CCCH-type, antiviral 1 | -0,1047 | -1,18104 | 1,60718 | 0,43903 |  |
| 238043_at | LOC729446 | similar to AT rich interactive domain 1B (SWI1-like) isoform 1 | -0,085306 | -1,19802 | 1,60415 | 1,90223 |  |
| 225144_at | BMPR2 | bone morphogenetic protein receptor, type II (serine/threonine kinase) | 0,0624789 | -1,34578 | 1,60412 | 0,16318 |  |
| 235007_at | BBS7 | Bardet-Biedl syndrome 7 | -0,539793 | -0,74129 | 1,60135 | 3,25444 |  |
| 205449_at | SAC3D1 | SAC3 domain containing 1 | -0,370373 | -0,91029 | 1,60083 | 1,90223 |  |
| 209054_s_at | WHSC1 | Wolf-Hirschhorn syndrome candidate 1 | -0,27486 | -1,00497 | 1,59979 | 2,53333 |  |
| 219631_at | LRP12 | low density lipoprotein-related protein 12 | -0,591025 | -0,6865 | 1,59691 | 1,06427 |  |
| 226247_at | PLEKHA1 | pleckstrin homology domain containing, family A (phosphoinositide binding specific) member 1 | -0,298271 | -0,97888 | 1,59643 | 1,90223 |  |
| 227337_at | ANKRD37 | ankyrin repeat domain 37 | -0,88551 | -0,38974 | 1,59407 | 1,90223 |  |
| 225971_at | DDHD1 | CDNA FLJ34209 fis, clone FCBBF3020599 | -0,258469 | -1,01587 | 1,59293 | 1,43745 |  |
| 214093_s_at | FUBP1 | far upstream element (FUSE) binding protein 1 | -0,625649 | -0,64826 | 1,59239 | 3,25444 |  |
| 212044_s_at | RPL27A | Ribosomal protein L27a | -0,734998 | -0,5375 | 1,59063 | 0,82259 |  |
| 227778_at | KIAA1833 /// LOC727957 | hypothetical protein KIAA1833 /// similar to c11.1 CG12132-PA | -0,789977 | -0,4803 | 1,58785 | 3,25444 |  |
| 226868_at | GLT8D3 | glycosyltransferase 8 domain containing 3 | -0,325692 | -0,93907 | 1,58095 | 0,82259 |  |
| 227188_at | C21orf63 | chromosome 21 open reading frame 63 | -1,347695 | 0,08307 | 1,58078 | 1,90223 |  |
| 205787_x_at | ZC3H11A | zinc finger CCCH-type containing 11A | -0,388512 | -0,87398 | 1,57811 | 1,43745 |  |
| 221527_s_at | PARD3 | par-3 partitioning defective 3 homolog (C. elegans) | -0,373606 | -0,88756 | 1,57646 | 3,25444 |  |
| 203743_s_at | TDG | thymine-DNA glycosylase | -0,142556 | -1,11844 | 1,57625 | 0,43903 |  |
| 229130_at | LOC285535 | hypothetical protein LOC285535 | -0,827359 | -0,43279 | 1,57518 | 4,2202 |  |
| 219353_at | NHLRC2 | NHL repeat containing 2 | -0,621553 | -0,63785 | 1,57425 | 1,90223 |  |
| 209686_at | S100B | S100 calcium binding protein B | -0,886952 | -0,37244 | 1,57423 | 2,53333 |  |
| 227002_at | FAM78A | family with sequence similarity 78, member A | -0,542912 | -0,71601 | 1,57366 | 4,2202 |  |
| 217753_s_at | RPS26 /// LOC644166 /// LOC644191 /// LOC728937 | ribosomal protein S26 /// similar to 40S ribosomal protein S26 /// similar to 40S ribosomal protein S26 /// similar to 40S ribosomal protein S26 | 0,3110174 | -1,5682 | 1,57148 | 0,06315 |  |
| 228005_at | ZXDB | zinc finger, X-linked, duplicated B | -0,304044 | -0,95142 | 1,56934 | 1,06427 |  |
| 216960_s_at | ZNF133 | zinc finger protein 133 | -0,141303 | -1,1136 | 1,56863 | 1,90223 |  |
| 202389_s_at | HD | huntingtin (Huntington disease) | -0,929495 | -0,32535 | 1,56856 | 2,53333 |  |
| 227693_at | WDR20 | WD repeat domain 20 | -0,61919 | -0,63347 | 1,56583 | 4,2202 |  |
| 219440_at | RAI2 | retinoic acid induced 2 | -0,832965 | -0,41913 | 1,56512 | 2,53333 |  |
| 211596_s_at | LRIG1 | leucine-rich repeats and immunoglobulin-like domains 1 /// leucine-rich repeats and immunoglobulin-like domains 1 | -0,558148 | -0,69324 | 1,56423 | 1,43745 |  |
| 219310_at | C20orf39 | chromosome 20 open reading frame 39 | -0,859293 | -0,3904 | 1,56212 | 2,53333 |  |
| 202097_at | NUP153 | nucleoporin 153kDa | -0,436741 | -0,81278 | 1,5619 | 1,06427 |  |
| 213979_s_at | CTBP1 | C-terminal binding protein 1 | -1,042726 | -0,20675 | 1,56184 | 1,06427 |  |
| 202613_at | CTPS | CTP synthase | -0,764993 | -0,48085 | 1,5573 | 1,43745 |  |
| 218566_s_at | CHORDC1 | cysteine and histidine-rich domain (CHORD)-containing 1 | -0,380086 | -0,86574 | 1,55729 | 1,06427 |  |
| 205874_at | ITPKA | inositol 1,4,5-trisphosphate 3-kinase A | -0,501402 | -0,74387 | 1,55659 | 2,53333 |  |
| 228711_at | ZNF37A | zinc finger protein 37A | -0,681463 | -0,5638 | 1,55657 | 1,43745 |  |
| 207414_s_at | PCSK6 | proprotein convertase subtilisin/kexin type 6 | -0,635591 | -0,60751 | 1,55388 | 0,82259 |  |
| 224739_at | PIM3 | pim-3 oncogene | -0,118588 | -1,12351 | 1,55262 | 0,43903 |  |
| 219582_at | OGFRL1 | opioid growth factor receptor-like 1 | 0,184587 | -1,4265 | 1,55239 | 1,43745 |  |
| 204512_at | HIVEP1 | human immunodeficiency virus type I enhancer binding protein 1 | -0,518084 | -0,72324 | 1,55166 | 2,53333 |  |
| 204776_at | THBS4 | thrombospondin 4 | -0,973111 | -0,26648 | 1,54949 | 0,60662 |  |
| 210778_s_at | MXD4 | MAX dimerization protein 4 | -1,171275 | -0,06179 | 1,54133 | 1,06427 |  |
| 203604_at | ZNF516 | zinc finger protein 516 | -0,432027 | -0,80088 | 1,54114 | 2,53333 |  |
| 225680_at | DKFZp434K1815 | hypothetical protein DKFZp434K1815 | -0,323524 | -0,90773 | 1,53907 | 4,2202 |  |
| 208383_s_at | PCK1 | phosphoenolpyruvate carboxykinase 1 (soluble) | -0,687739 | -0,54102 | 1,53595 | 1,43745 |  |
| 210145_at | PLA2G4A | phospholipase A2, group IVA (cytosolic, calcium-dependent) | -0,722565 | -0,50495 | 1,53439 | 1,06427 |  |
| 244163_at | SEMA3A | sema domain, immunoglobulin domain (Ig), short basic domain, secreted, (semaphorin) 3A | -0,635411 | -0,5917 | 1,53389 | 1,06427 |  |
| 235501_at | FARP1 | FERM, RhoGEF (ARHGEF) and pleckstrin domain protein 1 (chondrocyte-derived) | -0,42774 | -0,79698 | 1,5309 | 1,90223 |  |
| 203098_at | CDYL | chromodomain protein, Y-like | -0,294503 | -0,92947 | 1,52996 | 1,90223 |  |
| 222565_s_at | PRKD3 | protein kinase D3 | -0,563032 | -0,65529 | 1,5229 | 2,53333 |  |
| 220370_s_at | USP36 | ubiquitin specific peptidase 36 | 0,004277 | -1,22209 | 1,52227 | 4,2202 |  |
| 204976_s_at | AMMECR1 | Alport syndrome, mental retardation, midface hypoplasia and elliptocytosis chromosomal region, gene 1 | -0,052761 | -1,16298 | 1,51967 | 0,43903 |  |
| 220178_at | C19orf28 | chromosome 19 open reading frame 28 | -0,824113 | -0,39156 | 1,51959 | 3,25444 |  |
| 218696_at | EIF2AK3 | eukaryotic translation initiation factor 2-alpha kinase 3 | -0,187645 | -1,02697 | 1,51827 | 0,82259 |  |
| 227488_at | MGC16121 | hypothetical protein MGC16121 | -0,173211 | -1,03979 | 1,51625 | 1,06427 |  |
| 204900_x_at | SAP30 | Sin3A-associated protein, 30kDa | -0,602141 | -0,60628 | 1,51053 | 3,25444 |  |
| 214787_at | DENND4A | DENN/MADD domain containing 4A | -0,348863 | -0,853 | 1,50233 | 3,25444 |  |
| 209272_at | NAB1 | NGFI-A binding protein 1 (EGR1 binding protein 1) | -0,330427 | -0,87096 | 1,50173 | 1,43745 |  |
| 32062_at | LRRC14 | leucine rich repeat containing 14 | -0,636498 | -0,56305 | 1,49943 | 4,2202 |  |
| 218624_s_at | MGC2752 | hypothetical protein MGC2752 | 0,0225741 | -1,22164 | 1,49883 | 1,43745 |  |
| 218487_at | ALAD | aminolevulinate, delta-, dehydratase | -0,321255 | -0,87713 | 1,49798 | 0,82259 |  |
| 225711_at | ARL6IP6 | ADP-ribosylation-like factor 6 interacting protein 6 | -0,597158 | -0,60119 | 1,49793 | 4,2202 |  |
| 225398_at | RPUSD4 | RNA pseudouridylate synthase domain containing 4 | -0,322112 | -0,87587 | 1,49748 | 2,53333 |  |
| 205251_at | PER2 | period homolog 2 (Drosophila) | -0,277902 | -0,91699 | 1,49361 | 2,53333 |  |
| 1558102_at | TM6SF1 | Transmembrane 6 superfamily member 1 | -1,111996 | -0,08243 | 1,49303 | 2,53333 |  |
| 214714_at | ZNF394 | zinc finger protein 394 | -0,531626 | -0,6607 | 1,49041 | 3,25444 |  |
| 213918_s_at | NIPBL | Nipped-B homolog (Drosophila) | -0,068683 | -1,12359 | 1,49035 | 1,06427 |  |
| 226917_s_at | ANAPC4 | anaphase promoting complex subunit 4 | -0,243708 | -0,94395 | 1,48457 | 1,43745 |  |
| 205542_at | STEAP1 | six transmembrane epithelial antigen of the prostate 1 | -0,489305 | -0,69437 | 1,47959 | 1,43745 |  |
| 225325_at | FLJ20160 | FLJ20160 protein | -0,870548 | -0,31197 | 1,47814 | 1,06427 |  |
| 226872_at | RFX2 | regulatory factor X, 2 (influences HLA class II expression) | -0,109734 | -1,07229 | 1,47753 | 3,25444 |  |
| 222106_at | PRND | prion protein 2 (dublet) | -0,547217 | -0,63437 | 1,47698 | 1,43745 |  |
| 231775_at | TNFRSF10A | tumor necrosis factor receptor superfamily, member 10a | -0,327839 | -0,85372 | 1,47694 | 4,2202 |  |
| 230634_x_at | LOC113179 | hypothetical protein BC011824 | -1,506772 | 0,32695 | 1,47478 | 1,90223 |  |
| 202768_at | FOSB | FBJ murine osteosarcoma viral oncogene homolog B | -0,580458 | -0,59778 | 1,47279 | 1,43745 |  |
| 205945_at | IL6R | interleukin 6 receptor /// interleukin 6 receptor | -0,003248 | -1,17425 | 1,47187 | 0,82259 |  |
| 213010_at | PRKCDBP | protein kinase C, delta binding protein | 0,0554665 | -1,23057 | 1,46888 | 0,60662 |  |
| 200632_s_at | NDRG1 | N-myc downstream regulated gene 1 | -0,636443 | -0,53572 | 1,4652 | 1,43745 |  |
| 226200_at | VARSL | valyl-tRNA synthetase like | -0,80472 | -0,36447 | 1,46149 | 3,25444 |  |
| 225658_at | LOC339745 | hypothetical protein LOC339745 | -0,339286 | -0,82918 | 1,46058 | 2,53333 |  |
| 227351_at | C16orf52 | chromosome 16 open reading frame 52 | -0,238471 | -0,92927 | 1,45967 | 1,90223 |  |
| 238965_at | C21orf2 | Chromosome 21 open reading frame 2 | 0,2687162 | -1,43605 | 1,45917 | 1,90223 |  |
| 218330_s_at | NAV2 | neuron navigator 2 | -0,377373 | -0,7885 | 1,45734 | 2,53333 |  |
| 225318_at | DDHD2 | DDHD domain containing 2 | -0,47483 | -0,68855 | 1,45423 | 3,25444 |  |
| 230746_s_at | STC1 | Stanniocalcin 1 | -0,483486 | -0,67817 | 1,45207 | 1,90223 |  |
| 204916_at | RAMP1 | receptor (G protein-coupled) activity modifying protein 1 | -0,448518 | -0,71166 | 1,45022 | 1,90223 |  |
| 209967_s_at | CREM | cAMP responsive element modulator | -0,219011 | -0,93943 | 1,44805 | 2,53333 |  |
| 236046_at | FLJ44896 | FLJ44896 protein | 0,0543918 | -1,20848 | 1,44261 | 3,25444 |  |
| 207768_at | EGR4 | early growth response 4 | -0,368342 | -0,78392 | 1,44033 | 3,25444 |  |
| 218352_at | RCBTB1 | regulator of chromosome condensation (RCC1) and BTB (POZ) domain containing protein 1 | -0,490983 | -0,65484 | 1,43228 | 2,53333 |  |
| 212418_at | ELF1 | E74-like factor 1 (ets domain transcription factor) | -0,273632 | -0,87188 | 1,43189 | 1,43745 |  |
| 203475_at | CYP19A1 | cytochrome P450, family 19, subfamily A, polypeptide 1 | -0,653661 | -0,48565 | 1,42414 | 1,43745 |  |
| 217979_at | TSPAN13 | Tetraspanin 13 | -0,432539 | -0,70213 | 1,41833 | 3,25444 |  |
| 242263_at | TMED5 | transmembrane emp24 protein transport domain containing 5 | 0,0212979 | -1,14773 | 1,40804 | 1,06427 |  |
| 228204_at | PSMB4 | Proteasome (prosome, macropain) subunit, beta type, 4 | -0,683383 | -0,43772 | 1,40138 | 3,25444 |  |
| 203343_at | UGDH | UDP-glucose dehydrogenase | -0,012836 | -1,1076 | 1,40054 | 0,82259 |  |
| 202157_s_at | CUGBP2 | CUG triplet repeat, RNA binding protein 2 | -0,590709 | -0,52913 | 1,39979 | 2,53333 |  |
| 204464_s_at | EDNRA | endothelin receptor type A | -0,10591 | -1,01182 | 1,39716 | 1,43745 |  |
| 221763_at | JMJD1C | jumonji domain containing 1C | -0,474128 | -0,64269 | 1,39603 | 3,25444 |  |
| 224057_s_at | THAP4 /// LOC728944 | THAP domain containing 4 /// similar to THAP domain-containing protein 4 | -0,311821 | -0,80493 | 1,39594 | 2,53333 |  |
| 212522_at | PDE8A | phosphodiesterase 8A | -0,014451 | -1,0983 | 1,39094 | 1,06427 |  |
| 204347_at | LOC645619 /// LOC731007 | similar to Adenylate kinase isoenzyme 4, mitochondrial (ATP-AMP transphosphorylase) /// similar to Adenylate kinase isoenzyme 4, mitochondrial (Adenylate kinase 3-like 1) (ATP-AMP transphosphorylase) | -0,295306 | -0,81654 | 1,38981 | 2,53333 |  |
| 212875_s_at | C21orf25 | chromosome 21 open reading frame 25 | -0,196233 | -0,91314 | 1,38671 | 1,43745 |  |
| 231040_at | RORB | RAR-related orphan receptor B | -0,718166 | -0,38963 | 1,38474 | 2,53333 |  |
| 213194_at | ROBO1 | roundabout, axon guidance receptor, homolog 1 (Drosophila) | -0,397248 | -0,71053 | 1,38472 | 2,53333 |  |
| 205493_s_at | DPYSL4 | dihydropyrimidinase-like 4 | -0,472009 | -0,63466 | 1,38333 | 2,53333 |  |
| 222999_s_at | CCNL2 /// LOC727877 | cyclin L2 /// similar to Cyclin-L2 (Paneth cell-enhanced expression protein) | -0,120375 | -0,98485 | 1,38153 | 1,43745 |  |
| 201952_at | ALCAM | activated leukocyte cell adhesion molecule | -0,713598 | -0,39085 | 1,38056 | 2,53333 |  |
| 202668_at | EFNB2 | ephrin-B2 | -0,311217 | -0,79232 | 1,37942 | 1,90223 |  |
| 206036_s_at | REL | v-rel reticuloendotheliosis viral oncogene homolog (avian) | -0,077972 | -1,02534 | 1,37914 | 1,90223 |  |
| 208691_at | TFRC | transferrin receptor (p90, CD71) /// transferrin receptor (p90, CD71) | -0,169262 | -0,9307 | 1,37495 | 1,43745 |  |
| 202908_at | WFS1 | Wolfram syndrome 1 (wolframin) | -1,197874 | 0,09884 | 1,37379 | 1,90223 |  |
| 227140_at | Activin beta A | CDNA FLJ11041 fis, clone PLACE1004405 | -0,178466 | -0,91334 | 1,36476 | 1,43745 |  |
| 214016_s_at | SFPQ | splicing factor proline/glutamine-rich (polypyrimidine tract binding protein associated) | -0,200742 | -0,8898 | 1,36318 | 2,53333 |  |
| 207480_s_at | MEIS2 | Meis1, myeloid ecotropic viral integration site 1 homolog 2 (mouse) | -0,105232 | -0,98462 | 1,36232 | 1,43745 |  |
| 201673_s_at | GYS1 | glycogen synthase 1 (muscle) | -0,206675 | -0,88263 | 1,36163 | 1,90223 |  |
| 221760_at | MAN1A1 | Mannosidase, alpha, class 1A, member 1 | -0,250386 | -0,83754 | 1,35991 | 1,90223 |  |
| 212067_s_at | C1R | complement component 1, r subcomponent | -0,22243 | -0,86502 | 1,35931 | 1,90223 |  |
| 227081_at | DNALI1 | dynein, axonemal, light intermediate chain 1 | -0,168917 | -0,91796 | 1,3586 | 3,25444 |  |
| 235589_s_at | MDM4 | Mdm4, transformed 3T3 cell double minute 4, p53 binding protein (mouse) | -0,89958 | -0,18619 | 1,35721 | 3,25444 |  |
| 209277_at | TFPI2 | tissue factor pathway inhibitor 2 | -0,222028 | -0,86082 | 1,35357 | 1,90223 |  |
| 226333_at | IL6RA | Interleukin 6 receptor | 0,2461741 | -1,32874 | 1,35321 | 0,60662 |  |
| 207057_at | SLC16A7 | solute carrier family 16, member 7 (monocarboxylic acid transporter 2) | -0,86159 | -0,21955 | 1,35142 | 3,25444 |  |
| 226326_at | PCGF5 | polycomb group ring finger 5 | -0,542289 | -0,53862 | 1,35114 | 3,25444 |  |
| 201505_at | LAMB1 | laminin, beta 1 | -1,094943 | 0,01439 | 1,35069 | 1,06427 |  |
| 225355_at | DKFZP761M1511 | hypothetical protein DKFZP761M1511 | -0,9433 | -0,1328 | 1,34512 | 1,90223 |  |
| 213225_at | PPM1B | protein phosphatase 1B (formerly 2C), magnesium-dependent, beta isoform | -0,991298 | -0,08374 | 1,3438 | 1,90223 |  |
| 230372_at | HAS2 | Hyaluronan synthase 2 | -0,736829 | -0,33709 | 1,34239 | 2,53333 |  |
| 218055_s_at | WDR41 | WD repeat domain 41 | 0,0260326 | -1,08351 | 1,32184 | 2,53333 |  |
| 218816_at | LRRC1 | leucine rich repeat containing 1 | -0,151548 | -0,90179 | 1,31668 | 4,2202 |  |
| 225885_at | EEA1 | early endosome antigen 1, 162kD | -0,317051 | -0,73222 | 1,31159 | 4,2202 |  |
| 212054_x_at | TBC1D9B | TBC1 domain family, member 9B (with GRAM domain) | -0,821239 | -0,22622 | 1,30932 | 4,2202 |  |
| 226281_at | DNER | delta/notch-like EGF repeat containing | -0,922487 | -0,11809 | 1,30072 | 3,25444 |  |
| 200953_s_at | CCND2 | cyclin D2 | -0,714507 | -0,32168 | 1,29524 | 2,53333 |  |
| 221798_x_at | RPS2 | Ribosomal protein S2 | -0,546633 | -0,48381 | 1,28805 | 3,25444 |  |
| 222808_at | ALG13 | asparagine-linked glycosylation 13 homolog (S. cerevisiae) | -0,152989 | -0,87558 | 1,28571 | 3,25444 |  |
| 229331_at | SPATA18 | spermatogenesis associated 18 homolog (rat) | 0,2571075 | -1,2807 | 1,27949 | 4,2202 |  |
| 212097_at | CAV1 | caveolin 1, caveolae protein, 22kDa | -0,429749 | -0,59224 | 1,27749 | 4,2202 |  |
| 204416_x_at | APOC1 | apolipoprotein C-I | -0,854931 | -0,15901 | 1,26742 | 2,53333 |  |
| 204487_s_at | KCNQ1 | potassium voltage-gated channel, KQT-like subfamily, member 1 | 0,1528079 | -1,16663 | 1,26728 | 3,25444 |  |
| 36711_at | MAFF | v-maf musculoaponeurotic fibrosarcoma oncogene homolog F (avian) | -0,199942 | -0,80857 | 1,26065 | 2,53333 |  |
| 209505_at | NR2F1 | Nuclear receptor subfamily 2, group F, member 1 | -0,094606 | -0,91375 | 1,26045 | 2,53333 |  |
| 229957_at | TMEM91 | transmembrane protein 91 | 0,029905 | -1,03586 | 1,25745 | 4,2202 |  |
| 201492_s_at | RPL41 | ribosomal protein L41 | -0,46544 | -0,53459 | 1,25003 | 4,2202 |  |
| 203180_at | ALDH1A3 | aldehyde dehydrogenase 1 family, member A3 | -0,084503 | -0,90455 | 1,23632 | 4,2202 |  |
| 212843_at | NCAM1 | neural cell adhesion molecule 1 | -1,266735 | 0,28291 | 1,22979 | 0,82259 |  |
| 1555653_at | HNRPA3 | heterogeneous nuclear ribonucleoprotein A3 | -0,865066 | -0,10686 | 1,21491 | 2,53333 |  |
| 203837_at | MAP3K5 /// LOC729144 /// LOC732274 | mitogen-activated protein kinase kinase kinase 5 /// hypothetical protein LOC729144 /// hypothetical protein LOC732274 | -0,00921 | -0,95855 | 1,2097 | 2,53333 |  |
| 239161_at | FDX1 | ferredoxin 1 | 0,0830529 | -1,04877 | 1,20715 | 1,90223 |  |
| 204715_at | PANX1 | pannexin 1 | -0,110466 | -0,84568 | 1,19518 | 4,2202 |  |
| 228483_s_at | TAF9B | TAF9B RNA polymerase II, TATA box binding protein (TBP)-associated factor, 31kDa | 0,0721051 | -1,01418 | 1,17759 | 3,25444 |  |
| 226568_at | FAM102B | family with sequence similarity 102, member B | 0,3198123 | -1,25874 | 1,17365 | 0,82259 |  |
| 216028_at | DKFZP564C152 | DKFZP564C152 protein | 0,332737 | -1,25666 | 1,1549 | 1,43745 |  |
| 230183_at | Exostosin-1 |  | 0,038318 | -0,95294 | 1,14328 | 4,2202 |  |
| 206115_at | EGR3 | early growth response 3 | 0,057021 | -0,96492 | 1,13488 | 3,25444 |  |
| 226683_at | SNAG1 | Sorting nexin associated golgi protein 1 | -1,01498 | 0,12741 | 1,10946 | 2,53333 |  |
| 230376_at | GCNT3 | Glucosaminyl (N-acetyl) transferase 3, mucin type | -1,517232 | 0,67442 | 1,05351 | 3,25444 |  |
| 227493_s_at | KIAA1143 | KIAA1143 | 0,3376282 | -1,17227 | 1,04331 | 2,53333 |  |
| 208891_at | DUSP6 | dual specificity phosphatase 6 | 0,3589582 | -1,13772 | 0,97345 | 2,53333 |  |
| 213457_at | MFHAS1 | malignant fibrous histiocytoma amplified sequence 1 | 0,7109341 | -1,44182 | 0,9136 | 0,60662 |  |
| 222796_at | PTCD1 | pentatricopeptide repeat domain 1 | -1,661622 | 0,98692 | 0,84337 | 1,43745 |  |
| 213763_at | HIPK2 | Homeodomain interacting protein kinase 2 | -1,283539 | 0,96431 | 0,39904 | 3,25444 |  |
| 1569191_at | FLJ44894 | similar to zinc finger protein 91 | -1,319494 | 1,15501 | 0,2056 | 4,2202 |  |
| 218309_at | CAMK2N1 | calcium/calmodulin-dependent protein kinase II inhibitor 1 | -1,42067 | 1,50497 | -0,10538 | 0,16318 |  |
| 227209_at | CNTN1 | Contactin 1 | 1,3584327 | -0,99164 | -0,45849 | 1,43745 |  |
| 203632_s_at | GPRC5B | G protein-coupled receptor, family C, group 5, member B | -1,077692 | 1,52266 | -0,5562 | 0,60662 |  |
| 204065_at | CHST10 | carbohydrate sulfotransferase 10 | -1,325577 | 1,87789 | -0,69039 | 0,33143 |  |
| 202450_s_at | CTSK | cathepsin K | 1,1913286 | -0,60144 | -0,73736 | 1,90223 |  |
| 226438_at | MTBP | Mdm2, transformed 3T3 cell double minute 2, p53 binding protein (mouse) binding protein, 104kDa | -1,040472 | 1,66408 | -0,77951 | 0,33143 |  |
| 203139_at | DAPK1 | death-associated protein kinase 1 | -0,668773 | 1,30342 | -0,7933 | 1,90223 |  |
| 225701_at | AKNA | AT-hook transcription factor | -0,763091 | 1,40233 | -0,79904 | 1,43745 |  |
| 221933_at | NLGN4X | neuroligin 4, X-linked | -0,653395 | 1,30314 | -0,81218 | 1,90223 |  |
| 223434_at | GBP3 | guanylate binding protein 3 | 1,1918073 | -0,53649 | -0,81915 | 3,25444 |  |
| 205139_s_at | UST | uronyl-2-sulfotransferase | -1,020346 | 1,68464 | -0,83036 | 0,16318 |  |
| 205967_at | HIST1H4C | histone cluster 1, H4c | -0,52522 | 1,22063 | -0,86926 | 1,90223 |  |
| 227590_at | LOC150383 | similar to RIKEN cDNA 2210021J22 | -0,875102 | 1,61175 | -0,92081 | 3,25444 |  |
| 225169_at | INTS4 | integrator complex subunit 4 | -0,984604 | 1,73664 | -0,94005 | 0,82259 |  |
| 210910_s_at | POMZP3 | POM (POM121 homolog, rat) and ZP3 fusion | 1,2583806 | -0,50508 | -0,94162 | 3,25444 |  |
| 227152_at | C12orf35 | chromosome 12 open reading frame 35 | -0,430174 | 1,19266 | -0,9531 | 2,53333 |  |
| 238650_x_at | WDR89 | WD repeat domain 89 | -0,644164 | 1,42669 | -0,97816 | 2,53333 |  |
| 203395_s_at | HES1 | hairy and enhancer of split 1, (Drosophila) | 1,7943813 | -1,00321 | -0,98897 | 0,60662 |  |
| 204803_s_at | RRAD | Ras-related associated with diabetes | -0,309087 | 1,10313 | -0,99255 | 4,2202 |  |
| 224735_at | CYBASC3 | cytochrome b, ascorbate dependent 3 | -0,530585 | 1,33626 | -1,00709 | 1,90223 |  |
| 204148_s_at | ZP3 | zona pellucida glycoprotein 3 (sperm receptor) /// POM (POM121 homolog, rat) and ZP3 fusion /// Meis1, myeloid ecotropic viral integration site 1 homolog 3 (mouse) | 1,6234433 | -0,80305 | -1,02549 | 0,43903 |  |
| 213248_at | LOC221362 /// LOC730101 | hypothetical protein LOC221362 /// similar to heterogeneous nuclear ribonucleoprotein A/B | -0,527458 | 1,36988 | -1,05303 | 1,06427 |  |
| 203346_s_at | MTF2 | metal response element binding transcription factor 2 | 1,1472619 | -0,29805 | -1,06152 | 4,2202 |  |
| 219774_at | CCDC93 | coiled-coil domain containing 93 | -0,47126 | 1,33949 | -1,08528 | 3,25444 |  |
| 160020_at | MMP14 | matrix metallopeptidase 14 (membrane-inserted) | -0,194094 | 1,08002 | -1,10741 | 3,25444 |  |
| 1554547_at | FAM13C1 | family with sequence similarity 13, member C1 | -0,367782 | 1,25408 | -1,10787 | 3,25444 |  |
| 218929_at | CDKN2AIP | CDKN2A interacting protein | -0,151135 | 1,04755 | -1,12052 | 4,2202 |  |
| 204143_s_at | ENOSF1 | enolase superfamily member 1 | 1,5376187 | -0,63701 | -1,12576 | 0,60662 |  |
| 206286_s_at | TDGF1 /// TDGF3 | teratocarcinoma-derived growth factor 1 /// teratocarcinoma-derived growth factor 3, pseudogene | -0,438438 | 1,3487 | -1,13783 | 2,53333 |  |
| 206516_at | AMH | anti-Mullerian hormone | 1,3317298 | -0,42064 | -1,13887 | 0,82259 |  |
| 204174_at | ALOX5AP | arachidonate 5-lipoxygenase-activating protein | -0,131027 | 1,0487 | -1,14709 | 3,25444 |  |
| 206993_at | ATP5S | ATP synthase, H+ transporting, mitochondrial F0 complex, subunit s (factor B) | -0,381416 | 1,30575 | -1,15542 | 3,25444 |  |
| 217117_x_at | MUC3A | mucin 3A, cell surface associated | -0,823519 | 1,7531 | -1,16198 | 1,43745 |  |
| 228245_s_at | HELZ /// OVOS2 /// LOC728715 | helicase with zinc finger /// ovostatin 2 /// similar to cDNA sequence BC048546 | -0,34671 | 1,27765 | -1,16367 | 1,06427 |  |
| 44783_s_at | HEY1 | hairy/enhancer-of-split related with YRPW motif 1 | -0,719991 | 1,66084 | -1,17606 | 2,53333 |  |
| 200974_at | ACTA2 | actin, alpha 2, smooth muscle, aorta | -0,165659 | 1,10751 | -1,17731 | 1,43745 |  |
| 220947_s_at | TBC1D10B | TBC1 domain family, member 10B | -0,063063 | 1,01381 | -1,18843 | 3,25444 |  |
| 219414_at | CLSTN2 | calsyntenin 2 | -0,130497 | 1,0813 | -1,1885 | 2,53333 |  |
| 201325_s_at | EMP1 | epithelial membrane protein 1 | 1,0103491 | -0,04371 | -1,20829 | 3,25444 |  |
| 213479_at | NPTX2 | neuronal pentraxin II | 0,7577952 | 0,217 | -1,2185 | 3,25444 |  |
| 219997_s_at | COPS7B | COP9 constitutive photomorphogenic homolog subunit 7B (Arabidopsis) | -0,268858 | 1,24547 | -1,22077 | 3,25444 |  |
| 219066_at | PPCDC | phosphopantothenoylcysteine decarboxylase | -0,666604 | 1,64371 | -1,22138 | 2,53333 |  |
| 209652_s_at | PGF | placental growth factor, vascular endothelial growth factor-related protein | -0,496266 | 1,47485 | -1,22323 | 1,90223 |  |
| 1554414_a_at | OSGIN2 | oxidative stress induced growth inhibitor family member 2 | -0,712322 | 1,69276 | -1,22555 | 0,60662 |  |
| 1555167_s_at | PBEF1 | pre-B-cell colony enhancing factor 1 | 1,1159135 | -0,13425 | -1,22708 | 2,53333 |  |
| 206108_s_at | SFRS6 | splicing factor, arginine/serine-rich 6 | 1,2042974 | -0,2204 | -1,22988 | 1,90223 |  |
| 231727_s_at | MIF4GD | MIF4G domain containing | -0,159403 | 1,15148 | -1,24009 | 3,25444 |  |
| 241765_at | CPM | carboxypeptidase M | 1,0815344 | -0,0856 | -1,24492 | 1,90223 |  |
| 221528_s_at | ELMO2 | engulfment and cell motility 2 | -0,359655 | 1,35753 | -1,24734 | 2,53333 |  |
| 226803_at | CHMP4C | chromatin modifying protein 4C | -0,24353 | 1,24258 | -1,24882 | 3,25444 |  |
| 233571_x_at | C20orf149 | chromosome 20 open reading frame 149 | 1,0938848 | -0,09124 | -1,25331 | 1,43745 |  |
| 235054_at | NUDT16 | nudix (nucleoside diphosphate linked moiety X)-type motif 16 | -0,290998 | 1,31255 | -1,27694 | 0,82259 |  |
| 223690_at | LTBP2 | latent transforming growth factor beta binding protein 2 | -0,401775 | 1,42754 | -1,28221 | 3,25444 |  |
| 1557905_s_at | CD44 /// MAPK10 | CD44 molecule (Indian blood group) /// mitogen-activated protein kinase 10 | 0,8683934 | 0,16289 | -1,2891 | 4,2202 |  |
| 203585_at | ZNF185 | zinc finger protein 185 (LIM domain) | -0,221868 | 1,2548 | -1,29117 | 1,06427 |  |
| 1554443_s_at | BEST1 | bestrophin 1 | -0,108147 | 1,14112 | -1,29121 | 3,25444 |  |
| 1553764_a_at | JUB | jub, ajuba homolog (Xenopus laevis) | -0,293107 | 1,32678 | -1,29209 | 1,90223 |  |
| 220346_at | MTHFD2L | methylenetetrahydrofolate dehydrogenase (NADP+ dependent) 2-like | 0,3339884 | 0,7061 | -1,30012 | 4,2202 |  |
| 227223_at | RBM39 /// LOC643167 | RNA binding motif protein 39 /// similar to RNA-binding region-containing protein 2 (Hepatocellular carcinoma protein 1) (Splicing factor HCC1) | 1,1985175 | -0,15706 | -1,30182 | 1,90223 |  |
| 1565681_s_at | DIP2C | DIP2 disco-interacting protein 2 homolog C (Drosophila) | -0,087764 | 1,13477 | -1,30875 | 3,25444 |  |
| 244071_at | LOC345630 | similar to fibrillarin | -0,309512 | 1,35947 | -1,31244 | 3,25444 |  |
| 204195_s_at | PKNOX1 | PBX/knotted 1 homeobox 1 | -0,603513 | 1,65474 | -1,31403 | 1,06427 |  |
| 218986_s_at | FLJ20035 | hypothetical protein FLJ20035 | 0,0821669 | 0,9776 | -1,32471 | 3,25444 |  |
| 233757_x_at | C1orf181 | Chromosome 1 open reading frame 181 | 0,9405726 | 0,12065 | -1,32653 | 1,90223 |  |
| 224798_s_at | C15orf17 | chromosome 15 open reading frame 17 | 1,6784055 | -0,61605 | -1,32795 | 1,06427 |  |
| 209305_s_at | GADD45B | growth arrest and DNA-damage-inducible, beta | 0,7016433 | 0,36849 | -1,33767 | 4,2202 |  |
| 218400_at | OAS3 | 2'-5'-oligoadenylate synthetase 3, 100kDa | 1,2705789 | -0,19444 | -1,34517 | 1,43745 |  |
| 211352_s_at | NCOA3 | nuclear receptor coactivator 3 | 1,3745129 | -0,29528 | -1,34904 | 3,25444 |  |
| 226198_at | TOM1L2 | target of myb1-like 2 (chicken) | 0,029229 | 1,05666 | -1,35736 | 3,25444 |  |
| 212280_x_at | LOC727737 | similar to APG4 autophagy 4 homolog B isoform a | 1,1214751 | -0,03407 | -1,35925 | 3,25444 |  |
| 240312_at | LOC389895 | similar to CG4768-PA | -0,018427 | 1,1072 | -1,36097 | 1,43745 |  |
| 208965_s_at | IFI16 | interferon, gamma-inducible protein 16 | -0,209285 | 1,30784 | -1,3732 | 0,43903 |  |
| 227424_x_at | C21orf119 | chromosome 21 open reading frame 119 | -0,714539 | 1,81328 | -1,37342 | 0,60662 |  |
| 218688_at | DAK | dihydroxyacetone kinase 2 homolog (S. cerevisiae) | -0,292378 | 1,39521 | -1,37854 | 3,25444 |  |
| 200956_s_at | SSRP1 | structure specific recognition protein 1 | -0,09028 | 1,19781 | -1,38441 | 1,43745 |  |
| 221887_s_at | DFNB31 | deafness, autosomal recessive 31 | 1,0182385 | 0,09435 | -1,39073 | 3,25444 |  |
| 226734_at | EIF4E2 | Eukaryotic translation initiation factor 4E family member 2 | -0,033277 | 1,14599 | -1,39089 | 3,25444 |  |
| 230109_at | PDE7B | phosphodiesterase 7B | 0,4263312 | 0,68689 | -1,39153 | 2,53333 |  |
| 223355_at | ALG1 | asparagine-linked glycosylation 1 homolog (S. cerevisiae, beta-1,4-mannosyltransferase) | 1,5139005 | -0,39224 | -1,40207 | 3,25444 |  |
| 221958_s_at | GPR177 | G protein-coupled receptor 177 | 0,0725542 | 1,04925 | -1,40225 | 1,43745 |  |
| 226745_at | CYP4V2 | cytochrome P450, family 4, subfamily V, polypeptide 2 | -0,706448 | 1,83255 | -1,40763 | 0,60662 |  |
| 235499_at | REPIN1 | Replication initiator 1 | -0,696679 | 1,82342 | -1,40843 | 1,43745 |  |
| 205805_s_at | ROR1 | receptor tyrosine kinase-like orphan receptor 1 | 0,063949 | 1,06309 | -1,40879 | 3,25444 |  |
| 218307_at | RSAD1 | radical S-adenosyl methionine domain containing 1 | -0,131304 | 1,25961 | -1,41038 | 3,25444 |  |
| 205606_at | LRP6 | low density lipoprotein receptor-related protein 6 | 1,3907028 | -0,26183 | -1,41109 | 1,90223 |  |
| 204643_s_at | COVA1 | cytosolic ovarian carcinoma antigen 1 | -0,23373 | 1,36806 | -1,41791 | 1,06427 |  |
| 221898_at | PDPN | podoplanin | 0,959882 | 0,17583 | -1,41964 | 4,2202 |  |
| 203088_at | FBLN5 | fibulin 5 | 0,5533676 | 0,58483 | -1,42274 | 3,25444 |  |
| 220317_at | LRAT | lecithin retinol acyltransferase (phosphatidylcholine--retinol O-acyltransferase) | 0,2875498 | 0,85142 | -1,42371 | 1,90223 |  |
| 235089_at | FBXL20 | F-box and leucine-rich repeat protein 20 | 0,1546921 | 0,98579 | -1,4256 | 2,53333 |  |
| 204363_at | F3 | coagulation factor III (thromboplastin, tissue factor) | 0,0816922 | 1,06071 | -1,428 | 0,82259 |  |
| 204945_at | PTPRN | protein tyrosine phosphatase, receptor type, N | -0,224151 | 1,36903 | -1,4311 | 1,06427 |  |
| 205032_at | ITGA2 | integrin, alpha 2 (CD49B, alpha 2 subunit of VLA-2 receptor) | 0,8691675 | 0,27703 | -1,43274 | 1,43745 |  |
| 205266_at | LIF | leukemia inhibitory factor (cholinergic differentiation factor) | 0,695345 | 0,4518 | -1,43393 | 3,25444 |  |
| 64883_at | MOSPD2 | motile sperm domain containing 2 | 0,3649051 | 0,78304 | -1,43493 | 4,2202 |  |
| 209087_x_at | MCAM | melanoma cell adhesion molecule | -0,135284 | 1,28499 | -1,43714 | 0,43903 |  |
| 210376_x_at | ELK1 | ELK1, member of ETS oncogene family | 0,3656646 | 0,78462 | -1,43785 | 2,53333 |  |
| 206618_at | IL18R1 | interleukin 18 receptor 1 | 1,408572 | -0,25791 | -1,43832 | 1,90223 |  |
| 227006_at | PPP1R14A | protein phosphatase 1, regulatory (inhibitor) subunit 14A | 1,077724 | 0,07422 | -1,43993 | 1,06427 |  |
| 216733_s_at | GATM | glycine amidinotransferase (L-arginine:glycine amidinotransferase) | 0,3722768 | 0,78317 | -1,4443 | 1,90223 |  |
| 229119_s_at | ACTB /// SWS1 | actin, beta /// SWIM-domain containing Srs2 interacting protein 1 | 0,2372132 | 0,91857 | -1,44472 | 1,90223 |  |
| 1554411_at | CTNNB1 | catenin (cadherin-associated protein), beta 1, 88kDa | 0,3954367 | 0,76211 | -1,44693 | 2,53333 |  |
| 1554661_s_at | C1orf71 | chromosome 1 open reading frame 71 | 0,08288 | 1,07585 | -1,44841 | 4,2202 |  |
| 209954_x_at | SS18 | synovial sarcoma translocation, chromosome 18 | 0,8884612 | 0,27164 | -1,45013 | 3,25444 |  |
| 223092_at | ANKH | ankylosis, progressive homolog (mouse) | 1,0325743 | 0,12782 | -1,45049 | 3,25444 |  |
| 217173_s_at | LDLR | low density lipoprotein receptor (familial hypercholesterolemia) | 1,3462153 | -0,18558 | -1,4508 | 0,43903 |  |
| 209288_s_at | CDC42EP3 | CDC42 effector protein (Rho GTPase binding) 3 | 0,1274218 | 1,03385 | -1,45158 | 1,43745 |  |
| 1558679_at | LOC284804 | hypothetical protein LOC284804 | -0,321839 | 1,48396 | -1,45265 | 2,53333 |  |
| 238149_at | FLJ46385 | FLJ46385 protein | 0,2270877 | 0,93613 | -1,45402 | 4,2202 |  |
| 207980_s_at | CITED2 | Cbp/p300-interacting transactivator, with Glu/Asp-rich carboxy-terminal domain, 2 | 1,1655144 | -0,00069 | -1,45602 | 0,60662 |  |
| 202952_s_at | ADAM12 | ADAM metallopeptidase domain 12 (meltrin alpha) | -0,08511 | 1,25003 | -1,45615 | 1,90223 |  |
| 208744_x_at | HSPH1 | heat shock 105kDa/110kDa protein 1 | 0,3859227 | 0,7822 | -1,46015 | 1,90223 |  |
| 238946_at | C11orf54 | Chromosome 11 open reading frame 54 | -0,306871 | 1,47661 | -1,46218 | 1,90223 |  |
| 224468_s_at | C19orf48 | chromosome 19 open reading frame 48 /// chromosome 19 open reading frame 48 | 0,4825611 | 0,68932 | -1,46485 | 3,25444 |  |
| 200801_x_at | ACTB | actin, beta | 0,5712937 | 0,60275 | -1,46755 | 1,06427 |  |
| 202274_at | ACTG2 | actin, gamma 2, smooth muscle, enteric | 0,3769253 | 0,79812 | -1,46881 | 2,53333 |  |
| 201645_at | TNC | tenascin C (hexabrachion) | 0,9133924 | 0,26214 | -1,46941 | 0,82259 |  |
| 206551_x_at | KLHL24 | kelch-like 24 (Drosophila) | 0,9745594 | 0,20386 | -1,47303 | 1,43745 |  |
| 203439_s_at | STC2 | stanniocalcin 2 | 0,3909372 | 0,78911 | -1,47506 | 3,25444 |  |
| 239289_x_at | MTMR15 | myotubularin related protein 15 | 1,3323485 | -0,15079 | -1,47694 | 1,90223 |  |
| 226267_at | JDP2 | jun dimerization protein 2 | 0,1496775 | 1,032 | -1,47709 | 1,43745 |  |
| 219260_s_at | C17orf81 | chromosome 17 open reading frame 81 | 1,5932412 | -0,41089 | -1,47794 | 0,60662 |  |
| 234972_at | ARL16 | ADP-ribosylation factor-like 16 | 0,2569713 | 0,92667 | -1,47955 | 4,2202 |  |
| 243805_at | CCBE1 | collagen and calcium binding EGF domains 1 | 0,4747822 | 0,70911 | -1,47987 | 3,25444 |  |
| 211658_at | PRDX2 | peroxiredoxin 2 /// peroxiredoxin 2 | 0,8523568 | 0,33284 | -1,4815 | 1,43745 |  |
| 1555229_a_at | C1S | complement component 1, s subcomponent | 1,0088761 | 0,17858 | -1,48433 | 2,53333 |  |
| 1560562_a_at | ZNF677 | Zinc finger protein 677 | 1,0869459 | 0,10122 | -1,4852 | 3,25444 |  |
| 210377_at | ACSM3 | acyl-CoA synthetase medium-chain family member 3 | 0,4694369 | 0,72137 | -1,48851 | 4,2202 |  |
| 212148_at | PBX1 | Pre-B-cell leukemia transcription factor 1 | 0,3268353 | 0,86846 | -1,49412 | 1,90223 |  |
| 203952_at | ATF6 | activating transcription factor 6 | 0,0251274 | 1,17195 | -1,49634 | 1,06427 |  |
| 1556613_s_at | LOC203107 | hypothetical protein LOC203107 | 0,0819158 | 1,11712 | -1,49879 | 1,06427 |  |
| 204471_at | GAP43 | growth associated protein 43 | 0,3537233 | 0,84686 | -1,50073 | 1,90223 |  |
| 227544_at | C14orf83 | chromosome 14 open reading frame 83 | -0,554332 | 1,75521 | -1,5011 | 0,06315 |  |
| 220955_x_at | RAB23 | RAB23, member RAS oncogene family | 0,0231446 | 1,18108 | -1,50528 | 3,25444 |  |
| 204971_at | CSTA | cystatin A (stefin A) | 0,2661006 | 0,93892 | -1,50628 | 4,2202 |  |
| 1552309_a_at | NEXN | nexilin (F actin binding protein) | -0,579349 | 1,78795 | -1,51075 | 0,06315 |  |
| 224159_x_at | TRIM4 | tripartite motif-containing 4 | 0,2177646 | 0,9922 | -1,51246 | 1,90223 |  |
| 242858_at | C14orf2 | Chromosome 14 open reading frame 2 | -0,547553 | 1,7577 | -1,51268 | 1,90223 |  |
| 201282_at | OGDH | oxoglutarate (alpha-ketoglutarate) dehydrogenase (lipoamide) | 0,8494345 | 0,3671 | -1,52067 | 2,53333 |  |
| 210445_at | FABP6 | fatty acid binding protein 6, ileal (gastrotropin) | 0,9139961 | 0,30296 | -1,52119 | 1,06427 |  |
| 226565_at | TMEM99 | transmembrane protein 99 | -0,215742 | 1,4338 | -1,52257 | 0,60662 |  |
| 215549_x_at | LOC643854 | similar to CTAGE family, member 5 | 0,7699203 | 0,4486 | -1,52315 | 2,53333 |  |
| 1570198_x_at | BST2 | Bone marrow stromal cell antigen 2 | 0,7022054 | 0,52055 | -1,52845 | 3,25444 |  |
| 1555419_a_at | ASAH1 | N-acylsphingosine amidohydrolase (acid ceramidase) 1 | 0,3257504 | 0,89775 | -1,52938 | 2,53333 |  |
| 207522_s_at | ATP2A3 | ATPase, Ca++ transporting, ubiquitous | 0,1968933 | | 1,0288 | -1,53211 | 1,90223 |
| 200965_s_at | ABLIM1 | actin binding LIM protein 1 | 1,0053536 | 0,22177 | -1,5339 | 1,90223 |  |
| 212143_s_at | IGFBP3 | insulin-like growth factor binding protein 3 | -0,045533 | 1,27291 | -1,53422 | 0,43903 |  |
| 233841_s_at | SUDS3 | suppressor of defective silencing 3 homolog (S. cerevisiae) | 1,2169097 | 0,01052 | -1,53429 | 3,25444 |  |
| 209243_s_at | PEG3 | paternally expressed 3 | 0,6604761 | 0,56829 | -1,53595 | 1,06427 |  |
| 222528_s_at | SLC25A37 | solute carrier family 25, member 37 | 1,1565983 | 0,07239 | -1,53623 | 2,53333 |  |
| 218729_at | LXN | latexin | 0,5715917 | 0,65884 | -1,53804 | 0,82259 |  |
| 216881_x_at | PRB4 | proline-rich protein BstNI subfamily 4 | 0,2107559 | 1,01971 | -1,53808 | 0,60662 |  |
| 202275_at | G6PD | glucose-6-phosphate dehydrogenase | 0,9057669 | 0,32579 | -1,53945 | 1,43745 |  |
| 239296_at | ZFHX1B | Zinc finger homeobox 1b | 1,1442422 | 0,09003 | -1,54284 | 1,90223 |  |
| 209409_at | GRB10 | growth factor receptor-bound protein 10 | -0,877468 | 2,11192 | -1,54306 | 0 |  |
| 210597_x_at | PRB1 /// PRB2 | proline-rich protein BstNI subfamily 1 /// proline-rich protein BstNI subfamily 2 | 0,4568121 | 0,78005 | -1,54608 | 0,82259 |  |
| 204203_at | CEBPG | CCAAT/enhancer binding protein (C/EBP), gamma | 0,9153112 | 0,32175 | -1,54633 | 1,06427 |  |
| 1558430_at | KIAA0251 | KIAA0251 protein | 1,5709745 | -0,3338 | -1,54647 | 1,90223 |  |
| 226992_at | NOSTRIN | nitric oxide synthase trafficker | 0,4783001 | 0,76205 | -1,55044 | 2,53333 |  |
| 229649_at | NRXN3 | neurexin 3 | 0,354353 | 0,88806 | -1,55301 | 3,25444 |  |
| 224428_s_at | CDCA7 | cell division cycle associated 7 /// cell division cycle associated 7 | -0,250624 | 1,49333 | -1,55338 | 0,43903 |  |
| 208894_at | HLA-DRA | major histocompatibility complex, class II, DR alpha /// major histocompatibility complex, class II, DR alpha | 1,032475 | 0,21117 | -1,55456 | 1,43745 |  |
| 233177_s_at | PNKD | paroxysmal nonkinesiogenic dyskinesia | 0,4971912 | 0,74796 | -1,55644 | 2,53333 |  |
| 219872_at | C4orf18 | chromosome 4 open reading frame 18 | 0,2475106 | 0,99787 | -1,55673 | 1,90223 |  |
| 218488_at | EIF2B3 | eukaryotic translation initiation factor 2B, subunit 3 gamma, 58kDa | 0,0777794 | 1,1688 | -1,55822 | 1,90223 |  |
| 209297_at | ITSN1 | intersectin 1 (SH3 domain protein) | -0,096422 | 1,34432 | -1,55988 | 0,43903 |  |
| 212807_s_at | SORT1 | sortilin 1 | 0,5980081 | 0,65104 | -1,56131 | 3,25444 |  |
| 235927_at | THRB | thyroid hormone receptor, beta (erythroblastic leukemia viral (v-erb-a) oncogene homolog 2, avian) | 0,9433147 | 0,30831 | -1,56454 | 0,82259 |  |
| 238893_at | LOC338758 | hypothetical protein LOC338758 | -0,122722 | 1,3757 | -1,56622 | 0,60662 |  |
| 203264_s_at | ARHGEF9 | Cdc42 guanine nucleotide exchange factor (GEF) 9 | -0,195534 | 1,44878 | -1,56656 | 1,90223 |  |
| 211685_s_at | NCALD | neurocalcin delta /// neurocalcin delta | -0,119189 | 1,37445 | -1,56908 | 0,82259 |  |
| 214931_s_at | SRPK2 | SFRS protein kinase 2 | 0,0821716 | 1,17466 | -1,57104 | 3,25444 |  |
| 225420_at | GPAM | glycerol-3-phosphate acyltransferase, mitochondrial | 0,0601813 | 1,19695 | -1,57142 | 1,43745 |  |
| 224311_s_at | CAB39 | calcium binding protein 39 | -0,007078 | 1,26432 | -1,57156 | 3,25444 |  |
| 242873_at | KLRC4 | Killer cell lectin-like receptor subfamily C, member 4 | 0,7883445 | 0,46901 | -1,57169 | 0,82259 |  |
| 231806_s_at | STK36 | serine/threonine kinase 36, fused homolog (Drosophila) | -0,069237 | 1,32799 | -1,57344 | 3,25444 |  |
| 205651_x_at | RAPGEF4 | Rap guanine nucleotide exchange factor (GEF) 4 | 0,4577799 | 0,80196 | -1,57467 | 0,82259 |  |
| 212812_at | SERINC5 | Serine incorporator 5 | 0,2769747 | 0,98369 | -1,57583 | 0,82259 |  |
| 238434_at | SMCR8 | Homo sapiens, clone IMAGE:5500209, mRNA | 0,4515782 | 0,81162 | -1,579 | 3,25444 |  |
| 200907_s_at | PALLD | palladin, cytoskeletal associated protein | 0,2089389 | 1,05629 | -1,58154 | 0,43903 |  |
| 231202_at | ALDH1L2 | aldehyde dehydrogenase 1 family, member L2 | 0,0588637 | 1,20654 | -1,58175 | 1,06427 |  |
| 215151_at | DOCK10 | dedicator of cytokinesis 10 | 1,7141443 | -0,44829 | -1,58232 | 1,90223 |  |
| 222130_s_at | FTSJ2 | FtsJ homolog 2 (E. coli) | 0,1297628 | 1,13738 | -1,58393 | 2,53333 |  |
| 204029_at | CELSR2 | cadherin, EGF LAG seven-pass G-type receptor 2 (flamingo homolog, Drosophila) | -0,069933 | 1,3386 | -1,58584 | 2,53333 |  |
| 212279_at | TMEM97 | transmembrane protein 97 | 0,7581808 | 0,51125 | -1,58679 | 0,60662 |  |
| 209301_at | CA2 | carbonic anhydrase II | 1,2510208 | 0,02344 | -1,59308 | 0,43903 |  |
| 228082_at | ASAM | adipocyte-specific adhesion molecule | -0,133976 | 1,41025 | -1,59535 | 0,60662 |  |
| 214472_at | HIST1H3D | histone cluster 1, H3d | 0,5474321 | 0,72907 | -1,59562 | 3,25444 |  |
| 213109_at | TNIK | TRAF2 and NCK interacting kinase | 0,9761012 | 0,30189 | -1,59749 | 1,43745 |  |
| 1563745_a_at | LOC283050 | hypothetical protein LOC283050 | 0,7245564 | 0,5553 | -1,59982 | 2,53333 |  |
| 210458_s_at | TANK | TRAF family member-associated NFKB activator | 1,2366938 | 0,04761 | -1,60538 | 4,2202 |  |
| 226310_at | RICTOR | rapamycin-insensitive companion of mTOR | 0,5419774 | 0,74243 | -1,6055 | 3,25444 |  |
| 205538_at | CORO2A | coronin, actin binding protein, 2A | 0,4422201 | 0,84611 | -1,61041 | 2,53333 |  |
| 202728_s_at | LTBP1 | latent transforming growth factor beta binding protein 1 | 0,3611947 | 0,92874 | -1,61242 | 0,82259 |  |
| 210785_s_at | C1orf38 | chromosome 1 open reading frame 38 | 0,983228 | 0,30775 | -1,61373 | 2,53333 |  |
| 1555123_at | ST6GAL2 | ST6 beta-galactosamide alpha-2,6-sialyltranferase 2 | -0,097061 | 1,3881 | -1,61379 | 1,06427 |  |
| 202672_s_at | ATF3 | activating transcription factor 3 | 0,5949313 | 0,69619 | -1,6139 | 0,82259 |  |
| 216387_x_at | LOC390411 | similar to nucleophosmin 1 isoform 1 | -0,567844 | 1,86075 | -1,61614 | 0,06315 |  |
| 220940_at | KIAA1641 | KIAA1641 | 0,7691304 | 0,52391 | -1,6163 | 1,43745 |  |
| 222942_s_at | TIAM2 | T-cell lymphoma invasion and metastasis 2 | 0,3683795 | 0,92561 | -1,61749 | 2,53333 |  |
| 221094_s_at | ELP3 | elongation protein 3 homolog (S. cerevisiae) | 0,035417 | 1,25916 | -1,61823 | 1,90223 |  |
| 1555384_a_at | LARP4 | La ribonucleoprotein domain family, member 4 | 1,0209821 | 0,27428 | -1,61907 | 3,25444 |  |
| 227510_x_at | PRO1073 | PRO1073 protein | 0,9848529 | 0,31161 | -1,62058 | 1,43745 |  |
| 211748_x_at | PTGDS | prostaglandin D2 synthase 21kDa (brain) /// prostaglandin D2 synthase 21kDa (brain) | 0,5261214 | 0,77047 | -1,62074 | 1,90223 |  |
| 225525_at | CTA-221G9.4 | KIAA1671 protein | 0,4896384 | 0,80773 | -1,62171 | 4,2202 |  |
| 220173_at | C14orf45 | chromosome 14 open reading frame 45 | 0,2438421 | 1,0545 | -1,62293 | 1,90223 |  |
| 1553252_a_at | BRWD3 | bromodomain and WD repeat domain containing 3 | 0,3866253 | 0,91176 | -1,62298 | 4,2202 |  |
| 213348_at | CDKN1C | Cyclin-dependent kinase inhibitor 1C (p57, Kip2) | -0,514714 | 1,81594 | -1,62653 | 0,06315 |  |
| 211016_x_at | HSPA4 | heat shock 70kDa protein 4 | 0,6164865 | 0,68505 | -1,62691 | 3,25444 |  |
| 208042_at | AGGF1 | angiogenic factor with G patch and FHA domains 1 | 0,7999449 | 0,50604 | -1,63248 | 1,90223 |  |
| 209856_x_at | ABI2 | abl interactor 2 | -0,219388 | 1,52742 | -1,63504 | 0,60662 |  |
| 216202_s_at | SPTLC2 | serine palmitoyltransferase, long chain base subunit 2 | 0,2854994 | 1,02334 | -1,63605 | 1,90223 |  |
| 217025_s_at | DBN1 | drebrin 1 | -0,332878 | 1,64259 | -1,63714 | 0,60662 |  |
| 203481_at | C10orf6 | chromosome 10 open reading frame 6 | 0,7650328 | 0,54594 | -1,63871 | 2,53333 |  |
| 221063_x_at | RNF123 | ring finger protein 123 | -0,055139 | 1,37028 | -1,64392 | 1,43745 |  |
| 217777_s_at | PTPLAD1 /// LOC732402 | protein tyrosine phosphatase-like A domain containing 1 /// similar to butyrate-induced transcript 1 | 0,5143114 | 0,80352 | -1,64728 | 1,90223 |  |
| 225473_at | C20orf117 | chromosome 20 open reading frame 117 | -0,032087 | 1,35055 | -1,64808 | 1,06427 |  |
| 202079_s_at | TRAK1 | trafficking protein, kinesin binding 1 | 0,230404 | 1,09035 | -1,65094 | 2,53333 |  |
| 230659_at | EDEM1 | ER degradation enhancer, mannosidase alpha-like 1 | 0,2098853 | 1,11166 | -1,65194 | 1,43745 |  |
| 208916_at | SLC1A5 | solute carrier family 1 (neutral amino acid transporter), member 5 | 0,1368596 | 1,18512 | -1,65248 | 0,82259 |  |
| 224521_s_at | CCDC77 | coiled-coil domain containing 77 /// coiled-coil domain containing 77 | -0,198589 | 1,52227 | -1,6546 | 2,53333 |  |
| 206672_at | AQP2 | aquaporin 2 (collecting duct) | 0,8426295 | 0,48138 | -1,65501 | 0,82259 |  |
| 222641_s_at | C17orf63 | chromosome 17 open reading frame 63 | 1,6313948 | -0,30689 | -1,65563 | 0,82259 |  |
| 224170_s_at | TULP4 | tubby like protein 4 | -0,062578 | 1,38745 | -1,65609 | 3,25444 |  |
| 209002_s_at | CALCOCO1 | calcium binding and coiled-coil domain 1 | 0,2808562 | 1,04462 | -1,65685 | 1,90223 |  |
| 211222_s_at | HAP1 | huntingtin-associated protein 1 (neuroan 1) | 1,2302112 | 0,09597 | -1,65773 | 4,2202 |  |
| 206959_s_at | UPF3A | UPF3 regulator of nonsense transcripts homolog A (yeast) | 1,645593 | -0,31748 | -1,66015 | 1,06427 |  |
| 232247_at | ZNF502 | zinc finger protein 502 | 1,0698969 | 0,25846 | -1,66044 | 1,90223 |  |
| 205403_at | IL1R2 | interleukin 1 receptor, type II | 0,847734 | 0,48205 | -1,66222 | 1,43745 |  |
| 204309_at | CYP11A1 | cytochrome P450, family 11, subfamily A, polypeptide 1 /// cytochrome P450, family 11, subfamily A, polypeptide 1 | 0,5212343 | 0,81204 | -1,66659 | 0,43903 |  |
| 202954_at | PAK3 /// UBE2C | p21 (CDKN1A)-activated kinase 3 /// ubiquitin-conjugating enzyme E2C | 0,8667653 | 0,46672 | -1,66686 | 1,90223 |  |
| 209497_s_at | RBM4B | RNA binding motif protein 4B | 1,1674518 | 0,16772 | -1,66897 | 1,43745 |  |
| 1553108_at | C5orf24 | chromosome 5 open reading frame 24 | -0,079826 | 1,41562 | -1,66974 | 1,90223 |  |
| 205996_s_at | AK2 | adenylate kinase 2 | 1,0407635 | 0,29597 | -1,67091 | 3,25444 |  |
| 208180_s_at | HIST1H4H | histone cluster 1, H4h | 0,2081021 | 1,12913 | -1,67154 | 3,25444 |  |
| 223195_s_at | SESN2 | sestrin 2 | 1,0221836 | 0,31512 | -1,67163 | 0,43903 |  |
| 242459_at | C1orf141 | Chromosome 1 open reading frame 141 | 0,9886826 | 0,34912 | -1,67225 | 0,60662 |  |
| 201988_s_at | CREBL2 | cAMP responsive element binding protein-like 2 | -0,296636 | 1,63502 | -1,67298 | 0,33143 |  |
| 236281_x_at | HTR7 | 5-hydroxytryptamine (serotonin) receptor 7 (adenylate cyclase-coupled) | 0,2893126 | 1,05009 | -1,67426 | 0,60662 |  |
| 227820_at | EBP | Emopamil binding protein (sterol isomerase) | 0,831567 | 0,50784 | -1,67426 | 1,90223 |  |
| 219596_at | THAP10 | THAP domain containing 10 | 0,3670729 | 0,97294 | -1,67502 | 3,25444 |  |
| 212256_at | GALNT10 | UDP-N-acetyl-alpha-D-galactosamine:polypeptide N-acetylgalactosaminyltransferase 10 (GalNAc-T10) | 1,1453475 | 0,19757 | -1,67865 | 1,90223 |  |
| 220694_at | DDEF1IT1 | DDEF1 intronic transcript 1 | 0,5837058 | 0,75938 | -1,67886 | 4,2202 |  |
| 209339_at | SIAH2 | seven in absentia homolog 2 (Drosophila) /// seven in absentia homolog 2 (Drosophila) | 0,9286773 | 0,41522 | -1,67987 | 1,06427 |  |
| 228968_at | ZNF449 | zinc finger protein 449 | 0,6799449 | 0,66519 | -1,68142 | 2,53333 |  |
| 204083_s_at | TPM2 | tropomyosin 2 (beta) | 0,7600255 | 0,58575 | -1,68222 | 0,43903 |  |
| 202064_s_at | SEL1L | sel-1 suppressor of lin-12-like (C. elegans) | 0,5406146 | 0,80805 | -1,68583 | 3,25444 |  |
| 222041_at | DPH1 /// OVCA2 | DPH1 homolog (S. cerevisiae) /// candidate tumor suppressor in ovarian cancer 2 | 1,4785799 | -0,12864 | -1,68743 | 1,43745 |  |
| 207501_s_at | FGF12 | fibroblast growth factor 12 | 0,4064496 | 0,94437 | -1,68852 | 1,43745 |  |
| 203988_s_at | FUT8 | fucosyltransferase 8 (alpha (1,6) fucosyltransferase) | 0,2737953 | 1,07781 | -1,6895 | 1,90223 |  |
| 203830_at | C17orf75 | chromosome 17 open reading frame 75 | 0,0547635 | 1,29739 | -1,69019 | 0,82259 |  |
| 222196_at | LOC286434 /// LOC728146 /// LOC729137 | hypothetical protein LOC286434 /// hypothetical protein LOC728146 /// hypothetical protein LOC729137 | 0,9020898 | 0,45041 | -1,69063 | 3,25444 |  |
| 217167_x_at | GK | glycerol kinase | -0,345928 | 1,69992 | -1,69249 | 0,43903 |  |
| 242617_at | GSTZ1 | Glutathione transferase zeta 1 (maleylacetoacetate isomerase) | 1,0357346 | 0,32123 | -1,69621 | 0,60662 |  |
| 1553972_a_at | CBS | cystathionine-beta-synthase | -0,231542 | 1,58873 | -1,69649 | 0,25444 |  |
| 209712_at | SLC35D1 | solute carrier family 35 (UDP-glucuronic acid/UDP-N-acetylgalactosamine dual transporter), member D1 | 0,6247417 | 0,73602 | -1,70095 | 0,43903 |  |
| 205822_s_at | HMGCS1 | 3-hydroxy-3-methylglutaryl-Coenzyme A synthase 1 (soluble) | 0,9134178 | 0,44821 | -1,70203 | 0,43903 |  |
| 227882_at | FKRP | fukutin related protein | -0,163703 | 1,52903 | -1,70666 | 0,60662 |  |
| 230167_at | ADAMTS14 | ADAM metallopeptidase with thrombospondin type 1 motif, 14 | 0,5563474 | 0,80948 | -1,70728 | 1,43745 |  |
| 226029_at | VANGL2 | vang-like 2 (van gogh, Drosophila) | -0,384608 | 1,75308 | -1,7106 | 1,06427 |  |
| 229545_at | C20orf42 | chromosome 20 open reading frame 42 | -0,045553 | 1,4143 | -1,71093 | 0,82259 |  |
| 205271_s_at | CCRK | cell cycle related kinase | 0,1894089 | 1,18068 | -1,71261 | 1,90223 |  |
| 204293_at | SGSH | N-sulfoglucosamine sulfohydrolase (sulfamidase) | 0,6535749 | 0,71791 | -1,71435 | 1,90223 |  |
| 243196_s_at | TRAFD1 | TRAF-type zinc finger domain containing 1 | 0,2108092 | 1,1618 | -1,71576 | 2,53333 |  |
| 211148_s_at | ANGPT2 | angiopoietin 2 | 0,1457478 | 1,22697 | -1,7159 | 0,82259 |  |
| 225753_at | ZNF513 | zinc finger protein 513 | 0,0908149 | 1,28318 | -1,71749 | 2,53333 |  |
| 202101_s_at | RALB | v-ral simian leukemia viral oncogene homolog B (ras related; GTP binding protein) | 0,0673328 | 1,30767 | -1,71875 | 0,60662 |  |
| 235917_at | C9orf80 | Chromosome 9 open reading frame 80 | 0,800039 | 0,57511 | -1,71893 | 4,2202 |  |
| 207891_s_at | UCHL5IP | UCHL5 interacting protein | 1,0880216 | 0,28766 | -1,7196 | 4,2202 |  |
| 243432_at | LOC642891 /// LOC649158 | hypothetical LOC642891 /// hypothetical protein LOC649158 | 0,1700356 | 1,20772 | -1,7222 | 3,25444 |  |
| 220770_s_at | LOC63920 | transposon-derived Buster3 transposase-like | 0,6850203 | 0,69325 | -1,72284 | 2,53333 |  |
| 234624_at | HHLA2 | HERV-H LTR-associating 2 | -0,208374 | 1,58794 | -1,72445 | 2,53333 |  |
| 218555_at | ANAPC2 | anaphase promoting complex subunit 2 | 0,2587885 | 1,12095 | -1,72467 | 4,2202 |  |
| 201850_at | CAPG | capping protein (actin filament), gelsolin-like | -0,234544 | 1,61435 | -1,72476 | 0,43903 |  |
| 219447_s_at | SLC35C2 | solute carrier family 35, member C2 | 0,0716029 | 1,30839 | -1,72499 | 1,06427 |  |
| 219331_s_at | KLHDC8A | kelch domain containing 8A | -0,097861 | 1,47801 | -1,72519 | 1,43745 |  |
| 229846_s_at | MAPKAP1 | mitogen-activated protein kinase associated protein 1 | 0,4072526 | 0,97291 | -1,7252 | 3,25444 |  |
| 201373_at | PLEC1 | plectin 1, intermediate filament binding protein 500kDa | 0,2198204 | 1,16089 | -1,72588 | 1,90223 |  |
| 232397_at | HECW2 | HECT, C2 and WW domain containing E3 ubiquitin protein ligase 2 | 1,16477 | 0,21698 | -1,72718 | 0,25444 |  |
| 217478_s_at | HLA-DMA | major histocompatibility complex, class II, DM alpha | 1,6139995 | -0,23152 | -1,7281 | 0,82259 |  |
| 217897_at | FXYD6 | FXYD domain containing ion transport regulator 6 | 0,7043321 | 0,67888 | -1,72902 | 1,06427 |  |
| 224724_at | SULF2 | sulfatase 2 | 0,6805725 | 0,70414 | -1,73089 | 0,60662 |  |
| 1559883_s_at | SAMHD1 | SAM domain and HD domain 1 | 0,9647081 | 0,42036 | -1,73133 | 1,43745 |  |
| 229340_at | C6orf194 | chromosome 6 open reading frame 194 | 1,151285 | 0,23446 | -1,73218 | 3,25444 |  |
| 220975_s_at | C1QTNF1 | C1q and tumor necrosis factor related protein 1 /// C1q and tumor necrosis factor related protein 1 | -0,075569 | 1,46227 | -1,73338 | 0,25444 |  |
| 223894_s_at | FTS | fused toes homolog (mouse) | 1,4310696 | -0,04425 | -1,73352 | 3,25444 |  |
| 218145_at | TRIB3 | tribbles homolog 3 (Drosophila) | 0,3804682 | 1,00813 | -1,73575 | 0,25444 |  |
| 225611_at | MAST4 | Microtubule associated serine/threonine kinase family member 4 | 0,0287895 | 1,36117 | -1,73745 | 0,16318 |  |
| 222614_at | C21orf6 | chromosome 21 open reading frame 6 | 0,3779732 | 1,01219 | -1,7377 | 3,25444 |  |
| 219905_at | ERMAP | erythroblast membrane-associated protein (Scianna blood group) | -0,255397 | 1,64584 | -1,73806 | 0,60662 |  |
| 203953_s_at | CLDN3 | claudin 3 | 0,7369976 | 0,65491 | -1,73989 | 1,90223 |  |
| 205125_at | PLCD1 | phospholipase C, delta 1 | 0,1988676 | 1,19322 | -1,7401 | 0,82259 |  |
| 206249_at | MAP3K13 | mitogen-activated protein kinase kinase kinase 13 | 0,6742302 | 0,71884 | -1,74134 | 0,60662 |  |
| 219420_s_at | C1orf163 | chromosome 1 open reading frame 163 | -0,275875 | 1,67022 | -1,74293 | 0,33143 |  |
| 243582_at | SH3RF2 | SH3 domain containing ring finger 2 | 0,0937018 | 1,30411 | -1,74727 | 2,53333 |  |
| 242126_at | EXT1 | Exostoses (multiple) 1 | 0,8102535 | 0,58787 | -1,74765 | 2,53333 |  |
| 1553142_at | C13orf31 | chromosome 13 open reading frame 31 | -0,083076 | 1,48148 | -1,74801 | 1,43745 |  |
| 216902_s_at | RRN3 /// LOC653390 /// LOC730092 | RRN3 RNA polymerase I transcription factor homolog (S. cerevisiae) /// hypothetical LOC653390 /// hypothetical protein LOC730092 | 0,4167377 | 0,98234 | -1,74884 | 1,06427 |  |
| 204290_s_at | ALDH6A1 | aldehyde dehydrogenase 6 family, member A1 | 0,3671136 | 1,03409 | -1,7515 | 0,60662 |  |
| 226071_at | ADAMTSL4 | ADAMTS-like 4 | 0,8630465 | 0,53978 | -1,75353 | 3,25444 |  |
| 223686_at | TPK1 | thiamin pyrophosphokinase 1 | 1,1591249 | 0,24579 | -1,75615 | 2,53333 |  |
| 207831_x_at | DHPS | deoxyhypusine synthase | 0,5211416 | 0,88511 | -1,75782 | 1,06427 |  |
| 202805_s_at | ABCC1 | ATP-binding cassette, sub-family C (CFTR/MRP), member 1 | -0,338145 | 1,74442 | -1,75784 | 0,60662 |  |
| 232553_at | PCYT1B | phosphate cytidylyltransferase 1, choline, beta | 0,5385517 | 0,8684 | -1,75868 | 0,82259 |  |
| 212249_at | PIK3R1 | phosphoinositide-3-kinase, regulatory subunit 1 (p85 alpha) | 0,1092647 | 1,30073 | -1,7625 | 0,60662 |  |
| 214805_at | EIF4A1 | Eukaryotic translation initiation factor 4A, isoform 1 | 1,0230778 | 0,39068 | -1,7672 | 1,90223 |  |
| 229335_at | IGSF4C | immunoglobulin superfamily, member 4C | 0,8815672 | 0,53402 | -1,76949 | 3,25444 |  |
| 222594_s_at | SPATS2 | spermatogenesis associated, serine-rich 2 | 1,5487929 | -0,1302 | -1,77325 | 0,82259 |  |
| 202444_s_at | SPFH1 | SPFH domain family, member 1 | 1,2496744 | 0,16899 | -1,77332 | 4,2202 |  |
| 229793_at | RP11-564C4.1 | N-acylsphingosine amidohydrolase (non-lysosomal ceramidase) 2B like | 0,4455909 | 0,97454 | -1,77516 | 2,53333 |  |
| 234687_x_at | LIMD1 | LIM domains containing 1 | 0,7690317 | 0,65217 | -1,7765 | 1,90223 |  |
| 204324_s_at | GOLPH4 | golgi phosphoprotein 4 | 1,3845887 | 0,03873 | -1,77915 | 1,43745 |  |
| 218013_x_at | DCTN4 | dynactin 4 (p62) | 0,1901653 | 1,23354 | -1,77963 | 0,25444 |  |
| 200672_x_at | SPTBN1 | spectrin, beta, non-erythrocytic 1 | 0,1287375 | 1,29565 | -1,78048 | 0,60662 |  |
| 213261_at | LBA1 | lupus brain antigen 1 | 0,411812 | 1,01388 | -1,78212 | 1,90223 |  |
| 1556049_at | RTN4 | reticulon 4 | 0,9897484 | 0,43658 | -1,78291 | 0,82259 |  |
| 244476_at | TJP1 | Tight junction protein 1 (zona occludens 1) | 0,9244363 | 0,50303 | -1,78434 | 4,2202 |  |
| 224324_at | MRO | maestro /// maestro | 0,7263439 | 0,70195 | -1,78537 | 0,25444 |  |
| 227511_at | SAMD4B | Sterile alpha motif domain containing 4B | 0,1065847 | 1,32215 | -1,78592 | 1,06427 |  |
| 1555963_x_at | B3GNT7 | UDP-GlcNAc:betaGal beta-1,3-N-acetylglucosaminyltransferase 7 | -0,14774 | 1,57708 | -1,78668 | 0,82259 |  |
| 1554015_a_at | CHD2 | chromodomain helicase DNA binding protein 2 | 0,2389866 | 1,19128 | -1,78784 | 1,43745 |  |
| 221754_s_at | CORO1B | coronin, actin binding protein, 1B | -0,091427 | 1,52338 | -1,78995 | 0,82259 |  |
| 213983_s_at | SCC-112 | SCC-112 protein | 0,5594662 | 0,87314 | -1,79075 | 2,53333 |  |
| 1555869_a_at | LOC729070 | Hypothetical protein LOC729070 | 0,6725248 | 0,7604 | -1,79116 | 1,06427 |  |
| 204800_s_at | DHRS12 | dehydrogenase/reductase (SDR family) member 12 | 0,1740464 | 1,2603 | -1,79293 | 1,43745 |  |
| 226303_at | PGM5 | phosphoglucomutase 5 | 0,6926984 | 0,74238 | -1,79385 | 1,90223 |  |
| 206184_at | CRKL | v-crk sarcoma virus CT10 oncogene homolog (avian)-like | 0,5916276 | 0,84422 | -1,79481 | 2,53333 |  |
| 209761_s_at | SP110 | SP110 nuclear body protein | 1,0952872 | 0,34061 | -1,79487 | 0,43903 |  |
| 215489_x_at | HOMER3 | homer homolog 3 (Drosophila) | -0,09275 | 1,53025 | -1,79688 | 0,43903 |  |
| 218789_s_at | C11orf71 | chromosome 11 open reading frame 71 | 0,3069317 | 1,13272 | -1,79956 | 3,25444 |  |
| 205652_s_at | TTLL1 | tubulin tyrosine ligase-like family, member 1 | 0,592533 | 0,84739 | -1,7999 | 2,53333 |  |
| 214577_at | MAP1B | microtubule-associated protein 1B | 0,355135 | 1,08646 | -1,802 | 0,43903 |  |
| 203924_at | GSTA1 | glutathione S-transferase A1 | 1,254218 | 0,18792 | -1,80267 | 0,06315 |  |
| 239448_at | SMAD3 | SMAD family member 3 | 1,6358088 | -0,19291 | -1,80362 | 0,16318 |  |
| 230027_s_at | MRPL43 | mitochondrial ribosomal protein L43 | 0,7386826 | 0,70617 | -1,80607 | 1,06427 |  |
| 241498_at | C11orf77 | chromosome 11 open reading frame 77 | 0,9361069 | 0,51049 | -1,80824 | 2,53333 |  |
| 215984_s_at | ARFRP1 | ADP-ribosylation factor related protein 1 | 0,681173 | 0,76566 | -1,80854 | 1,90223 |  |
| 226619_at | SENP1 | SUMO1/sentrin specific peptidase 1 | 0,2877582 | 1,15936 | -1,8089 | 2,53333 |  |
| 211949_s_at | NOLC1 | nucleolar and coiled-body phosphoprotein 1 | 0,6313706 | 0,81819 | -1,81195 | 1,43745 |  |
| 203796_s_at | BCL7A | B-cell CLL/lymphoma 7A | 0,31416 | 1,13626 | -1,81302 | 1,06427 |  |
| 241393_at | IPP | Intracisternal A particle-promoted polypeptide | 0,6495819 | 0,8015 | -1,81385 | 2,53333 |  |
| 203022_at | RNASEH2A | ribonuclease H2, subunit A | 0,8515448 | 0,59999 | -1,81442 | 1,06427 |  |
| 217543_s_at | MBTPS1 | membrane-bound transcription factor peptidase, site 1 | 0,1185168 | 1,33438 | -1,81612 | 0,43903 |  |
| 219657_s_at | KLF3 | Kruppel-like factor 3 (basic) | -0,092155 | 1,54734 | -1,81898 | 0,82259 |  |
| 202382_s_at | GNPDA1 | glucosamine-6-phosphate deaminase 1 | 0,5410001 | 0,91669 | -1,82211 | 0,43903 |  |
| 200861_at | CNOT1 | CCR4-NOT transcription complex, subunit 1 | 1,1339005 | 0,32472 | -1,82328 | 1,06427 |  |
| 207265_s_at | KDELR3 | KDEL (Lys-Asp-Glu-Leu) endoplasmic reticulum protein retention receptor 3 | -0,270738 | 1,73073 | -1,82499 | 0,25444 |  |
| 220028_at | ACVR2B | activin A receptor, type IIB | 0,0784125 | 1,38293 | -1,82668 | 1,06427 |  |
| 202742_s_at | PRKACB | protein kinase, cAMP-dependent, catalytic, beta | 0,0021865 | 1,45961 | -1,82725 | 2,53333 |  |
| 212064_x_at | MAZ | MYC-associated zinc finger protein (purine-binding transcription factor) | 1,2292275 | 0,23268 | -1,82738 | 0,43903 |  |
| 1552485_at | LACTB | lactamase, beta | 0,4740017 | 0,98873 | -1,82842 | 1,06427 |  |
| 211089_s_at | NEK3 | NIMA (never in mitosis gene a)-related kinase 3 /// NIMA (never in mitosis gene a)-related kinase 3 | 0,1251938 | 1,3402 | -1,83174 | 1,43745 |  |
| 1556730_at | LOC652993 | hypothetical LOC652993 | 0,2797462 | 1,18665 | -1,83299 | 1,90223 |  |
| 212259_s_at | PBXIP1 | pre-B-cell leukemia transcription factor interacting protein 1 | 1,1240764 | 0,34415 | -1,83528 | 0,82259 |  |
| 209006_s_at | C1orf63 | chromosome 1 open reading frame 63 | 0,8994305 | 0,56971 | -1,83643 | 0,82259 |  |
| 215506_s_at | DIRAS3 | DIRAS family, GTP-binding RAS-like 3 | -0,197918 | 1,66724 | -1,83665 | 0 |  |
| 1555009_a_at | SYNJ2 | synaptojanin 2 | 1,0716704 | 0,39961 | -1,8391 | 4,2202 |  |
| 214870_x_at | NPIP /// LOC339047 /// LOC642799 | nuclear pore complex interacting protein /// hypothetical protein LOC339047 /// similar to nuclear pore complex interacting protein | 0,6504859 | 0,82233 | -1,84102 | 0,25444 |  |
| 215022_x_at | ZNF33B | zinc finger protein 33B | 0,5762714 | 0,89655 | -1,84103 | 3,25444 |  |
| 209488_s_at | RBPMS | RNA binding protein with multiple splicing | 0,0203307 | 1,45362 | -1,84243 | 0,33143 |  |
| 231989_s_at | LOC440345 /// LOC641298 /// LOC730099 | hypothetical protein LOC440345 /// PI-3-kinase-related kinase SMG-1 - like locus /// similar to PI-3-kinase-related kinase SMG-1 | 0,5986488 | 0,87573 | -1,84298 | 3,25444 |  |
| 218607_s_at | SDAD1 | SDA1 domain containing 1 | 0,205282 | 1,26973 | -1,84376 | 0,43903 |  |
| 233380_s_at | RUFY1 | RUN and FYVE domain containing 1 | 0,7945485 | 0,6817 | -1,84532 | 1,43745 |  |
| 1557064_s_at | HGSNAT | heparan-alpha-glucosaminide N-acetyltransferase | -0,275026 | 1,75203 | -1,84625 | 1,43745 |  |
| 223821_s_at | SUSD4 | sushi domain containing 4 | 0,223898 | 1,25319 | -1,84636 | 0,33143 |  |
| 1552264_a_at | MAPK1 | mitogen-activated protein kinase 1 | 0,5118659 | 0,96614 | -1,84751 | 1,06427 |  |
| 212135_s_at | ATP2B4 | ATPase, Ca++ transporting, plasma membrane 4 | -0,294493 | 1,77354 | -1,84881 | 0 |  |
| 1554894_a_at | PCBD2 | pterin-4 alpha-carbinolamine dehydratase/dimerization cofactor of hepatocyte nuclear factor 1 alpha (TCF1) 2 | 0,5498686 | 0,92989 | -1,84969 | 3,25444 |  |
| 202407_s_at | PRPF31 | PRP31 pre-mRNA processing factor 31 homolog (S. cerevisiae) | 0,6190046 | 0,86241 | -1,85177 | 3,25444 |  |
| 231842_at | KIAA1462 | KIAA1462 | 0,2607496 | 1,22286 | -1,85452 | 0,16318 |  |
| 227007_at | TMCO4 | transmembrane and coiled-coil domains 4 | 1,0993915 | 0,38696 | -1,85794 | 1,90223 |  |
| 205333_s_at | RCE1 | RCE1 homolog, prenyl protein peptidase (S. cerevisiae) | 1,3511005 | 0,13704 | -1,86018 | 0,60662 |  |
| 224609_at | SLC44A2 | solute carrier family 44, member 2 | 0,1206783 | 1,36974 | -1,86302 | 0,33143 |  |
| 224522_s_at | DCAKD | dephospho-CoA kinase domain containing /// dephospho-CoA kinase domain containing | 0,1072857 | 1,38344 | -1,8634 | 0,60662 |  |
| 222635_s_at | MED28 | mediator of RNA polymerase II transcription, subunit 28 homolog (S. cerevisiae) | -0,144277 | 1,636 | -1,86465 | 0,16318 |  |
| 227978_s_at | ZADH2 | zinc binding alcohol dehydrogenase, domain containing 2 | 0,473774 | 1,01819 | -1,86496 | 1,90223 |  |
| 1556462_a_at | LOC730245 | Hypothetical protein LOC730245 | 0,9936616 | 0,49894 | -1,86575 | 1,90223 |  |
| 238574_at | MCART1 | Mitochondrial carrier triple repeat 1 | 0,5258552 | 0,9671 | -1,86619 | 1,06427 |  |
| 218094_s_at | DBNDD2 /// C20orf169-DBNDD2 | dysbindin (dystrobrevin binding protein 1) domain containing 2 /// C20orf169-DBNDD2 readthrough transcript | 0,2180455 | 1,2755 | -1,86694 | 0,16318 |  |
| 223496_s_at | CCDC8 | coiled-coil domain containing 8 | -0,037221 | 1,53357 | -1,87044 | 1,06427 |  |
| 203572_s_at | TAF6 | TAF6 RNA polymerase II, TATA box binding protein (TBP)-associated factor, 80kDa | 0,5469756 | 0,95137 | -1,87293 | 0,43903 |  |
| 215177_s_at | ITGA6 | integrin, alpha 6 | 0,2222326 | 1,27618 | -1,87302 | 0,33143 |  |
| 209864_at | FRAT2 | frequently rearranged in advanced T-cell lymphomas 2 | 0,3543794 | 1,14405 | -1,87303 | 1,43745 |  |
| 207177_at | PTGFR | prostaglandin F receptor (FP) | -0,705285 | 2,20607 | -1,87598 | 0 |  |
| 217857_s_at | RBM8A | RNA binding motif protein 8A | 0,2940428 | 1,2081 | -1,87767 | 0,82259 |  |
| 231894_at | SARS | Seryl-tRNA synthetase | 0,36198 | 1,1409 | -1,8786 | 0,16318 |  |
| 206110_at | HIST1H3H | histone cluster 1, H3h | 0,3904683 | 1,11281 | -1,8791 | 3,25444 |  |
| 225803_at | FBXO32 | F-box protein 32 | 0,4227887 | 1,08357 | -1,88295 | 0,06315 |  |
| 204538_x_at | NPIP /// LOC339047 /// LOC642778 /// LOC642799 | nuclear pore complex interacting protein /// hypothetical protein LOC339047 /// similar to nuclear pore complex interacting protein /// similar to nuclear pore complex interacting protein | 0,6981315 | 0,80824 | -1,88296 | 0,16318 |  |
| 222366_at | ADNP | Activity-dependent neuroprotector | 0,9567179 | 0,55136 | -1,8851 | 2,53333 |  |
| 241611_s_at | FNDC3A | fibronectin type III domain containing 3A | 0,974773 | 0,53453 | -1,88663 | 1,90223 |  |
| 226411_at | EVI5L | ecotropic viral integration site 5-like | 0,2449059 | 1,26457 | -1,88684 | 2,53333 |  |
| 220387_s_at | HHLA3 | HERV-H LTR-associating 3 | 0,1469836 | 1,36437 | -1,8892 | 4,2202 |  |
| 1558871_at | EPGN | Epithelial mitogen homolog (mouse) | 0,913741 | 0,60096 | -1,89338 | 0,43903 |  |
| 238001_at | KCTD6 | potassium channel tetramerisation domain containing 6 | 1,1697087 | 0,34936 | -1,89884 | 1,43745 |  |
| 216602_s_at | FARSLA | phenylalanine-tRNA synthetase-like, alpha subunit | 0,1217311 | 1,39859 | -1,90041 | 0,43903 |  |
| 224697_at | WDR22 | WD repeat domain 22 | 1,5530286 | -0,03212 | -1,90113 | 0,82259 |  |
| 1569629_x_at | LOC389906 | similar to Serine/threonine-protein kinase PRKX (Protein kinase PKX1) | 0,664179 | 0,85775 | -1,90241 | 2,53333 |  |
| 212654_at | TPM2 /// PPIL5 | tropomyosin 2 (beta) /// peptidylprolyl isomerase (cyclophilin)-like 5 | -0,038566 | 1,56091 | -1,90293 | 0,60662 |  |
| 214726_x_at | ADD1 | adducin 1 (alpha) | 0,7367228 | 0,78577 | -1,90312 | 0,60662 |  |
| 210655_s_at | FOXO3A | forkhead box O3A | 1,0319342 | 0,49236 | -1,90537 | 0,82259 |  |
| 232057_at | SLC7A6OS | solute carrier family 7, member 6 opposite strand | 0,8157009 | 0,7093 | -1,90625 | 1,90223 |  |
| 219368_at | NAP1L2 | nucleosome assembly protein 1-like 2 | 0,1012546 | 1,42504 | -1,90787 | 0,43903 |  |
| 202341_s_at | TRIM2 | tripartite motif-containing 2 | 0,0387878 | 1,48866 | -1,90931 | 0,82259 |  |
| 226973_at | C20orf102 | chromosome 20 open reading frame 102 | 0,1405405 | 1,38703 | -1,90947 | 0,25444 |  |
| 207821_s_at | PTK2 | PTK2 protein tyrosine kinase 2 | 0,8963196 | 0,63143 | -1,90969 | 0,82259 |  |
| 36830_at | MIPEP | mitochondrial intermediate peptidase | 0,9575358 | 0,57693 | -1,91808 | 2,53333 |  |
| 212730_at | DMN | desmuslin | -0,497112 | 2,03166 | -1,91819 | 0,06315 |  |
| 224627_at | GBA2 | glucosidase, beta (bile acid) 2 | 0,5076231 | 1,0277 | -1,91916 | 0,60662 |  |
| 210635_s_at | KLHL20 | kelch-like 20 (Drosophila) | -0,034421 | 1,57161 | -1,92148 | 2,53333 |  |
| 217337_at | LOC646677 /// LOC650674 | similar to aconitase 2, mitochondrial /// similar to aconitase 2, mitochondrial | 0,8729751 | 0,66553 | -1,92313 | 3,25444 |  |
| 202944_at | NAGA | N-acetylgalactosaminidase, alpha- | 0,8846394 | 0,65843 | -1,92884 | 2,53333 |  |
| 233274_at | NCK1 | NCK adaptor protein 1 | 1,0241236 | 0,52589 | -1,93751 | 3,25444 |  |
| 228367_at | ALPK2 | alpha-kinase 2 | 0,6196392 | 0,93209 | -1,93966 | 0,25444 |  |
| 66053_at | HNRPUL2 | heterogeneous nuclear ribonucleoprotein U-like 2 | 0,07763 | 1,47452 | -1,94019 | 3,25444 |  |
| 215629_s_at | DLEU2L | deleted in lymphocytic leukemia 2-like | 1,3558898 | 0,19818 | -1,94258 | 0,82259 |  |
| 1554509_a_at | C10orf97 | chromosome 10 open reading frame 97 | -0,136477 | 1,69319 | -1,94589 | 0,33143 |  |
| 202199_s_at | SRPK1 | SFRS protein kinase 1 | 0,2700956 | 1,28665 | -1,94594 | 1,43745 |  |
| 227782_at | ZBTB7C | zinc finger and BTB domain containing 7C | 1,6858402 | -0,12898 | -1,94608 | 0 |  |
| 238794_at | C10orf78 | chromosome 10 open reading frame 78 | 1,004526 | 0,55265 | -1,94647 | 2,53333 |  |
| 216315_x_at | UBE2V1 /// Kua-UEV /// LOC730052 /// LOC732137 | ubiquitin-conjugating enzyme E2 variant 1 /// ubiquitin-conjugating enzyme E2 variant 1 /// similar to ubiquitin-conjugating enzyme E2 variant 1 isoform d /// similar to ubiquitin-conjugating enzyme E2 variant 1 isoform d | 0,4272331 | 1,13029 | -1,94691 | 2,53333 |  |
| 216521_s_at | BRCC3 | BRCA1/BRCA2-containing complex, subunit 3 | 0,0393885 | 1,52072 | -1,95013 | 1,43745 |  |
| 218218_at | DIP13B | DIP13 beta | 0,7575069 | 0,8041 | -1,952 | 0,11715 |  |
| 210892_s_at | GTF2I | general transcription factor II, i | 0,9504591 | 0,61293 | -1,95424 | 3,25444 |  |
| 214804_at | CENPI | Centromere protein I | 1,6226957 | -0,05918 | -1,9544 | 1,43745 |  |
| 242915_at | ZNF682 | zinc finger protein 682 | 0,5651536 | 0,99897 | -1,95516 | 1,43745 |  |
| 204312_x_at | CREB1 | cAMP responsive element binding protein 1 | 1,295192 | 0,26962 | -1,95602 | 0,82259 |  |
| 210465_s_at | SNAPC3 | small nuclear RNA activating complex, polypeptide 3, 50kDa | 1,0873405 | 0,47833 | -1,95708 | 0,33143 |  |
| 201397_at | PHGDH | phosphoglycerate dehydrogenase | -0,041138 | 1,60705 | -1,9574 | 0,06315 |  |
| 209584_x_at | APOBEC3C | apolipoprotein B mRNA editing enzyme, catalytic polypeptide-like 3C | 0,5191937 | 1,04716 | -1,95794 | 0,43903 |  |
| 236574_at | LOC284373 | Hypothetical protein LOC284373 | 0,87441 | 0,69579 | -1,96275 | 0,82259 |  |
| 208024_s_at | DGCR6 /// DGCR6L | DiGeorge syndrome critical region gene 6 /// DiGeorge syndrome critical region gene 6-like | 0,6673897 | 0,90334 | -1,96341 | 0,33143 |  |
| 230685_at | FLJ33630 | hypothetical protein LOC644873 | 1,0581703 | 0,51264 | -1,96351 | 1,90223 |  |
| 228195_at | MGC13057 | hypothetical protein MGC13057 | 0,3023987 | 1,26903 | -1,96428 | 0,11715 |  |
| 201615_x_at | CALD1 | caldesmon 1 | 0,035205 | 1,53781 | -1,96627 | 0,11715 |  |
| 205730_s_at | ABLIM3 | actin binding LIM protein family, member 3 | 0,0841931 | 1,48946 | -1,96706 | 0,06315 |  |
| 205283_at | FCMD | Fukuyama type congenital muscular dystrophy (fukutin) | 0,394909 | 1,17988 | -1,96848 | 0,60662 |  |
| 209885_at | RHOD | ras homolog gene family, member D | 0,8181043 | 0,75849 | -1,97074 | 0,43903 |  |
| 223487_x_at | GNB4 | guanine nucleotide binding protein (G protein), beta polypeptide 4 | 0,894342 | 0,68321 | -1,97194 | 2,53333 |  |
| 201957_at | PPP1R12B | protein phosphatase 1, regulatory (inhibitor) subunit 12B | 0,7981916 | 0,78284 | -1,97629 | 0,11715 |  |
| 226181_at | TUBE1 | tubulin, epsilon 1 | 1,6952055 | -0,11103 | -1,98022 | 0,11715 |  |
| 221430_s_at | RNF146 | ring finger protein 146 /// ring finger protein 146 | 0,9888113 | 0,59553 | -1,98042 | 1,06427 |  |
| 224288_x_at | FKSG49 | FKSG49 | 1,2010871 | 0,3859 | -1,98374 | 0,82259 |  |
| 1554785_at | CCDC82 | coiled-coil domain containing 82 | 0,1282446 | 1,45916 | -1,98425 | 3,25444 |  |
| 233559_s_at | WDFY1 | WD repeat and FYVE domain containing 1 | 0,2247748 | 1,36317 | -1,98493 | 0,06315 |  |
| 225055_at | AMZ2 | Archaemetzincins-2 | 0,7427205 | 0,85027 | -1,99123 | 1,06427 |  |
| 1554145_a_at | CCDC128 | coiled-coil domain containing 128 | 1,1632488 | 0,43069 | -1,99242 | 1,43745 |  |
| 212128_s_at | DAG1 | dystroglycan 1 (dystrophin-associated glycoprotein 1) | 1,1851719 | 0,40884 | -1,99252 | 0,33143 |  |
| 204768_s_at | FEN1 | flap structure-specific endonuclease 1 | 0,4343642 | 1,16056 | -1,99365 | 1,06427 |  |
| 206686_at | PDK1 | pyruvate dehydrogenase kinase, isozyme 1 | 1,3418594 | 0,25314 | -1,99375 | 0,82259 |  |
| 1553162_x_at | C19orf55 | chromosome 19 open reading frame 55 | 0,0636243 | 1,53209 | -1,99464 | 0,82259 |  |
| 231723_at | SNX12 | sorting nexin 12 | 0,9009113 | 0,69783 | -1,99843 | 1,90223 |  |
| 227185_at | LOC643988 | hypothetical LOC643988 | 0,8314167 | 0,77111 | -2,00315 | 0,60662 |  |
| 223446_s_at | DTNBP1 | dystrobrevin binding protein 1 | 0,3767564 | 1,22609 | -2,00356 | 1,43745 |  |
| 211019_s_at | LSS /// PCM1 | lanosterol synthase (2,3-oxidosqualene-lanosterol cyclase) /// pericentriolar material 1 | 1,1271347 | 0,47906 | -2,00775 | 1,43745 |  |
| 1555725_a_at | UPK1B /// RGS5 | uroplakin 1B /// regulator of G-protein signalling 5 | 0,627633 | 0,9787 | -2,00792 | 0,16318 |  |
| 202833_s_at | SERPINA1 | serpin peptidase inhibitor, clade A (alpha-1 antiproteinase, antitrypsin), member 1 | 1,1627182 | 0,44424 | -2,00869 | 0,25444 |  |
| 241710_at | LOC728819 | similar to C1GALT1-specific chaperone 1 | -0,019992 | 1,62718 | -2,00899 | 0,60662 |  |
| 1552927_at | MAP3K7IP3 | mitogen-activated protein kinase kinase kinase 7 interacting protein 3 | 0,6218367 | 0,98602 | -2,00982 | 0,82259 |  |
| 206748_s_at | SPAG9 | sperm associated antigen 9 | 0,7635415 | 0,84691 | -2,01307 | 1,43745 |  |
| 203381_s_at | APOE | apolipoprotein E | -0,168192 | 1,7791 | -2,01364 | 0 |  |
| 213328_at | NEK1 | NIMA (never in mitosis gene a)-related kinase 1 | 0,1354937 | 1,47728 | -2,01597 | 0,06315 |  |
| 201012_at | ANXA1 | annexin A1 | 0,9423279 | 0,67125 | -2,01698 | 0,06315 |  |
| 213279_at | DHRS1 | dehydrogenase/reductase (SDR family) member 1 | 0,8368192 | 0,77731 | -2,01766 | 0,82259 |  |
| 225382_at | ZNF275 | zinc finger protein 275 | 0,7831303 | 0,83145 | -2,01823 | 0,11715 |  |
| 210141_s_at | INHA | inhibin, alpha | 1,4147223 | 0,20133 | -2,02007 | 0 |  |
| 206447_at | ELA2A | elastase 2A | 0,8911628 | 0,72657 | -2,02217 | 0,25444 |  |
| 205142_x_at | ABCD1 | ATP-binding cassette, sub-family D (ALD), member 1 | 0,7756381 | 0,84344 | -2,02385 | 0,60662 |  |
| 243225_at | LOC283481 | hypothetical protein LOC283481 | -0,144755 | 1,76407 | -2,02414 | 1,43745 |  |
| 232389_at | WIPF3 | WAS/WASL interacting protein family, member 3 | 0,4210529 | 1,19882 | -2,02484 | 0,11715 |  |
| 205580_s_at | HRH1 | histamine receptor H1 | 0,4767021 | 1,14334 | -2,02505 | 0,60662 |  |
| 225548_at | SHROOM3 | shroom family member 3 | 0,0376819 | 1,58339 | -2,02635 | 0,16318 |  |
| 205547_s_at | TAGLN | transgelin | 0,2569373 | 1,36766 | -2,03074 | 0,06315 |  |
| 1554464_a_at | CRTAP | cartilage associated protein | 0,5808622 | 1,04405 | -2,03114 | 0,82259 |  |
| 215714_s_at | SMARCA4 | SWI/SNF related, matrix associated, actin dependent regulator of chromatin, subfamily a, member 4 | 0,4839245 | 1,14131 | -2,03155 | 0,25444 |  |
| 230186_at | TMEM136 | transmembrane protein 136 | 0,2552364 | 1,37021 | -2,0318 | 0,43903 |  |
| 226825_s_at | TMEM165 | transmembrane protein 165 | 0,9072901 | 0,71857 | -2,03232 | 0,06315 |  |
| 206724_at | CBX4 | chromobox homolog 4 (Pc class homolog, Drosophila) | 0,0322593 | 1,59558 | -2,0348 | 0,82259 |  |
| 205516_x_at | CIZ1 | CDKN1A interacting zinc finger protein 1 | 0,3092148 | 1,32148 | -2,03837 | 0,25444 |  |
| 1552610_a_at | JAK1 | Janus kinase 1 (a protein tyrosine kinase) | 0,3758871 | 1,255 | -2,0386 | 0,16318 |  |
| 210692_s_at | SLC43A3 | solute carrier family 43, member 3 | 1,0044247 | 0,62997 | -2,043 | 0,60662 |  |
| 211212_s_at | ORC5L | origin recognition complex, subunit 5-like (yeast) | 0,9612851 | 0,67478 | -2,04509 | 1,90223 |  |
| 238935_at | RPS27L | ribosomal protein S27-like | -0,381262 | 2,02114 | -2,04985 | 0 |  |
| 209677_at | PRKCI | protein kinase C, iota | 0,7421049 | 0,89975 | -2,05232 | 1,90223 |  |
| 202796_at | SYNPO | synaptopodin | 0,3019818 | 1,34008 | -2,05258 | 0,06315 |  |
| 211813_x_at | DCN | decorin | 0,2923891 | 1,35002 | -2,05301 | 0 |  |
| 214279_s_at | NDRG2 | NDRG family member 2 | 0,5705796 | 1,07533 | -2,05739 | 0,60662 |  |
| 1553718_at | ZNF548 | zinc finger protein 548 | 0,1945921 | 1,45145 | -2,05756 | 0,16318 |  |
| 223940_x_at | MALAT1 | metastasis associated lung adenocarcinoma transcript 1 (non-coding RNA) | 1,293784 | 0,35452 | -2,06038 | 0 |  |
| 202479_s_at | TRIB2 | tribbles homolog 2 (Drosophila) | 0,4866817 | 1,16445 | -2,06392 | 0,16318 |  |
| 203027_s_at | MVD | mevalonate (diphospho) decarboxylase | 1,1199686 | 0,53157 | -2,06442 | 0,25444 |  |
| 206022_at | NDP | Norrie disease (pseudoglioma) | 0,9503363 | 0,70351 | -2,06731 | 0,06315 |  |
| 214035_x_at | LOC399491 | LOC399491 protein | 0,8423292 | 0,81343 | -2,06969 | 0,06315 |  |
| 203823_at | RGS3 | regulator of G-protein signalling 3 | 0,3464023 | 1,31081 | -2,07151 | 0,25444 |  |
| 212400_at | FAM102A | family with sequence similarity 102, member A | 1,153571 | 0,50498 | -2,07319 | 0,60662 |  |
| 209463_s_at | TAF12 | TAF12 RNA polymerase II, TATA box binding protein (TBP)-associated factor, 20kDa | 1,487469 | 0,17285 | -2,0754 | 0,16318 |  |
| 1568592_at | TRIM69 | tripartite motif-containing 69 | 0,9723693 | 0,68831 | -2,07585 | 0,11715 |  |
| 232412_at | Scrapper | CDNA: FLJ21037 fis, clone CAE10055 | 0,4012051 | 1,2601 | -2,07663 | 0,25444 |  |
| 201728_s_at | KIAA0100 /// CCDC134 | KIAA0100 /// coiled-coil domain containing 134 | 1,0205977 | 0,64161 | -2,07776 | 1,06427 |  |
| 223360_at | C21orf56 | chromosome 21 open reading frame 56 | 0,3244518 | 1,33922 | -2,07959 | 0,11715 |  |
| 238449_at | LOC595101 | PI-3-kinase-related kinase SMG-1 pseudogene | 1,4858178 | 0,17817 | -2,07998 | 0,25444 |  |
| 223417_at | RAD18 | RAD18 homolog (S. cerevisiae) | 0,5001039 | 1,16488 | -2,08123 | 1,06427 |  |
| 209230_s_at | NUPR1 | nuclear protein 1 | 0,004335 | 1,66146 | -2,08225 | 0 |  |
| 222165_x_at | C9orf16 | chromosome 9 open reading frame 16 | 0,8036833 | 0,86415 | -2,08479 | 0,16318 |  |
| 203975_s_at | CHAF1A | chromatin assembly factor 1, subunit A (p150) | 0,2070697 | 1,46099 | -2,08507 | 1,06427 |  |
| 209895_at | PTPN11 | protein tyrosine phosphatase, non-receptor type 11 (Noonan syndrome 1) | 0,6885362 | 0,97978 | -2,08539 | 0,60662 |  |
| 225582_at | KIAA1754 | KIAA1754 | 0,3417216 | 1,32839 | -2,08764 | 0,33143 |  |
| 203248_at | ZNF24 | zinc finger protein 24 | 1,2632415 | 0,40719 | -2,08805 | 0,82259 |  |
| 205214_at | STK17B | serine/threonine kinase 17b (apoptosis-inducing) | 1,716716 | -0,04584 | -2,0886 | 0 |  |
| 227622_at | PCF11 | PCF11, cleavage and polyadenylation factor subunit, homolog (S. cerevisiae) | 0,8204365 | 0,8519 | -2,09042 | 0,33143 |  |
| 225063_at | UBL7 | ubiquitin-like 7 (bone marrow stromal cell-derived) | 0,6816727 | 0,99218 | -2,09231 | 0,16318 |  |
| 201161_s_at | CSDA | cold shock domain protein A | 0,7880223 | 0,88815 | -2,09522 | 0 |  |
| 212302_at | RTF1 | Rtf1, Paf1/RNA polymerase II complex component, homolog (S. cerevisiae) | 0,7864819 | 0,89009 | -2,09571 | 0,43903 |  |
| 1554966_a_at | FILIP1L | filamin A interacting protein 1-like | 0,6391812 | 1,03923 | -2,09801 | 0,06315 |  |
| 211797_s_at | NFYC | nuclear transcription factor Y, gamma | 0,8425415 | 0,83662 | -2,09895 | 0,43903 |  |
| 201750_s_at | ECE1 | endothelin converting enzyme 1 | 0,5315784 | 1,15084 | -2,10303 | 0,25444 |  |
| 209610_s_at | SLC1A4 | solute carrier family 1 (glutamate/neutral amino acid transporter), member 4 | 0,8068115 | 0,87636 | -2,10397 | 0,11715 |  |
| 206998_x_at | PRB3 | proline-rich protein BstNI subfamily 3 | 0,1063342 | 1,57887 | -2,10651 | 0 |  |
| 226075_at | SPSB1 | splA/ryanodine receptor domain and SOCS box containing 1 | 1,4024967 | 0,28402 | -2,10814 | 0,06315 |  |
| 64900_at | FLJ22167 | hypothetical protein FLJ22167 | 0,7857928 | 0,90079 | -2,10823 | 0,33143 |  |
| 215990_s_at | BCL6 | B-cell CLL/lymphoma 6 (zinc finger protein 51) | 1,6858666 | 0,00119 | -2,10882 | 0,06315 |  |
| 220475_at | SLC28A3 | solute carrier family 28 (sodium-coupled nucleoside transporter), member 3 | 0,1763435 | 1,51195 | -2,11036 | 0 |  |
| 228141_at | LOC493869 | Similar to RIKEN cDNA 2310016C16 | 0,3292227 | 1,36093 | -2,11269 | 0,06315 |  |
| 218353_at | RGS5 | regulator of G-protein signalling 5 | 0,8508464 | 0,8397 | -2,11319 | 0,06315 |  |
| 1560386_at | XPO1 | Exportin 1 (CRM1 homolog, yeast) | 0,6580123 | 1,03293 | -2,11368 | 1,43745 |  |
| 235164_at | ZNF25 | zinc finger protein 25 | 1,5945001 | 0,09757 | -2,11509 | 0,25444 |  |
| 1562415_a_at | SPOCD1 | SPOC domain containing 1 | 1,0922357 | 0,60053 | -2,11596 | 0,06315 |  |
| 228521_s_at | RAB4B | RAB4B, member RAS oncogene family | 1,0485105 | 0,6455 | -2,11751 | 0,25444 |  |
| 227616_at | BCL9L | B-cell CLL/lymphoma 9-like | 1,4645971 | 0,23032 | -2,11865 | 1,06427 |  |
| 201376_s_at | HNRPF | heterogeneous nuclear ribonucleoprotein F | 0,8608069 | 0,83548 | -2,12036 | 0,11715 |  |
| 201564_s_at | FSCN1 | fascin homolog 1, actin-bundling protein (Strongylocentrotus purpuratus) | 0,1895531 | 1,50759 | -2,12142 | 0 |  |
| 224280_s_at | FAM54B | family with sequence similarity 54, member B | 0,4820938 | 1,21505 | -2,12143 | 0,06315 |  |
| 202226_s_at | CRK | v-crk sarcoma virus CT10 oncogene homolog (avian) | 0,5096085 | 1,18965 | -2,12408 | 0 |  |
| 221539_at | EIF4EBP1 | eukaryotic translation initiation factor 4E binding protein 1 | 0,6980457 | 1,00683 | -2,13109 | 0 |  |
| 1553155_x_at | ATP6V0D2 | ATPase, H+ transporting, lysosomal 38kDa, V0 subunit d2 | 0,5302486 | 1,17557 | -2,13227 | 2,53333 |  |
| 214276_at | KLF12 | Kruppel-like factor 12 | 0,0247406 | 1,68173 | -2,13309 | 0,06315 |  |
| 208900_s_at | TOP1 | topoisomerase (DNA) I | 1,2398763 | 0,46665 | -2,13316 | 0 |  |
| 213490_s_at | MAP2K2 | mitogen-activated protein kinase kinase 2 | 1,0850639 | 0,62264 | -2,13463 | 0,25444 |  |
| 202402_s_at | CARS | cysteinyl-tRNA synthetase | 0,1231443 | 1,58633 | -2,13685 | 0 |  |
| 212471_at | KIAA0241 | KIAA0241 | 1,3078218 | 0,40196 | -2,13722 | 1,06427 |  |
| 243026_x_at | BIRC4 | baculoviral IAP repeat-containing 4 | -0,066807 | 1,77834 | -2,13942 | 0,06315 |  |
| 211323_s_at | ITPR1 | inositol 1,4,5-triphosphate receptor, type 1 | 0,8436857 | 0,8709 | -2,14323 | 0,11715 |  |
| 210605_s_at | MFGE8 | milk fat globule-EGF factor 8 protein | 0,3629468 | 1,35422 | -2,14646 | 0,25444 |  |
| 204534_at | VTN | vitronectin | 0,6271618 | 1,09003 | -2,14649 | 0 |  |
| 207001_x_at | TSC22D3 | TSC22 domain family, member 3 | 0,2995095 | 1,41847 | -2,14748 | 0,06315 |  |
| 224659_at | SEPN1 | selenoprotein N, 1 | 0,8918527 | 0,82693 | -2,14847 | 0,43903 |  |
| 233292_s_at | ANKHD1 /// MASK-BP3 | ankyrin repeat and KH domain containing 1 /// MASK-4E-BP3 alternate reading frame gene | 0,7673628 | 0,95298 | -2,15042 | 2,53333 |  |
| 1555803_a_at | C11orf57 | chromosome 11 open reading frame 57 | -0,02408 | 1,74981 | -2,15716 | 0,16318 |  |
| 212559_at | PRKAR1B | protein kinase, cAMP-dependent, regulatory, type I, beta | 0,4526671 | 1,27388 | -2,15818 | 0,16318 |  |
| 200825_s_at | HYOU1 | hypoxia up-regulated 1 | 0,0050393 | 1,7232 | -2,1603 | 0 |  |
| 1558755_x_at | ZNF763 | zinc finger protein 763 | 0,6086712 | 1,12079 | -2,16183 | 0,60662 |  |
| 1558111_at | MBNL1 | Muscleblind-like (Drosophila) | 0,951846 | 0,77972 | -2,16446 | 1,43745 |  |
| 201785_at | RNASE1 | ribonuclease, RNase A family, 1 (pancreatic) | 0,4207896 | 1,31333 | -2,16765 | 0,06315 |  |
| 206544_x_at | SMARCA2 | SWI/SNF related, matrix associated, actin dependent regulator of chromatin, subfamily a, member 2 | -0,002259 | 1,73703 | -2,16847 | 1,06427 |  |
| 201961_s_at | RNF41 | ring finger protein 41 | 0,6662251 | 1,06898 | -2,169 | 0,33143 |  |
| 218651_s_at | LARP6 | La ribonucleoprotein domain family, member 6 | 0,7671635 | 0,9687 | -2,16984 | 0,16318 |  |
| 1554696_s_at | TYMS | thymidylate synthetase | 0,6546597 | 1,08132 | -2,16997 | 0,43903 |  |
| 238542_at | ULBP2 | UL16 binding protein 2 | 0,58954 | 1,15097 | -2,17564 | 0,06315 |  |
| 203827_at | WIPI1 | WD repeat domain, phosphoinositide interacting 1 | -0,333458 | 2,07703 | -2,17946 | 0 |  |
| 207968_s_at | MEF2C | MADS box transcription enhancer factor 2, polypeptide C (myocyte enhancer factor 2C) | 0,8814099 | 0,86386 | -2,18158 | 0,60662 |  |
| 215501_s_at | DUSP10 | dual specificity phosphatase 10 | 0,9776398 | 0,77098 | -2,18578 | 0,33143 |  |
| 207165_at | HMMR | hyaluronan-mediated motility receptor (RHAMM) | 0,8603271 | 0,88898 | -2,18664 | 1,06427 |  |
| 1558253_x_at | ZNF587 | zinc finger protein 587 | 1,5416893 | 0,21123 | -2,19115 | 0,33143 |  |
| 201686_x_at | API5 | apoptosis inhibitor 5 | 1,0164528 | 0,73791 | -2,19295 | 0,82259 |  |
| 202089_s_at | SLC39A6 | solute carrier family 39 (zinc transporter), member 6 | 0,4470959 | 1,30927 | -2,19546 | 0,06315 |  |
| 205729_at | OSMR | oncostatin M receptor | 1,3913005 | 0,3696 | -2,20112 | 0,06315 |  |
| 206788_s_at | CBFB | core-binding factor, beta subunit | 0,9709224 | 0,79097 | -2,20237 | 0,16318 |  |
| 1557385_at | FLJ13305 | hypothetical protein FLJ13305 | 0,5152792 | 1,24945 | -2,20591 | 0,25444 |  |
| 210716_s_at | RSN | restin (Reed-Steinberg cell-expressed intermediate filament-associated protein) | 0,8099822 | 0,95657 | -2,20819 | 0,11715 |  |
| 216449_x_at | HSP90B1 | heat shock protein 90kDa beta (Grp94), member 1 | 1,5917394 | 0,18137 | -2,21638 | 0 |  |
| 215747_s_at | RCC1 /// SNHG3-RCC1 | regulator of chromosome condensation 1 /// regulator of chromosome condensation 1 | 1,1577619 | 0,61763 | -2,21924 | 2,53333 |  |
| 227940_at | LOC339803 | Hypothetical protein LOC339803 | 0,0883025 | 1,68828 | -2,22073 | 0,06315 |  |
| 214543_x_at | QKI | quaking homolog, KH domain RNA binding (mouse) | 1,0871926 | 0,68945 | -2,2208 | 0 |  |
| 209424_s_at | AMACR | alpha-methylacyl-CoA racemase | 0,8796364 | 0,90109 | -2,22591 | 0,43903 |  |
| 211478_s_at | DPP4 | dipeptidyl-peptidase 4 (CD26, adenosine deaminase complexing protein 2) | 0,3920727 | 1,39035 | -2,22803 | 0,06315 |  |
| 222067_x_at | HIST1H2BD | histone cluster 1, H2bd | 0,4678334 | 1,31522 | -2,22881 | 0 |  |
| 238695_s_at | RAB39B | RAB39B, member RAS oncogene family | 0,8001025 | 0,98328 | -2,22923 | 0,25444 |  |
| 201718_s_at | EPB41L2 | erythrocyte membrane protein band 4.1-like 2 | 0,2186844 | 1,56577 | -2,23057 | 0 |  |
| 238444_at | ZNF618 | zinc finger protein 618 | 0,4483038 | 1,33638 | -2,23085 | 0,25444 |  |
| 238089_at | MAN2C1 | mannosidase, alpha, class 2C, member 1 | 0,1936882 | 1,5914 | -2,23136 | 0,25444 |  |
| 205717_x_at | PCDHGC3 /// PCDHGB4 /// PCDHGA8 /// PCDHGA12 /// PCDHGC5 /// PCDHGC4 /// PCDHGB7 /// PCDHGB6 /// PCDHGB5 /// PCDHGB3 /// PCDHGB2 /// PCDHGB1 /// PCDHGA11 /// PCDHGA10 /// PCDHGA9 /// PCDHGA7 /// PCDHGA6 /// PCDHGA5 /// PCDHGA4 /// PCDHGA3 /// PCDHGA2 /// | protocadherin gamma subfamily C, 3 /// protocadherin gamma subfamily B, 4 /// protocadherin gamma subfamily A, 8 /// protocadherin gamma subfamily A, 12 /// protocadherin gamma subfamily C, 5 /// protocadherin gamma subfamily C, 4 /// protocadherin gamma | 0,165642 | 1,62405 | -2,23711 | 0,06315 |  |
| 213186_at | DZIP3 | zinc finger DAZ interacting protein 3 | 0,8844727 | 0,91172 | -2,24524 | 0,60662 |  |
| 223113_at | TMEM138 | transmembrane protein 138 | 0,7030788 | 1,09334 | -2,24553 | 0 |  |
| 213748_at | TRIM66 | tripartite motif-containing 66 | 0,8125332 | 0,98421 | -2,24593 | 0,82259 |  |
| 1553192_at | ZNF441 | zinc finger protein 441 | 0,7529823 | 1,04379 | -2,24597 | 1,90223 |  |
| 61734_at | RCN3 | reticulocalbin 3, EF-hand calcium binding domain | 1,727348 | 0,07053 | -2,24735 | 0 |  |
| 210622_x_at | CDK10 | cyclin-dependent kinase (CDC2-like) 10 | 1,4578936 | 0,34018 | -2,24759 | 0,33143 |  |
| 219380_x_at | POLH | polymerase (DNA directed), eta | 1,1159462 | 0,68372 | -2,24958 | 0,82259 |  |
| 210993_s_at | SMAD1 | SMAD family member 1 | 0,8156407 | 0,98883 | -2,25558 | 0,33143 |  |
| 223241_at | SNX8 | sorting nexin 8 | 1,0584499 | 0,74757 | -2,25753 | 0,82259 |  |
| 235388_at | CHD9 | chromodomain helicase DNA binding protein 9 | 0,6708934 | 1,13523 | -2,25766 | 0,25444 |  |
| 227709_at | LOC728913 | Similar to Reticulocalbin-1 precursor | 0,4605868 | 1,34663 | -2,25902 | 0,43903 |  |
| 1555831_s_at | LRRC41 | leucine rich repeat containing 41 | 0,4903292 | 1,31717 | -2,25938 | 0,11715 |  |
| 218402_s_at | HPS4 | Hermansky-Pudlak syndrome 4 | 0,689392 | 1,11924 | -2,26079 | 0,60662 |  |
| 204357_s_at | LIMK1 | LIM domain kinase 1 | 0,6993802 | 1,11439 | -2,26722 | 0,82259 |  |
| 203147_s_at | TRIM14 | tripartite motif-containing 14 | 0,9414711 | 0,87335 | -2,26852 | 0,06315 |  |
| 205140_at | FPGT | fucose-1-phosphate guanylyltransferase | 1,2582062 | 0,55686 | -2,26883 | 0,43903 |  |
| 213038_at | IBRDC3 | IBR domain containing 3 | 0,9229695 | 0,89264 | -2,26951 | 0,06315 |  |
| 216994_s_at | RUNX2 | runt-related transcription factor 2 | 1,0101154 | 0,80925 | -2,27421 | 0,16318 |  |
| 209030_s_at | IGSF4 | immunoglobulin superfamily, member 4 | 0,4502148 | 1,36937 | -2,27449 | 0 |  |
| 213529_at | ZNF688 | zinc finger protein 688 | 0,0426076 | 1,77992 | -2,27816 | 0,33143 |  |
| 218748_s_at | EXOC5 | exocyst complex component 5 | 1,026455 | 0,79726 | -2,27965 | 0,82259 |  |
| 209953_s_at | CDC37 | cell division cycle 37 homolog (S. cerevisiae) | 1,1083301 | 0,71697 | -2,28163 | 0,06315 |  |
| 221484_at | B4GALT5 | UDP-Gal:betaGlcNAc beta 1,4- galactosyltransferase, polypeptide 5 | 2,6672906 | -0,84064 | -2,28332 | 0 |  |
| 215561_s_at | IL1R1 | interleukin 1 receptor, type I | 1,190692 | 0,63757 | -2,28532 | 0,11715 |  |
| 235060_at | DKFZp547E087 | hypothetical gene LOC283846 | 1,5778802 | 0,2521 | -2,28747 | 0 |  |
| 205807_s_at | TUFT1 | tuftelin 1 | 0,614356 | 1,21687 | -2,28904 | 0,06315 |  |
| 222714_s_at | LACTB2 | lactamase, beta 2 | 0,5588807 | 1,27283 | -2,28963 | 0,11715 |  |
| 208868_s_at | GABARAPL1 | GABA(A) receptor-associated protein like 1 | 0,4967759 | 1,33767 | -2,29305 | 0,06315 |  |
| 207563_s_at | OGT | O-linked N-acetylglucosamine (GlcNAc) transferase (UDP-N-acetylglucosamine:polypeptide-N-acetylglucosaminyl transferase) | 0,9634672 | 0,87111 | -2,29322 | 0,16318 |  |
| 221679_s_at | ABHD6 | abhydrolase domain containing 6 | 0,8860536 | 0,94951 | -2,29446 | 0,43903 |  |
| 222102_at | GSTA3 | glutathione S-transferase A3 | 0,9424274 | 0,8938 | -2,29528 | 0 |  |
| 206306_at | RYR3 | ryanodine receptor 3 | 0,2016917 | 1,63469 | -2,29547 | 0 |  |
| 224792_at | TNKS1BP1 | tankyrase 1 binding protein 1, 182kDa | 1,2748475 | 0,56523 | -2,3001 | 0,33143 |  |
| 201746_at | TP53 | tumor protein p53 (Li-Fraumeni syndrome) | 0,952014 | 0,88878 | -2,30099 | 0,25444 |  |
| 204445_s_at | ALOX5 | arachidonate 5-lipoxygenase | -0,125347 | 1,96688 | -2,30191 | 0,06315 |  |
| 229257_at | KIAA1856 | KIAA1856 protein | 0,1983857 | 1,64482 | -2,30401 | 0,25444 |  |
| 225899_x_at | FLJ45445 /// FLJ45340 /// LOC653340 /// LOC727755 /// LOC728105 /// LOC728797 /// LOC729660 | FLJ45445 protein /// hypothetical gene supported by AK127273 /// hypothetical LOC653340 /// similar to FLJ45445 protein /// similar to FLJ45445 protein /// hypothetical protein LOC728797 /// hypothetical protein LOC729660 | 1,1757083 | 0,6689 | -2,30576 | 0,11715 |  |
| 34478_at | RAB11B | RAB11B, member RAS oncogene family | 0,8136031 | 1,03303 | -2,30829 | 1,43745 |  |
| 206085_s_at | CTH | cystathionase (cystathionine gamma-lyase) | 0,2183641 | 1,62929 | -2,30957 | 0 |  |
| 201279_s_at | DAB2 | disabled homolog 2, mitogen-responsive phosphoprotein (Drosophila) | 0,130886 | 1,71682 | -2,30963 | 0 |  |
| 201348_at | GPX3 | glutathione peroxidase 3 (plasma) | 1,423534 | 0,42875 | -2,31535 | 0 |  |
| 217297_s_at | MYO9B | myosin IXB | 1,0784408 | 0,77595 | -2,31799 | 0,25444 |  |
| 210543_s_at | PRKDC | protein kinase, DNA-activated, catalytic polypeptide | 1,2326593 | 0,62337 | -2,32004 | 0,11715 |  |
| 204338_s_at | RGS4 | regulator of G-protein signalling 4 | 1,2778744 | 0,5782 | -2,3201 | 0 |  |
| 231822_at | CTTNBP2NL | CTTNBP2 N-terminal like | 0,1646541 | 1,69161 | -2,32033 | 0,16318 |  |
| 226537_at | HINT3 | histidine triad nucleotide binding protein 3 | 0,4797301 | 1,37663 | -2,32046 | 0,60662 |  |
| 205961_s_at | PSIP1 | PC4 and SFRS1 interacting protein 1 | 0,955824 | 0,90381 | -2,32454 | 0,06315 |  |
| 210180_s_at | SFRS10 | splicing factor, arginine/serine-rich 10 (transformer 2 homolog, Drosophila) | 0,7068913 | 1,15396 | -2,32607 | 0,33143 |  |
| 228790_at | C8orf72 | chromosome 8 open reading frame 72 | 0,3225854 | 1,5384 | -2,32623 | 0 |  |
| 217799_x_at | UBE2H | ubiquitin-conjugating enzyme E2H (UBC8 homolog, yeast) | 0,203271 | 1,66197 | -2,33155 | 0 |  |
| 216627_s_at | B4GALT1 | UDP-Gal:betaGlcNAc beta 1,4- galactosyltransferase, polypeptide 1 | 1,50916 | 0,35796 | -2,3339 | 0,11715 |  |
| 1554193_s_at | MANEA | mannosidase, endo-alpha | 0,9978988 | 0,87555 | -2,34181 | 3,25444 |  |
| 216985_s_at | STX3 | syntaxin 3 | 0,2953806 | 1,57815 | -2,34192 | 0,16318 |  |
| 1558214_s_at | CTNNA1 | catenin (cadherin-associated protein), alpha 1, 102kDa | 1,5261272 | 0,34761 | -2,34217 | 0 |  |
| 1557137_at | TMEM17 | transmembrane protein 17 | 1,0948469 | 0,77983 | -2,34335 | 0,06315 |  |
| 1555736_a_at | AGTRAP | angiotensin II receptor-associated protein | 0,740687 | 1,13416 | -2,34356 | 0,16318 |  |
| 211810_s_at | GALC | galactosylceramidase | 0,6193451 | 1,25632 | -2,34458 | 0,11715 |  |
| 207622_s_at | ABCF2 | ATP-binding cassette, sub-family F (GCN20), member 2 | 1,102887 | 0,77386 | -2,34594 | 0,11715 |  |
| 211228_s_at | RAD17 | RAD17 homolog (S. pombe) | 0,726528 | 1,1527 | -2,34904 | 0,16318 |  |
| 201367_s_at | ZFP36L2 | zinc finger protein 36, C3H type-like 2 | 0,6676643 | 1,2127 | -2,35046 | 0,33143 |  |
| 225751_at | RBM17 | RNA binding motif protein 17 | 1,1750615 | 0,70614 | -2,3515 | 0,06315 |  |
| 203117_s_at | USP52 | ubiquitin specific peptidase 52 | 0,2615779 | 1,62014 | -2,35215 | 0,11715 |  |
| 216506_x_at | MLLT10 | myeloid/lymphoid or mixed-lineage leukemia (trithorax homolog, Drosophila); translocated to, 10 | 0,9987603 | 0,88566 | -2,35553 | 0,16318 |  |
| 210753_s_at | EPHB1 | EPH receptor B1 | -0,34569 | 2,23415 | -2,36057 | 0 |  |
| 205047_s_at | ASNS | asparagine synthetase | 0,7349931 | 1,15426 | -2,36156 | 0 |  |
| 235362_at | LOC729970 | Transcribed locus /// Hypothetical protein LOC729970 | 0,6879368 | 1,20319 | -2,36391 | 0,06315 |  |
| 227260_at | ANKRD10 | Ankyrin repeat domain 10 | 0,2829206 | 1,60856 | -2,36436 | 0,82259 |  |
| 211110_s_at | AR | androgen receptor (dihydrotestosterone receptor; testicular feminization; spinal and bulbar muscular atrophy; Kennedy disease) | 0,9914746 | 0,90084 | -2,36539 | 0,25444 |  |
| 231259_s_at | Cyclin D2 | Transcribed locus | 0,3785096 | 1,51677 | -2,3691 | 0,16318 |  |
| 209041_s_at | UBE2G2 | ubiquitin-conjugating enzyme E2G 2 (UBC7 homolog, yeast) | 0,8472899 | 1,05085 | -2,37267 | 0,11715 |  |
| 217234_s_at | VIL2 | villin 2 (ezrin) | 1,3247259 | 0,57439 | -2,3739 | 0 |  |
| 201476_s_at | RRM1 | ribonucleotide reductase M1 polypeptide | 1,3973154 | 0,50389 | -2,3765 | 0,25444 |  |
| 202898_at | SDC3 | syndecan 3 (N-syndecan) | 0,8740612 | 1,03111 | -2,38147 | 0 |  |
| 1557081_at | RBM25 | RNA binding motif protein 25 | 1,0939552 | 0,81332 | -2,3841 | 0,16318 |  |
| 225876_at | NPAL3 | NIPA-like domain containing 3 | 0,615881 | 1,29157 | -2,38432 | 0,06315 |  |
| 222955_s_at | FAM45B /// FAM45A /// LOC731832 | family with sequence similarity 45, member B /// family with sequence similarity 45, member A /// similar to family with sequence similarity 45, member A | 0,810895 | 1,09969 | -2,38824 | 0,11715 |  |
| 209921_at | SLC7A11 | solute carrier family 7, (cationic amino acid transporter, y+ system) member 11 | 0,3748906 | 1,53588 | -2,38846 | 0 |  |
| 1553710_at | FLJ31659 | hypothetical protein FLJ31659 | 0,0325386 | 1,87834 | -2,3886 | 0 |  |
| 210764_s_at | CYR61 | cysteine-rich, angiogenic inducer, 61 | 0,5547621 | 1,3613 | -2,39508 | 0 |  |
| 222719_s_at | PDGFC | platelet derived growth factor C | 0,2839533 | 1,63583 | -2,39972 | 0 |  |
| 218775_s_at | WWC2 | WW and C2 domain containing 2 | 1,0471747 | 0,8728 | -2,39997 | 0,06315 |  |
| 204479_at | OSTF1 | osteoclast stimulating factor 1 | 0,7765477 | 1,14599 | -2,40317 | 0,16318 |  |
| 208791_at | CLU | clusterin | 0,5024347 | 1,42212 | -2,40569 | 0 |  |
| 201247_at | SREBP2 precursor = SREBP2 (Golgi membrane) | Sterol regulatory element binding transcription factor 2 | 0,8001619 | 1,12521 | -2,40672 | 0 |  |
| 1562228_s_at | PDE5A | phosphodiesterase 5A, cGMP-specific | 0,8807296 | 1,05163 | -2,41545 | 0,25444 |  |
| 205457_at | C6orf106 | chromosome 6 open reading frame 106 | 1,2633684 | 0,67025 | -2,41703 | 0,06315 |  |
| 218051_s_at | NT5DC2 | 5'-nucleotidase domain containing 2 | 0,6890426 | 1,24635 | -2,41925 | 0 |  |
| 1559954_s_at | DDX42 | DEAD (Asp-Glu-Ala-Asp) box polypeptide 42 | 1,4261401 | 0,51534 | -2,42686 | 0,33143 |  |
| 210513_s_at | VEGFA | vascular endothelial growth factor A | 0,3594686 | 1,58243 | -2,42737 | 0,11715 |  |
| 1554119_at | C16orf57 | chromosome 16 open reading frame 57 | -0,95092 | 2,89327 | -2,42794 | 0 |  |
| 220240_s_at | TMCO3 | transmembrane and coiled-coil domains 3 | 0,1104471 | 1,83202 | -2,42808 | 0 |  |
| 201559_s_at | CLIC4 | chloride intracellular channel 4 | 0,8678248 | 1,08343 | -2,43906 | 0 |  |
| 241709_s_at | DOCK1 | dedicator of cytokinesis 1 | 0,8615496 | 1,08981 | -2,4392 | 0,43903 |  |
| 213986_s_at | C19orf6 | chromosome 19 open reading frame 6 | 0,7710131 | 1,18205 | -2,44133 | 0,33143 |  |
| 227159_at | GHDC | GH3 domain containing | 0,8522231 | 1,1012 | -2,44178 | 0,25444 |  |
| 204589_at | NUAK1 | NUAK family, SNF1-like kinase, 1 | 0,532546 | 1,42159 | -2,44267 | 0 |  |
| 220532_s_at | TMEM176B | transmembrane protein 176B | 0,4071565 | 1,54806 | -2,44403 | 0 |  |
| 228948_at | EPHA4 | EPH receptor A4 | -0,061703 | 2,01741 | -2,44463 | 0 |  |
| 203198_at | CDK9 | cyclin-dependent kinase 9 (CDC2-related kinase) | 0,5876671 | 1,36999 | -2,44708 | 0,06315 |  |
| 202345_s_at | FABP5 /// LOC728641 /// LOC729163 /// LOC731043 /// LOC732031 | fatty acid binding protein 5 (psoriasis-associated) /// similar to Fatty acid-binding protein, epidermal (E-FABP) (Psoriasis-associated fatty acid-binding protein homolog) (PA-FABP) /// similar to Fatty acid-binding protein, epidermal (E-FABP) (Psoriasis- | 0,5450049 | 1,41376 | -2,44845 | 0 |  |
| 1554821_a_at | ZBED1 | zinc finger, BED-type containing 1 | 1,046594 | 0,91444 | -2,45129 | 0 |  |
| 202847_at | PCK2 | phosphoenolpyruvate carboxykinase 2 (mitochondrial) | 0,8171119 | 1,15045 | -2,45946 | 0 |  |
| 214701_s_at | FN1 | fibronectin 1 | 1,4221319 | 0,5457 | -2,45979 | 0 |  |
| 216048_s_at | RHOBTB3 | Rho-related BTB domain containing 3 | 0,6461227 | 1,3228 | -2,46116 | 0 |  |
| 237464_at | IMAA | SLC7A5 pseudogene | 1,11364 | 0,85624 | -2,46235 | 0,16318 |  |
| 224443_at | C1orf97 | chromosome 1 open reading frame 97 /// chromosome 1 open reading frame 97 | 1,3474861 | 0,62346 | -2,46368 | 0 |  |
| 238010_at | C1orf174 | chromosome 1 open reading frame 174 | 1,1222067 | 0,85672 | -2,47366 | 0,33143 |  |
| 202145_at | LY6E | lymphocyte antigen 6 complex, locus E | 2,2585511 | -0,27816 | -2,47549 | 0 |  |
| 202161_at | PKN1 | protein kinase N1 | 1,030724 | 0,9517 | -2,47803 | 0,11715 |  |
| 201835_s_at | PRKAB1 | protein kinase, AMP-activated, beta 1 non-catalytic subunit | 0,6518409 | 1,33193 | -2,47971 | 0,16318 |  |
| 37965_at | PARVB | parvin, beta | 0,7524121 | 1,2315 | -2,4799 | 0,33143 |  |
| 223321_s_at | FGFRL1 | fibroblast growth factor receptor-like 1 | 1,0170549 | 0,97051 | -2,48446 | 0,06315 |  |
| 1552622_s_at | POLR2J2 /// LOC441259 /// LOC730323 | DNA directed RNA polymerase II polypeptide J-related gene /// similar to postmeiotic segregation increased 2-like 2 /// similar to postmeiotic segregation increased 2-like 2 | 1,5726001 | 0,41665 | -2,48656 | 0,25444 |  |
| 218540_at | THTPA | thiamine triphosphatase | 0,7862673 | 1,21057 | -2,49605 | 0,06315 |  |
| 207782_s_at | PSEN1 | presenilin 1 (Alzheimer disease 3) | 1,1061693 | 0,894 | -2,50021 | 0,06315 |  |
| 1553535_a_at | RANGAP1 | Ran GTPase activating protein 1 | 1,2755978 | 0,72659 | -2,50273 | 0,06315 |  |
| 226372_at | CHST11 | Carbohydrate (chondroitin 4) sulfotransferase 11 | 1,02365 | 0,98043 | -2,5051 | 0 |  |
| 205017_s_at | MBNL2 | muscleblind-like 2 (Drosophila) | 1,3873626 | 0,61999 | -2,50919 | 0,43903 |  |
| 211864_s_at | FER1L3 | fer-1-like 3, myoferlin (C. elegans) | 0,7740694 | 1,23614 | -2,51276 | 0 |  |
| 1561281_a_at | LOC284576 | hypothetical protein LOC284576 | 0,8598638 | 1,15198 | -2,51481 | 0,11715 |  |
| 65585_at | FAM86B1 | family with sequence similarity 86, member B1 | 0,1341958 | 1,88034 | -2,51817 | 0 |  |
| 230492_s_at | RP5-1022P6.2 | hypothetical protein KIAA1434 | 1,1441491 | 0,87452 | -2,52333 | 0,06315 |  |
| 1555467_a_at | CUGBP1 | CUG triplet repeat, RNA binding protein 1 | 0,6458462 | 1,37342 | -2,52408 | 0,11715 |  |
| 223798_at | SLC41A2 | solute carrier family 41, member 2 | 1,1319695 | 0,89003 | -2,5275 | 0,16318 |  |
| 1569311_at | LOC554203 | Hypothetical LOC554203 | 0,8423274 | 1,18045 | -2,52848 | 1,06427 |  |
| 1558044_s_at | EXOSC6 | Exosome component 6 | 0,87174 | 1,15143 | -2,52896 | 0 |  |
| 208523_x_at | HIST1H2BI | histone cluster 1, H2bi | 0,8126987 | 1,21557 | -2,53534 | 0,06315 |  |
| 244287_at | SFRS12 | splicing factor, arginine/serine-rich 12 | 1,0207464 | 1,01172 | -2,54058 | 0 |  |
| 220454_s_at | SEMA6A | sema domain, transmembrane domain (TM), and cytoplasmic domain, (semaphorin) 6A | 1,1372703 | 0,89802 | -2,54412 | 0 |  |
| 213562_s_at | SQLE | squalene epoxidase | 1,1908411 | 0,84732 | -2,5477 | 0 |  |
| 226173_at | OATL1 | ornithine aminotransferase-like 1 | 0,5633194 | 1,47529 | -2,54826 | 0 |  |
| 208442_s_at | ATM /// LOC651610 | ataxia telangiectasia mutated (includes complementation groups A, C and D) /// similar to Serine-protein kinase ATM (Ataxia telangiectasia mutated) (A-T, mutated) | 0,8949034 | 1,14547 | -2,55047 | 0,82259 |  |
| 202017_at | EPHX1 | epoxide hydrolase 1, microsomal (xenobiotic) | 1,3773607 | 0,66796 | -2,55665 | 0 |  |
| 202143_s_at | COPS8 | COP9 constitutive photomorphogenic homolog subunit 8 (Arabidopsis) | 0,2842961 | 1,76177 | -2,55758 | 0 |  |
| 200935_at | CALR | calreticulin | 0,7105936 | 1,33839 | -2,56124 | 0 |  |
| 225782_at | MSRB3 | methionine sulfoxide reductase B3 | 0,2244313 | 1,82905 | -2,56685 | 0 |  |
| 241343_at | RNASEH1 | Ribonuclease H1 | 1,3533187 | 0,70214 | -2,56932 | 0,16318 |  |
| 214720_x_at | SEPT10 | septin 10 | 1,2089169 | 0,84808 | -2,57125 | 0 |  |
| 208490_x_at | HIST1H2BF | histone cluster 1, H2bf | 0,870837 | 1,19007 | -2,57614 | 0 |  |
| 215434_x_at | NBPF1 /// NBPF10 | neuroblastoma breakpoint family, member 1 /// neuroblastoma breakpoint family, member 10 | 0,6806635 | 1,38252 | -2,57898 | 0 |  |
| 205746_s_at | ADAM17 | ADAM metallopeptidase domain 17 (tumor necrosis factor, alpha, converting enzyme) | 1,4426931 | 0,6211 | -2,57974 | 0 |  |
| 225454_at | CCDC124 | coiled-coil domain containing 124 | 0,66776 | 1,40106 | -2,58603 | 0 |  |
| 213937_s_at | FTSJ1 | FtsJ homolog 1 (E. coli) | 1,3550173 | 0,7146 | -2,58702 | 0 |  |
| 203032_s_at | FH | fumarate hydratase | 1,4063671 | 0,66329 | -2,58707 | 0,11715 |  |
| 201668_x_at | MARCKS | myristoylated alanine-rich protein kinase C substrate | 1,2750015 | 0,79521 | -2,58776 | 0 |  |
| 217216_x_at | MLH3 | mutL homolog 3 (E. coli) | 1,1287833 | 0,94173 | -2,58814 | 0,06315 |  |
| 209398_at | HIST1H1C | histone cluster 1, H1c | -0,146706 | 2,22239 | -2,59461 | 0 |  |
| 207064_s_at | AOC2 | amine oxidase, copper containing 2 (retina-specific) | 1,5205414 | 0,55568 | -2,59528 | 0 |  |
| 203425_s_at | IGFBP5 | insulin-like growth factor binding protein 5 | 0,735626 | 1,34131 | -2,59617 | 0 |  |
| 211985_s_at | CALM1 | calmodulin 1 (phosphorylase kinase, delta) | 0,7800518 | 1,29796 | -2,59752 | 0 |  |
| 226809_at | FLJ30428 /// LOC730024 | similar to hypothetical protein A230046P18; cDNA sequence BC055759 /// similar to male sterility domain containing 1 | 0,1622117 | 1,91675 | -2,5987 | 0,06315 |  |
| 218045_x_at | PTMS | parathymosin | 1,2565194 | 0,82785 | -2,60547 | 0 |  |
| 225727_at | PLEKHH1 | pleckstrin homology domain containing, family H (with MyTH4 domain) member 1 | 1,2346535 | 0,8537 | -2,61044 | 0 |  |
| 214336_s_at | COPA | coatomer protein complex, subunit alpha | 2,1068804 | -0,01829 | -2,61074 | 0 |  |
| 224566_at | TncRNA | trophoblast-derived noncoding RNA | 1,0720398 | 1,01787 | -2,61238 | 0 |  |
| 228746_s_at | CDV3 | CDV3 homolog (mouse) | -0,170044 | 2,26144 | -2,61425 | 0 |  |
| 204881_s_at | UGCG | UDP-glucose ceramide glucosyltransferase | 1,0701601 | 1,02268 | -2,61605 | 0 |  |
| 201101_s_at | BCLAF1 | BCL2-associated transcription factor 1 | 0,6928102 | 1,41646 | -2,63658 | 0 |  |
| 218367_x_at | USP21 | ubiquitin specific peptidase 21 | 0,8627702 | 1,2479 | -2,63833 | 0,06315 |  |
| 204418_x_at | GSTM2 | glutathione S-transferase M2 (muscle) | 0,0638274 | 2,04898 | -2,641 | 0 |  |
| 221194_s_at | LOC51136 | PTD016 protein | 0,7272628 | 1,38632 | -2,64198 | 0 |  |
| 210387_at | HIST1H2BG | histone cluster 1, H2bg | 1,2184744 | 0,89571 | -2,64273 | 0 |  |
| 1558733_at | ZBTB38 | zinc finger and BTB domain containing 38 | 1,1618918 | 0,95496 | -2,64606 | 0 |  |
| 213865_at | DCBLD2 | discoidin, CUB and LCCL domain containing 2 | -0,009066 | 2,128 | -2,64867 | 0 |  |
| 1555740_a_at | MRAP | melanocortin 2 receptor accessory protein | 0,2538045 | 1,87273 | -2,65817 | 0 |  |
| 209113_s_at | HMG20B | high-mobility group 20B | 1,4364904 | 0,69191 | -2,66051 | 0 |  |
| 1556067_a_at | JMJD3 | jumonji domain containing 3 | 0,9321565 | 1,1971 | -2,66157 | 0 |  |
| 216064_s_at | AGA | aspartylglucosaminidase | 1,50379 | 0,62831 | -2,66513 | 0,11715 |  |
| 219326_s_at | B3GNT2 | UDP-GlcNAc:betaGal beta-1,3-N-acetylglucosaminyltransferase 2 | 0,4872365 | 1,64494 | -2,66522 | 0 |  |
| 224410_s_at | LMBR1 | limb region 1 homolog (mouse) /// limb region 1 homolog (mouse) | 1,3638218 | 0,77076 | -2,66823 | 0 |  |
| 219878_s_at | KLF13 | Kruppel-like factor 13 | 1,0454717 | 1,09152 | -2,67125 | 0 |  |
| 229411_at | PNCK | pregnancy upregulated non-ubiquitously expressed CaM kinase | 2,2981219 | -0,15446 | -2,67958 | 0 |  |
| 1555240_s_at | GNG12 | guanine nucleotide binding protein (G protein), gamma 12 | 1,0126805 | 1,13107 | -2,67968 | 0 |  |
| 1555830_s_at | FAM62B | family with sequence similarity 62 (C2 domain containing) member B | 1,7906435 | 0,35455 | -2,6815 | 0 |  |
| 219932_at | SLC27A6 | solute carrier family 27 (fatty acid transporter), member 6 | 1,2804234 | 0,86792 | -2,68543 | 0,06315 |  |
| 223971_at | OR2A20P /// OR2A9P /// LOC730421 | olfactory receptor, family 2, subfamily A, member 20 pseudogene /// olfactory receptor, family 2, subfamily A, member 9 pseudogene /// region containing olfactory receptor, family 2, subfamily A, member 9 pseudogene; olfactory receptor, family 2, subfamil | 0,6816629 | 1,46913 | -2,68849 | 0,43903 |  |
| 201120_s_at | PGRMC1 | progesterone receptor membrane component 1 | 1,8891277 | 0,26597 | -2,69387 | 0 |  |
| 1557455_s_at | MOSPD1 | motile sperm domain containing 1 | 0,4199799 | 1,74247 | -2,70306 | 0 |  |
| 1555326_a_at | ADAM9 | ADAM metallopeptidase domain 9 (meltrin gamma) | 0,4842633 | 1,67823 | -2,70312 | 0 |  |
| 235014_at | LOC147727 | Hypothetical protein LOC147727 | 0,3482083 | 1,82761 | -2,71977 | 0 |  |
| 215749_s_at | GORASP1 | golgi reassembly stacking protein 1, 65kDa | 0,3243939 | 1,85872 | -2,72889 | 0,06315 |  |
| 209105_at | NCOA1 | nuclear receptor coactivator 1 | 0,9725544 | 1,21124 | -2,72975 | 0 |  |
| 1553218_a_at | ZNF512 | zinc finger protein 512 | 0,4429365 | 1,74112 | -2,73007 | 0 |  |
| 210974_s_at | AP3D1 | adaptor-related protein complex 3, delta 1 subunit | 0,9817173 | 1,20265 | -2,73046 | 0 |  |
| 214649_s_at | MTMR2 | myotubularin related protein 2 | -0,009259 | 2,19382 | -2,7307 | 0 |  |
| 1552627_a_at | ARHGAP5 | Rho GTPase activating protein 5 | 1,0926818 | 1,09298 | -2,73207 | 0 |  |
| 204925_at | CTNS | cystinosis, nephropathic | 0,8436097 | 1,34638 | -2,73749 | 0 |  |
| 221881_s_at | ADD3 /// CLIC4 | adducin 3 (gamma) /// chloride intracellular channel 4 | 1,4604807 | 0,73528 | -2,7447 | 0 |  |
| 227406_at | GABPB2 | GA binding protein transcription factor, beta subunit 2 | 1,2601434 | 0,93725 | -2,74674 | 0 |  |
| 221323_at | ULBP1 | UL16 binding protein 1 | 0,5723104 | 1,62748 | -2,74974 | 0 |  |
| 202755_s_at | GPC1 | glypican 1 | 0,6848815 | 1,51519 | -2,75009 | 0 |  |
| 229986_at | ZNF717 | zinc finger protein 717 | 1,3510679 | 0,85039 | -2,75182 | 0 |  |
| 238505_at | ADPRH | ADP-ribosylarginine hydrolase | 1,533731 | 0,67567 | -2,76175 | 0,06315 |  |
| 208874_x_at | PPP2R4 | protein phosphatase 2A, regulatory subunit B' (PR 53) | 0,9000526 | 1,30954 | -2,76198 | 0 |  |
| 211911_x_at | HLA-B | major histocompatibility complex, class I, B /// major histocompatibility complex, class I, B | 1,4751855 | 0,73819 | -2,76672 | 0 |  |
| 223242_s_at | ET | hypothetical protein ET | 0,6700575 | 1,54396 | -2,76752 | 0 |  |
| 218345_at | TMEM176A | transmembrane protein 176A | 0,1556862 | 2,06091 | -2,77074 | 0 |  |
| 208527_x_at | HIST1H2BE | histone cluster 1, H2be | 0,562909 | 1,65464 | -2,77194 | 0 |  |
| 208540_x_at | LOC729659 /// LOC730278 /// LOC730558 | similar to Putative S100 calcium-binding protein A11 pseudogene /// similar to Putative S100 calcium-binding protein A11 pseudogene /// similar to Putative S100 calcium-binding protein A11 pseudogene | 2,0107881 | 0,20751 | -2,77288 | 0 |  |
| 223635_s_at | SSBP3 /// IL17RB | single stranded DNA binding protein 3 /// interleukin 17 receptor B | 0,7546926 | 1,46416 | -2,77356 | 0,06315 |  |
| 218992_at | C9orf46 | chromosome 9 open reading frame 46 | 1,0580056 | 1,16124 | -2,77406 | 0 |  |
| 217729_s_at | AES | amino-terminal enhancer of split | 0,8438516 | 1,37557 | -2,77428 | 0 |  |
| 220892_s_at | PSAT1 | phosphoserine aminotransferase 1 | 0,3272452 | 1,89612 | -2,77921 | 0 |  |
| 227446_s_at | C14orf167 | chromosome 14 open reading frame 167 | 0,2127208 | 2,01183 | -2,78069 | 0 |  |
| 200722_s_at | GPIAP1 | GPI-anchored membrane protein 1 | 1,2410281 | 0,98514 | -2,78271 | 0 |  |
| 206090_s_at | DISC1 | disrupted in schizophrenia 1 | 0,6943913 | 1,54274 | -2,79641 | 0 |  |
| 217903_at | STRN4 | striatin, calmodulin binding protein 4 | 0,646345 | 1,59231 | -2,79832 | 0 |  |
| 239536_at | C1orf56 | Chromosome 1 open reading frame 56 | 1,4457167 | 0,79398 | -2,79962 | 0,16318 |  |
| 216593_s_at | PIGC | phosphatidylinositol glycan anchor biosynthesis, class C | 0,6208856 | 1,61921 | -2,80012 | 0 |  |
| 201044_x_at | DUSP1 | dual specificity phosphatase 1 | 0,4763864 | 1,765 | -2,80173 | 0 |  |
| 206099_at | PRKCH | protein kinase C, eta | 1,2838279 | 0,95796 | -2,80223 | 0 |  |
| 201337_s_at | VAMP3 | vesicle-associated membrane protein 3 (cellubrevin) | 1,3663872 | 0,87574 | -2,80266 | 0 |  |
| 215253_s_at | DSCR1 | Down syndrome critical region gene 1 | -0,013247 | 2,25978 | -2,80816 | 0 |  |
| 216942_s_at | CD58 | CD58 molecule | 0,81726 | 1,43287 | -2,81266 | 0 |  |
| 222590_s_at | NLK | nemo-like kinase | 1,2228359 | 1,02825 | -2,81386 | 0 |  |
| 200617_at | KIAA0152 | KIAA0152 | 1,1049359 | 1,14757 | -2,81564 | 0 |  |
| 222699_s_at | PLEKHF2 | pleckstrin homology domain containing, family F (with FYVE domain) member 2 | 0,424164 | 1,82995 | -2,81764 | 0 |  |
| 204864_s_at | IL6ST | interleukin 6 signal transducer (gp130, oncostatin M receptor) | 1,4972809 | 0,76861 | -2,83237 | 0 |  |
| 214895_s_at | ADAM10 | ADAM metallopeptidase domain 10 | 1,6828133 | 0,58577 | -2,83572 | 0 |  |
| 219599_at | PRO1843 | hypothetical protein PRO1843 | 0,7178442 | 1,55568 | -2,84191 | 0 |  |
| 225160_x_at | MGC5370 | hypothetical protein MGC5370 | 0,2719068 | 2,00369 | -2,8445 | 0 |  |
| 201813_s_at | TBC1D5 | TBC1 domain family, member 5 | 1,0697746 | 1,20709 | -2,84608 | 0 |  |
| 1555526_a_at | Septin 6 | septin 6 | 1,178231 | 1,09952 | -2,84719 | 0 |  |
| 226306_at | C6orf1 | chromosome 6 open reading frame 1 | -0,272362 | 2,56974 | -2,87172 | 0 |  |
| 242477_at | C9orf52 | chromosome 9 open reading frame 52 | 0,8083418 | 1,49014 | -2,87311 | 0,16318 |  |
| 211255_x_at | DEDD | death effector domain containing | 0,6889673 | 1,61 | -2,87371 | 0 |  |
| 215333_x_at | GSTM1 | glutathione S-transferase M1 | 0,0473889 | 2,25937 | -2,88345 | 0 |  |
| 201026_at | EIF5B | eukaryotic translation initiation factor 5B | 1,1944817 | 1,11405 | -2,88567 | 0 |  |
| 211153_s_at | TNFSF11 | tumor necrosis factor (ligand) superfamily, member 11 | 0,8319093 | 1,4769 | -2,88601 | 0 |  |
| 224560_at | TIMP2 | TIMP metallopeptidase inhibitor 2 | 0,9807346 | 1,32942 | -2,8877 | 0 |  |
| 210707_x_at | PMS2L11 | postmeiotic segregation increased 2-like 11 | 0,6675569 | 1,64556 | -2,89139 | 0 |  |
| 231955_s_at | HIBADH | 3-hydroxyisobutyrate dehydrogenase | 1,963398 | 0,35792 | -2,90165 | 0 |  |
| 225622_at | PAG1 | phosphoprotein associated with glycosphingolipid microdomains 1 | 0,4510016 | 1,87691 | -2,90989 | 0 |  |
| 209582_s_at | CD200 | CD200 molecule | 1,3968438 | 0,93515 | -2,91499 | 0 |  |
| 211074_at | FOLR1 | folate receptor 1 (adult) /// folate receptor 1 (adult) | 1,3808159 | 0,96009 | -2,92613 | 0 |  |
| 209428_s_at | ZFPL1 | zinc finger protein-like 1 | 1,1349374 | 1,20893 | -2,92983 | 0 |  |
| 224823_at | MYLK | myosin, light chain kinase | 0,8372822 | 1,50666 | -2,92993 | 0 |  |
| 215236_s_at | PICALM | phosphatidylinositol binding clathrin assembly protein | 0,7462257 | 1,59925 | -2,93185 | 0 |  |
| 219927_at | C14orf111 | chromosome 14 open reading frame 111 | 1,3764218 | 0,9691 | -2,9319 | 0,11715 |  |
| 211085_s_at | STK4 | serine/threonine kinase 4 /// serine/threonine kinase 4 | 1,3344607 | 1,01716 | -2,93952 | 0 |  |
| 201308_s_at | Septin 11 | septin 11 | 1,3969909 | 0,96218 | -2,94896 | 0 |  |
| 202566_s_at | SVIL | supervillin | 1,3035866 | 1,05838 | -2,95246 | 0 |  |
| 204041_at | MAOB | monoamine oxidase B | 0,2676667 | 2,09647 | -2,95517 | 0 |  |
| 1569792_a_at | MGC50559 | hypothetical protein MGC50559 | 0,8094246 | 1,55633 | -2,95719 | 0,06315 |  |
| 210284_s_at | MAP3K7IP2 | mitogen-activated protein kinase kinase kinase 7 interacting protein 2 | 1,1317445 | 1,2374 | -2,96143 | 0 |  |
| 212337_at | TI-227H /// TUG1 | hypothetical protein TI-227H /// taurine upregulated gene 1 | 0,4677277 | 1,90535 | -2,96635 | 0 |  |
| 1558093_s_at | MATR3 | matrin 3 | 1,2752549 | 1,09993 | -2,96899 | 0 |  |
| 215113_s_at | SENP3 | SUMO1/sentrin/SMT3 specific peptidase 3 | 0,951955 | 1,42371 | -2,96959 | 0 |  |
| 213274_s_at | CTSB | cathepsin B | 1,827854 | 0,54974 | -2,97199 | 0 |  |
| 215195_at | PRKCA | protein kinase C, alpha | 0,8950385 | 1,48493 | -2,97496 | 0 |  |
| 207079_s_at | MED6 | mediator of RNA polymerase II transcription, subunit 6 homolog (S. cerevisiae) | 1,9388678 | 0,44777 | -2,98329 | 0 |  |
| 1558685_a_at | LOC158960 | hypothetical protein BC009467 | 0,882633 | 1,50472 | -2,98419 | 0 |  |
| 209622_at | STK16 | serine/threonine kinase 16 | 0,0724708 | 2,32746 | -2,99991 | 0 |  |
| 212292_at | SLC7A1 | solute carrier family 7 (cationic amino acid transporter, y+ system), member 1 | 1,3339623 | 1,06753 | -3,00186 | 0 |  |
| 209743_s_at | ITCH | itchy homolog E3 ubiquitin protein ligase (mouse) | 1,5176803 | 0,88698 | -3,00583 | 0 |  |
| 232079_s_at | PVRL2 | poliovirus receptor-related 2 (herpesvirus entry mediator B) | 1,4420054 | 0,96482 | -3,00853 | 0 |  |
| 220368_s_at | SMEK1 | SMEK homolog 1, suppressor of mek1 (Dictyostelium) | 2,0989486 | 0,31022 | -3,01146 | 0 |  |
| 201148_s_at | TIMP3 | TIMP metallopeptidase inhibitor 3 (Sorsby fundus dystrophy, pseudoinflammatory) | 0,2526068 | 2,15657 | -3,01147 | 0 |  |
| 201008_s_at | TXNIP | thioredoxin interacting protein | 1,2416767 | 1,16917 | -3,01355 | 0 |  |
| 238539_at | HPS3 | Hermansky-Pudlak syndrome 3 | 0,6943392 | 1,7228 | -3,02142 | 0 |  |
| 202354_s_at | GTF2F1 | general transcription factor IIF, polypeptide 1, 74kDa | 1,4227923 | 0,99583 | -3,02327 | 0 |  |
| 222458_s_at | C1orf108 | chromosome 1 open reading frame 108 | 1,2517883 | 1,17408 | -3,03233 | 0 |  |
| 211708_s_at | SCD | stearoyl-CoA desaturase (delta-9-desaturase) /// stearoyl-CoA desaturase (delta-9-desaturase) | 1,7043863 | 0,73057 | -3,0437 | 0 |  |
| 210105_s_at | FYN | FYN oncogene related to SRC, FGR, YES | -0,563374 | 3,0005 | -3,04641 | 0 |  |
| 221432_s_at | SLC25A28 | solute carrier family 25, member 28 /// solute carrier family 25, member 28 | 1,5554843 | 0,88257 | -3,04757 | 0 |  |
| 231952_at | LOC731450 | hypothetical protein LOC731450 | -0,129421 | 2,56985 | -3,05053 | 0 |  |
| 225399_at | C1orf19 | chromosome 1 open reading frame 19 | 0,9275724 | 1,51408 | -3,05207 | 0 |  |
| 200661_at | CTSA | cathepsin A | 0,3660414 | 2,07726 | -3,05413 | 0 |  |
| 200918_s_at | SRPR | signal recognition particle receptor ('docking protein') | 1,7531308 | 0,69487 | -3,06 | 0 |  |
| 224626_at | SLC35A4 | solute carrier family 35, member A4 | 1,2872573 | 1,16194 | -3,0615 | 0 |  |
| 1554553_s_at | YIF1B | Yip1 interacting factor homolog B (S. cerevisiae) | 1,6811344 | 0,76835 | -3,06186 | 0 |  |
| 200607_s_at | RAD21 | RAD21 homolog (S. pombe) | 1,2613619 | 1,19096 | -3,0654 | 0 |  |
| 228834_at | TOB1 | transducer of ERBB2, 1 | -0,280094 | 2,73335 | -3,06657 | 0 |  |
| 224632_at | GPATCH4 | G patch domain containing 4 | 1,3576497 | 1,09855 | -3,07025 | 0 |  |
| 206113_s_at | RAB5A | RAB5A, member RAS oncogene family | 1,1038352 | 1,35274 | -3,07072 | 0 |  |
| 223005_s_at | C9orf5 | chromosome 9 open reading frame 5 | 0,9403385 | 1,51766 | -3,0725 | 0 |  |
| 210541_s_at | TRIM27 | tripartite motif-containing 27 | 0,4314441 | 2,03332 | -3,08096 | 0 |  |
| 209035_at | MDK | midkine (neurite growth-promoting factor 2) | 0,9444872 | 1,52471 | -3,08649 | 0 |  |
| 224572_s_at | IRF2BP2 | interferon regulatory factor 2 binding protein 2 | 0,5682151 | 1,90115 | -3,08671 | 0 |  |
| 201109_s_at | THBS1 | thrombospondin 1 | 0,7095259 | 1,76176 | -3,0891 | 0 |  |
| 228346_at | FLJ14959 | Hypothetical protein FLJ14959 | 1,2783805 | 1,1972 | -3,09447 | 0 |  |
| 211968_s_at | HSP90AA1 | heat shock protein 90kDa alpha (cytosolic), class A member 1 | 1,3562865 | 1,12233 | -3,09827 | 0 |  |
| 227106_at | LOC440104 | similar to RIKEN cDNA 1110012D08 | 0,2956926 | 2,18892 | -3,10577 | 0 |  |
| 224982_at | AKT1S1 | AKT1 substrate 1 (proline-rich) | 1,9440684 | 0,54105 | -3,1064 | 0 |  |
| 225442_at | DDR2 | Discoidin domain receptor family, member 2 | -0,234924 | 2,72142 | -3,10812 | 0 |  |
| 209355_s_at | PPAP2B | phosphatidic acid phosphatase type 2B | 0,1106442 | 2,37688 | -3,10941 | 0 |  |
| 211924_s_at | PLAUR | plasminogen activator, urokinase receptor /// plasminogen activator, urokinase receptor | 0,2606763 | 2,23113 | -3,11476 | 0 |  |
| 1554958_at | ZNF641 | zinc finger protein 641 | 1,1805759 | 1,31978 | -3,12544 | 0 |  |
| 210178_x_at | FUSIP1 /// LOC727922 | FUS interacting protein (serine/arginine-rich) 1 /// similar to FUS-interacting serine-arginine-rich protein 1 (TLS-associated protein with Ser-Arg repeats) (TLS-associated protein with SR repeats) (TASR) (TLS-associated serine-arginine protein) (TLS-asso | 1,3413114 | 1,16788 | -3,13648 | 0 |  |
| 218903_s_at | OBFC2B | oligonucleotide/oligosaccharide-binding fold containing 2B | 1,7441558 | 0,77218 | -3,14542 | 0 |  |
| 222460_s_at | NIP30 | NEFA-interacting nuclear protein NIP30 | 0,3743848 | 2,14393 | -3,14789 | 0 |  |
| 222846_at | RAB8B | RAB8B, member RAS oncogene family | 0,8492389 | 1,6697 | -3,14867 | 0 |  |
| 210202_s_at | BIN1 | bridging integrator 1 | 1,6552586 | 0,86517 | -3,15054 | 0 |  |
| 223452_s_at | DKFZP564J0863 | DKFZP564J0863 protein | 1,2880461 | 1,23779 | -3,1573 | 0 |  |
| 1553027_a_at | KLHL4 | kelch-like 4 (Drosophila) | 0,636723 | 1,88915 | -3,15734 | 0 |  |
| 220200_s_at | SETD8 /// LOC647597 | SET domain containing (lysine methyltransferase) 8 /// similar to Histone-lysine N-methyltransferase, H4 lysine-20 specific (Histone H4-K20 methyltransferase) (H4-K20-HMTase) (SET domain-containing protein 8) (PR/SET domain-containing protein 07) (PR/SET0 | 0,6415582 | 1,88705 | -3,16076 | 0 |  |
| 219238_at | GFRA1 /// PIGV | GDNF family receptor alpha 1 /// phosphatidylinositol glycan anchor biosynthesis, class V | 1,010229 | 1,51923 | -3,16183 | 0 |  |
| 208677_s_at | BSG | basigin (Ok blood group) | 1,8187974 | 0,71349 | -3,16536 | 0 |  |
| 1556827_at | LOC339929 | hypothetical protein LOC339929 | 0,7992235 | 1,73448 | -3,16713 | 0 |  |
| 229441_at | PRSS23 | Protease, serine, 23 | 0,5344749 | 2,00204 | -3,17064 | 0 |  |
| 1553962_s_at | RHOB | ras homolog gene family, member B | 1,715707 | 0,82228 | -3,17249 | 0 |  |
| 1555294_a_at | ERC1 | ELKS/RAB6-interacting/CAST family member 1 | 0,0731078 | 2,46666 | -3,17471 | 0 |  |
| 202290_at | PDAP1 | PDGFA associated protein 1 | 0,8727881 | 1,66765 | -3,17555 | 0 |  |
| 217960_s_at | TOMM22 | translocase of outer mitochondrial membrane 22 homolog (yeast) | 0,8166117 | 1,72407 | -3,17586 | 0 |  |
| 210987_x_at | TPM1 | tropomyosin 1 (alpha) | 0,3701808 | 2,18035 | -3,18816 | 0 |  |
| 217188_s_at | C14orf1 | chromosome 14 open reading frame 1 | 1,1051569 | 1,44751 | -3,19083 | 0 |  |
| 1555564_a_at | CFI | complement factor I | 0,9115599 | 1,64531 | -3,19609 | 0 |  |
| 214544_s_at | SNAP23 | synaptosomal-associated protein, 23kDa | 1,4041083 | 1,15397 | -3,19759 | 0 |  |
| 225238_at | MSI2 | musashi homolog 2 (Drosophila) | 1,1082372 | 1,45091 | -3,19893 | 0 |  |
| 205882_x_at | ADD3 | adducin 3 (gamma) | 0,7060346 | 1,85972 | -3,20719 | 0 |  |
| 201465_s_at | JUN | jun oncogene | 0,7437429 | 1,82696 | -3,21338 | 0 |  |
| 208078_s_at | SNF1LK | SNF1-like kinase /// SNF1-like kinase | 0,3543049 | 2,2269 | -3,22651 | 0 |  |
| 200660_at | S100A11 | S100 calcium binding protein A11 | 1,6746039 | 0,90964 | -3,23031 | 0 |  |
| 201043_s_at | ANP32A | acidic (leucine-rich) nuclear phosphoprotein 32 family, member A | 1,1552209 | 1,42968 | -3,23112 | 0 |  |
| 201224_s_at | SRRM1 | serine/arginine repetitive matrix 1 | 1,1642563 | 1,43171 | -3,24496 | 0 |  |
| 213470_s_at | HNRPH1 | heterogeneous nuclear ribonucleoprotein H1 (H) | 1,804196 | 0,79824 | -3,25305 | 0 |  |
| 201742_x_at | SFRS1 | splicing factor, arginine/serine-rich 1 (splicing factor 2, alternate splicing factor) | 1,6105718 | 0,99542 | -3,25749 | 0 |  |
| 211530_x_at | HLA-G | HLA-G histocompatibility antigen, class I, G | 1,1726865 | 1,4413 | -3,26749 | 0 |  |
| 215706_x_at | ZYX | zyxin | 1,1965046 | 1,41941 | -3,26989 | 0 |  |
| 214121_x_at | PDLIM7 | PDZ and LIM domain 7 (enigma) | 0,4849829 | 2,13365 | -3,27329 | 0 |  |
| 203557_s_at | PCBD1 | pterin-4 alpha-carbinolamine dehydratase/dimerization cofactor of hepatocyte nuclear factor 1 alpha (TCF1) | 1,3296581 | 1,29089 | -3,27569 | 0 |  |
| 209675_s_at | HNRPUL1 | heterogeneous nuclear ribonucleoprotein U-like 1 | 1,8217967 | 0,80037 | -3,2777 | 0 |  |
| 217831_s_at | NSFL1C | NSFL1 (p97) cofactor (p47) | 1,7913309 | 0,83091 | -3,2778 | 0 |  |
| 217909_s_at | MLX | MAX-like protein X | 0,7353635 | 1,89274 | -3,28513 | 0 |  |
| 228266_s_at | HDGFRP3 | hepatoma-derived growth factor, related protein 3 | 1,2822658 | 1,34922 | -3,28936 | 0 |  |
| 1552977_a_at | TNRC5 | trinucleotide repeat containing 5 | 0,532155 | 2,10021 | -3,29046 | 0 |  |
| 215071_s_at | HIST1H2AC | histone cluster 1, H2ac | 0,4729263 | 2,16927 | -3,30274 | 0 |  |
| 65133_i_at | ZNHIT4 | zinc finger, HIT type 4 | 0,6062064 | 2,04022 | -3,30803 | 0 |  |
| 214792_x_at | VAMP2 | vesicle-associated membrane protein 2 (synaptobrevin 2) | 1,5239248 | 1,1387 | -3,32828 | 0 |  |
| 204969_s_at | RDX | radixin | 1,6179224 | 1,04806 | -3,33248 | 0 |  |
| 201058_s_at | MYL9 | myosin, light chain 9, regulatory | 1,1546785 | 1,51514 | -3,33727 | 0 |  |
| 208579_x_at | HIST1H2BK | histone cluster 1, H2bk | 0,586703 | 2,08438 | -3,33885 | 0 |  |
| 209011_at | TRIO | triple functional domain (PTPRF interacting) | 1,0039383 | 1,67917 | -3,35388 | 0 |  |
| 226235_at | LOC339290 | hypothetical protein LOC339290 | -0,190232 | 2,87437 | -3,35517 | 0 |  |
| 208337_s_at | NR5A2 | nuclear receptor subfamily 5, group A, member 2 | 1,0649745 | 1,6314 | -3,37047 | 0 |  |
| 205761_s_at | DUS4L | dihydrouridine synthase 4-like (S. cerevisiae) | 0,4417526 | 2,2716 | -3,39169 | 0 |  |
| 201168_x_at | ARHGDIA /// LOC728908 | Rho GDP dissociation inhibitor (GDI) alpha /// similar to Rho GDP dissociation inhibitor (GDI) alpha | 1,4112836 | 1,30303 | -3,39289 | 0 |  |
| 1554679_a_at | LAPTM4B | lysosomal associated protein transmembrane 4 beta | 1,0000148 | 1,71483 | -3,39356 | 0 |  |
| 200806_s_at | HSPD1 | heat shock 60kDa protein 1 (chaperonin) | 1,5032437 | 1,2122 | -3,39431 | 0 |  |
| 238012_at | DPP7 | Dipeptidyl-peptidase 7 | -0,371373 | 3,09077 | -3,39925 | 0 |  |
| 1552274_at | PXK | PX domain containing serine/threonine kinase | 0,5625935 | 2,16514 | -3,40967 | 0 |  |
| 226429_at | KIAA1704 | KIAA1704 | 1,7020289 | 1,02611 | -3,41018 | 0 |  |
| 210966_x_at | LARP1 | La ribonucleoprotein domain family, member 1 | 1,0773439 | 1,65469 | -3,41504 | 0 |  |
| 1566303_s_at | PPP1R11 | protein phosphatase 1, regulatory (inhibitor) subunit 11 | 1,0328681 | 1,69976 | -3,41579 | 0 |  |
| 210338_s_at | HSPA8 | heat shock 70kDa protein 8 | 1,4244403 | 1,32964 | -3,4426 | 0 |  |
| 225801_at | MAFbx | CDNA FLJ39585 fis, clone SKMUS2006633 | 1,1247855 | 1,63877 | -3,45444 | 0 |  |
| 230748_at | SLC16A6 | solute carrier family 16, member 6 (monocarboxylic acid transporter 7) | -0,228013 | 3,00155 | -3,46692 | 0 |  |
| 211317_s_at | CFLAR | CASP8 and FADD-like apoptosis regulator | 1,7447491 | 1,03315 | -3,47238 | 0 |  |
| 208316_s_at | OCRL | oculocerebrorenal syndrome of Lowe | 0,9995193 | 1,78367 | -3,47899 | 0 |  |
| 201050_at | PLD3 | phospholipase D family, member 3 | 1,1126892 | 1,67506 | -3,48468 | 0 |  |
| 208973_at | PRNPIP | prion protein interacting protein | 1,5679988 | 1,22366 | -3,48958 | 0 |  |
| 211160_x_at | ACTN1 | actinin, alpha 1 | 0,5641222 | 2,23167 | -3,49474 | 0 |  |
| 200973_s_at | TSPAN3 | tetraspanin 3 | 1,0551262 | 1,7483 | -3,50428 | 0 |  |
| 210935_s_at | WDR1 | WD repeat domain 1 | 1,6332533 | 1,17321 | -3,50808 | 0 |  |
| 214390_s_at | BCAT1 | branched chain aminotransferase 1, cytosolic | 1,4026319 | 1,41967 | -3,52788 | 0 |  |
| 207172_s_at | CDH11 | cadherin 11, type 2, OB-cadherin (osteoblast) | 1,6632465 | 1,16819 | -3,5393 | 0 |  |
| 201118_at | PGD /// UGDH | phosphogluconate dehydrogenase /// phosphogluconate dehydrogenase /// UDP-glucose dehydrogenase /// UDP-glucose dehydrogenase | 1,2753241 | 1,56361 | -3,54867 | 0 |  |
| 209257_s_at | SMC3 | structural maintenance of chromosomes 3 | 1,1130652 | 1,73054 | -3,5545 | 0 |  |
| 201946_s_at | CCT2 | chaperonin containing TCP1, subunit 2 (beta) | 1,6688005 | 1,17623 | -3,55628 | 0 |  |
| 200769_s_at | MAT2A | methionine adenosyltransferase II, alpha | 0,440473 | 2,40503 | -3,55688 | 0 |  |
| 212298_at | NRP1 | neuropilin 1 | 0,8329425 | 2,02601 | -3,57369 | 0 |  |
| 201605_x_at | CNN2 | calponin 2 | 0,8402269 | 2,02218 | -3,57801 | 0 |  |
| 208932_at | PPP4C | protein phosphatase 4 (formerly X), catalytic subunit | 1,3093985 | 1,55305 | -3,57806 | 0 |  |
| 204560_at | FKBP5 | FK506 binding protein 5 | 1,5374672 | 1,32602 | -3,57936 | 0 |  |
| 224853_at | SLAIN2 | SLAIN motif family, member 2 | 0,7956401 | 2,08157 | -3,59651 | 0 |  |
| 1554980_a_at | ATF3 | activating transcription factor 3 | 1,4116741 | 1,47015 | -3,60227 | 0 |  |
| 210978_s_at | TAGLN2 | transgelin 2 | 1,6375051 | 1,26994 | -3,63431 | 0 |  |
| 202708_s_at | HIST2H2BE | histone cluster 2, H2be | 0,3530233 | 2,5563 | -3,63666 | 0 |  |
| 214845_s_at | CALU | calumenin | 1,618949 | 1,29261 | -3,63945 | 0 |  |
| 208808_s_at | HMGB2 | high-mobility group box 2 | 0,673786 | 2,23887 | -3,64083 | 0 |  |
| 232053_x_at | RHBDD2 | rhomboid domain containing 2 | 1,2626769 | 1,65656 | -3,64905 | 0 |  |
| 213826_s_at | Histone H3.3 |  | 0,687075 | 2,23378 | -3,65107 | 0 |  |
| 210153_s_at | ME2 /// PRKAR2B | malic enzyme 2, NAD(+)-dependent, mitochondrial /// protein kinase, cAMP-dependent, regulatory, type II, beta | 1,6149284 | 1,31047 | -3,65674 | 0 |  |
| 209372_x_at | TUBB2A /// TUBB2B | tubulin, beta 2A /// tubulin, beta 2B | 1,1416134 | 1,78397 | -3,65698 | 0 |  |
| 211136_s_at | CLPTM1 | cleft lip and palate associated transmembrane protein 1 | 0,9796087 | 1,94749 | -3,65887 | 0 |  |
| 201234_at | ILK | integrin-linked kinase | 0,6459226 | 2,28345 | -3,66172 | 0 |  |
| 208703_s_at | APLP2 | amyloid beta (A4) precursor-like protein 2 | 1,0041639 | 1,93217 | -3,67042 | 0 |  |
| 210529_s_at | KIAA0738 /// LOC653199 | KIAA0738 gene product /// hypothetical LOC653199 | 1,0654777 | 1,87248 | -3,67245 | 0 |  |
| 1555618_s_at | SAE1 | SUMO1 activating enzyme subunit 1 | 1,1534717 | 1,78855 | -3,67753 | 0 |  |
| 200709_at | FKBP1A | FK506 binding protein 1A, 12kDa | 1,9282157 | 1,01752 | -3,68218 | 0 |  |
| 210756_s_at | NOTCH2 | Notch homolog 2 (Drosophila) /// Notch homolog 2 (Drosophila) | 0,419727 | 2,52998 | -3,68713 | 0 |  |
| 205428_s_at | CALB2 | calbindin 2, 29kDa (calretinin) | 0,6382779 | 2,31803 | -3,69538 | 0 |  |
| 213746_s_at | FLNA | filamin A, alpha (actin binding protein 280) | 1,0331896 | 1,93188 | -3,70634 | 0 |  |
| 208698_s_at | NONO | non-POU domain containing, octamer-binding | 1,425424 | 1,54271 | -3,71017 | 0 |  |
| 231715_s_at | PYCR2 | pyrroline-5-carboxylate reductase family, member 2 | 1,7665991 | 1,21607 | -3,72834 | 0 |  |
| 200008_s_at | GDI2 | GDP dissociation inhibitor 2 /// GDP dissociation inhibitor 2 | 1,4651407 | 1,52035 | -3,73187 | 0 |  |
| 218000_s_at | PHLDA1 | pleckstrin homology-like domain, family A, member 1 | 1,7396634 | 1,26739 | -3,75881 | 0 |  |
| 218880_at | FOSL2 | FOS-like antigen 2 | 1,530344 | 1,48136 | -3,76463 | 0 |  |
| 213901_x_at | RBM9 | RNA binding motif protein 9 | 1,4489052 | 1,57181 | -3,7759 | 0 |  |
| 209435_s_at | ARHGEF2 | rho/rac guanine nucleotide exchange factor (GEF) 2 | 0,3581953 | 2,66293 | -3,7764 | 0 |  |
| 211622_s_at | ARF3 | ADP-ribosylation factor 3 /// ADP-ribosylation factor 3 | 1,2852138 | 1,73947 | -3,78086 | 0 |  |
| 221771_s_at | HSMPP8 | M-phase phosphoprotein, mpp8 | 1,7209348 | 1,30491 | -3,78231 | 0 |  |
| 212552_at | HPCAL1 | hippocalcin-like 1 | 1,0295629 | 2,00456 | -3,79266 | 0 |  |
| 201123_s_at | EIF5A | eukaryotic translation initiation factor 5A | 1,7289832 | 1,32618 | -3,81895 | 0 |  |
| 217140_s_at | VDAC1 | voltage-dependent anion channel 1 | 1,4266869 | 1,64014 | -3,83353 | 0 |  |
| 209205_s_at | LMO4 | LIM domain only 4 | 0,629417 | 2,44112 | -3,83817 | 0 |  |
| 201490_s_at | PPIF | peptidylprolyl isomerase F (cyclophilin F) | 1,2079496 | 1,86513 | -3,84135 | 0 |  |
| 204306_s_at | CD151 | CD151 molecule (Raph blood group) | 0,5597905 | 2,51358 | -3,84171 | 0 |  |
| 201309_x_at | C5orf13 | chromosome 5 open reading frame 13 | 0,9738025 | 2,10435 | -3,84769 | 0 |  |
| 209772_s_at | CD24 | CD24 molecule | 0,4812598 | 2,59902 | -3,85035 | 0 |  |
| 201039_s_at | RAD23A | RAD23 homolog A (S. cerevisiae) | 1,3026602 | 1,78202 | -3,85586 | 0 |  |
| 233013_x_at | LOC220906 | hypothetical protein LOC220906 | 1,0243042 | 2,0722 | -3,87062 | 0 |  |
| 201389_at | ITGA5 | integrin, alpha 5 (fibronectin receptor, alpha polypeptide) | 1,6626206 | 1,43698 | -3,8745 | 0 |  |
| 208721_s_at | ANAPC5 | anaphase promoting complex subunit 5 | 0,957178 | 2,14878 | -3,88245 | 0 |  |
| 200900_s_at | M6PR | mannose-6-phosphate receptor (cation dependent) | 1,6057369 | 1,50734 | -3,89135 | 0 |  |
| 206929_s_at | NFIC | nuclear factor I/C (CCAAT-binding transcription factor) | 1,0228737 | 2,09237 | -3,89406 | 0 |  |
| 206075_s_at | CSNK2A1 | casein kinase 2, alpha 1 polypeptide | 1,8896413 | 1,22673 | -3,89546 | 0 |  |
| 1554451_s_at | CIP29 /// DNAJC14 | cytokine induced protein 29 kDa /// DnaJ (Hsp40) homolog, subfamily C, member 14 | 1,2735483 | 1,84743 | -3,90122 | 0 |  |
| 202431_s_at | MYC | v-myc myelocytomatosis viral oncogene homolog (avian) | 2,0528914 | 1,07999 | -3,9161 | 0 |  |
| 1554482_a_at | SAR1B | SAR1 gene homolog B (S. cerevisiae) | 1,7783228 | 1,36183 | -3,92519 | 0 |  |
| 209156_s_at | COL6A2 | collagen, type VI, alpha 2 | 1,3752788 | 1,76991 | -3,93149 | 0 |  |
| 214484_s_at | OPRS1 | opioid receptor, sigma 1 | 1,3089821 | 1,83656 | -3,93192 | 0 |  |
| 200787_s_at | PEA15 | phosphoprotein enriched in astrocytes 15 | 0,8249232 | 2,32933 | -3,94281 | 0 |  |
| 200751_s_at | HNRPC | heterogeneous nuclear ribonucleoprotein C (C1/C2) | 1,6508227 | 1,51733 | -3,96019 | 0 |  |
| 225507_at | C6orf111 | chromosome 6 open reading frame 111 | 2,2486395 | 0,91965 | -3,96037 | 0 |  |
| 200649_at | NUCB1 | nucleobindin 1 | 1,323707 | 1,86049 | -3,98025 | 0 |  |
| 201841_s_at | HSPB1 /// MEIS3 | heat shock 27kDa protein 1 /// Meis1, myeloid ecotropic viral integration site 1 homolog 3 (mouse) | 1,896549 | 1,3002 | -3,99594 | 0 |  |
| 223743_s_at | MRPL4 | mitochondrial ribosomal protein L4 | 2,1231932 | 1,07434 | -3,99692 | 0 |  |
| 223847_s_at | ERGIC1 | endoplasmic reticulum-golgi intermediate compartment (ERGIC) 1 | 1,4490134 | 1,75345 | -4,00308 | 0 |  |
| 203254_s_at | TLN1 | talin 1 | 0,7441089 | 2,46286 | -4,00871 | 0 |  |
| 216591_s_at | SDHC /// LOC642502 | succinate dehydrogenase complex, subunit C, integral membrane protein, 15kDa /// similar to succinate dehydrogenase complex, subunit C isoform 3 precursor | 1,3738239 | 1,83747 | -4,01411 | 0 |  |
| 210023_s_at | PCGF1 | polycomb group ring finger 1 | 1,7035177 | 1,51919 | -4,02838 | 0 |  |
| 212152_x_at | ARID1A | AT rich interactive domain 1A (SWI-like) | 0,6978421 | 2,5409 | -4,04843 | 0 |  |
| 201633_s_at | CYB5B | cytochrome b5 type B (outer mitochondrial membrane) | 0,6804874 | 2,56289 | -4,05423 | 0 |  |
| 242857_at | FARP2 | FERM, RhoGEF and pleckstrin domain protein 2 | 0,7998828 | 2,4464 | -4,05785 | 0 |  |
| 212009_s_at | STIP1 | stress-induced-phosphoprotein 1 (Hsp70/Hsp90-organizing protein) | 1,3895038 | 1,86397 | -4,06684 | 0 |  |
| 204149_s_at | GSTM4 | glutathione S-transferase M4 | 1,6676074 | 1,59246 | -4,07508 | 0 |  |
| 227219_x_at | MAP1LC3A | microtubule-associated protein 1 light chain 3 alpha | 1,2321313 | 2,02907 | -4,0765 | 0 |  |
| 214995_s_at | APOBEC3G /// APOBEC3F | apolipoprotein B mRNA editing enzyme, catalytic polypeptide-like 3G /// apolipoprotein B mRNA editing enzyme, catalytic polypeptide-like 3F | 1,8481804 | 1,42418 | -4,09046 | 0 |  |
| 1558028_x_at | LOC647979 | hypothetical protein LOC647979 | 1,969966 | 1,30964 | -4,09951 | 0 |  |
| 213872_at | C6orf62 | Chromosome 6 open reading frame 62 | 1,0016343 | 2,29331 | -4,11868 | 0 |  |
| 200964_at | UBE1 | ubiquitin-activating enzyme E1 (A1S9T and BN75 temperature sensitivity complementing) | 1,4775309 | 1,83186 | -4,13674 | 0 |  |
| 208611_s_at | SPTAN1 | spectrin, alpha, non-erythrocytic 1 (alpha-fodrin) | 1,459295 | 1,85497 | -4,14283 | 0 |  |
| 203085_s_at | TGFB1 | transforming growth factor, beta 1 (Camurati-Engelmann disease) | 1,7198743 | 1,60937 | -4,16156 | 0 |  |
| 204088_at | P2RX4 | purinergic receptor P2X, ligand-gated ion channel, 4 | 1,2619748 | 2,07296 | -4,16866 | 0 |  |
| 216976_s_at | RYK | RYK receptor-like tyrosine kinase | 0,9007677 | 2,43575 | -4,17064 | 0 |  |
| 202899_s_at | SFRS3 | splicing factor, arginine/serine-rich 3 | 0,6668375 | 2,68142 | -4,18532 | 0 |  |
| 204805_s_at | H1FX | H1 histone family, member X | 0,6239879 | 2,72432 | -4,18538 | 0 |  |
| 235219_at | LOC116349 | hypothetical protein BC014011 | 0,179381 | 3,18692 | -4,20787 | 0 |  |
| 208817_at | COMT | catechol-O-methyltransferase | 1,4729723 | 1,91342 | -4,23299 | 0 |  |
| 203890_s_at | DAPK3 | death-associated protein kinase 3 | 1,4773047 | 1,90953 | -4,23354 | 0 |  |
| 211681_s_at | PDLIM5 | PDZ and LIM domain 5 /// PDZ and LIM domain 5 | 1,1787043 | 2,21674 | -4,24431 | 0 |  |
| 201251_at | PKM2 | pyruvate kinase, muscle | 2,379848 | 1,04358 | -4,27929 | 0 |  |
| 224247_s_at | MRPS10 | mitochondrial ribosomal protein S10 | 1,1909692 | 2,26537 | -4,32042 | 0 |  |
| 201971_s_at | ATP6V1A | ATPase, H+ transporting, lysosomal 70kDa, V1 subunit A | 1,2414453 | 2,21606 | -4,32189 | 0 |  |
| 235277_at | AMOTL1 | angiomotin like 1 | 1,1903779 | 2,2814 | -4,33972 | 0 |  |
| 212003_at | C1orf144 | chromosome 1 open reading frame 144 | 2,0091842 | 1,50515 | -4,39291 | 0 |  |
| 1554574_a_at | CYB5R3 | cytochrome b5 reductase 3 | 2,0577601 | 1,45738 | -4,39392 | 0 |  |
| 208824_x_at | PCTK1 | PCTAIRE protein kinase 1 | 1,4567913 | 2,05905 | -4,39481 | 0 |  |
| 210154_at | ME2 | malic enzyme 2, NAD(+)-dependent, mitochondrial | 1,8502505 | 1,67758 | -4,40978 | 0 |  |
| 205321_at | EIF2S3 | eukaryotic translation initiation factor 2, subunit 3 gamma, 52kDa | 2,3673516 | 1,16941 | -4,42095 | 0 |  |
| 208751_at | NAPA | N-ethylmaleimide-sensitive factor attachment protein, alpha | 1,3597171 | 2,19493 | -4,44331 | 0 |  |
| 200695_at | PPP2R1A | protein phosphatase 2 (formerly 2A), regulatory subunit A (PR 65), alpha isoform | 1,275578 | 2,28744 | -4,45377 | 0 |  |
| 228851_s_at | ENSA | endosulfine alpha | 1,84505 | 1,72811 | -4,46645 | 0 |  |
| 203258_at | DRAP1 | DR1-associated protein 1 (negative cofactor 2 alpha) | 1,8043438 | 1,78142 | -4,4822 | 0 |  |
| 208876_s_at | PAK2 | p21 (CDKN1A)-activated kinase 2 | 2,3606321 | 1,23438 | -4,49376 | 0 |  |
| 210337_s_at | ACLY | ATP citrate lyase | 0,5103755 | 3,08654 | -4,49614 | 0 |  |
| 231974_at | MLL2 | myeloid/lymphoid or mixed-lineage leukemia 2 | 2,4532712 | 1,14983 | -4,50387 | 0 |  |
| 204427_s_at | TMED2 | transmembrane emp24 domain trafficking protein 2 | 2,1221415 | 1,48133 | -4,50434 | 0 |  |
| 202058_s_at | KPNA1 | karyopherin alpha 1 (importin alpha 5) | 1,3878669 | 2,22306 | -4,51366 | 0 |  |
| 222422_s_at | NDFIP1 | Nedd4 family interacting protein 1 | 1,5236511 | 2,08945 | -4,51637 | 0 |  |
| 217211_at | LOC390861 | similar to cytoplasmic beta-actin | 1,3389802 | 2,30926 | -4,5603 | 0 |  |
| 203311_s_at | ARF6 | ADP-ribosylation factor 6 | 0,8962544 | 2,75366 | -4,56239 | 0 |  |
| 201469_s_at | SHC1 | SHC (Src homology 2 domain containing) transforming protein 1 | 1,6249101 | 2,03449 | -4,57426 | 0 |  |
| 210317_s_at | YWHAE | tyrosine 3-monooxygenase/tryptophan 5-monooxygenase activation protein, epsilon polypeptide | 1,8089529 | 1,89203 | -4,62623 | 0 |  |
| 201028_s_at | CD99 | CD99 molecule | 1,5541292 | 2,1692 | -4,65417 | 0 |  |
| 236533_at | DDEF1 | development and differentiation enhancing factor 1 | 1,7753638 | 1,97079 | -4,68269 | 0 |  |
| 201052_s_at | PSMF1 | proteasome (prosome, macropain) inhibitor subunit 1 (PI31) | 1,7247442 | 2,0386 | -4,70418 | 0 |  |
| 214359_s_at | HSP90AB1 | heat shock protein 90kDa alpha (cytosolic), class B member 1 | 2,1445685 | 1,63497 | -4,72443 | 0 |  |
| 211672_s_at | ARPC4 | actin related protein 2/3 complex, subunit 4, 20kDa /// actin related protein 2/3 complex, subunit 4, 20kDa | 1,2852557 | 2,54308 | -4,78542 | 0 |  |
| 203729_at | EMP3 | epithelial membrane protein 3 | 1,4324544 | 2,39713 | -4,78698 | 0 |  |
| 1558015_s_at | ACTR2 | ARP2 actin-related protein 2 homolog (yeast) | 1,6222222 | 2,20869 | -4,78864 | 0 |  |
| 200707_at | PRKCSH | protein kinase C substrate 80K-H | 2,3109627 | 1,52131 | -4,79034 | 0 |  |
| 201551_s_at | LAMP1 | lysosomal-associated membrane protein 1 | 1,1909139 | 2,64281 | -4,79215 | 0 |  |
| 225294_s_at | TRAPPC1 | trafficking protein particle complex 1 | 1,8615842 | 2,08733 | -4,93614 | 0 |  |
| 1555814_a_at | RHOA | ras homolog gene family, member A | 1,8751607 | 2,08523 | -4,95049 | 0 |  |
| 233878_s_at | XRN2 | 5'-3' exoribonuclease 2 | 2,3602843 | 1,64279 | -5,00384 | 0 |  |
| 1554678_s_at | HNRPDL | heterogeneous nuclear ribonucleoprotein D-like | 1,2497564 | 2,76016 | -5,01239 | 0 |  |
| 201082_s_at | DCTN1 | dynactin 1 (p150, glued homolog, Drosophila) | 1,2403265 | 2,80913 | -5,06182 | 0 |  |
| 200613_at | AP2M1 | adaptor-related protein complex 2, mu 1 subunit | 1,653284 | 2,40414 | -5,07177 | 0 |  |
| 215780_s_at | SET /// LOC389168 /// LOC642869 | SET translocation (myeloid leukemia-associated) /// similar to Protein SET (Phosphatase 2A inhibitor I2PP2A) (I-2PP2A) (Template-activating factor I) (TAF-I) (HLA-DR-associated protein II) (PHAPII) (Inhibitor of granzyme A-activated DNase) (IGAAD) /// sim | 1,5407496 | 2,51672 | -5,07184 | 0 |  |
| 212595_s_at | DAZAP2 | DAZ associated protein 2 | 1,7782543 | 2,30177 | -5,10003 | 0 |  |
| 201378_s_at | UBAP2L | ubiquitin associated protein 2-like | 1,4757113 | 2,68224 | -5,19744 | 0 |  |
| 200948_at | MLF2 | myeloid leukemia factor 2 | 1,9770579 | 2,18939 | -5,20805 | 0 |  |
| 218260_at | C19orf58 | chromosome 19 open reading frame 58 | 1,6079932 | 2,59576 | -5,25469 | 0 |  |
| 202024_at | ASNA1 | arsA arsenite transporter, ATP-binding, homolog 1 (bacterial) | 1,7780202 | 2,48685 | -5,33109 | 0 |  |
| 208886_at | H1F0 | H1 histone family, member 0 | 1,3704891 | 2,90566 | -5,34519 | 0 |  |
| 213606_s_at | ARHGDIA | Rho GDP dissociation inhibitor (GDI) alpha | 3,758286 | 0,5271 | -5,35674 | 0 |  |
| 1564494_s_at | P4HB | procollagen-proline, 2-oxoglutarate 4-dioxygenase (proline 4-hydroxylase), beta polypeptide | 3,163208 | 1,12965 | -5,36607 | 0 |  |
| 201950_x_at | CAPZB | capping protein (actin filament) muscle Z-line, beta | 1,6089841 | 2,68785 | -5,37104 | 0 |  |
| 221269_s_at | SH3BGRL3 | SH3 domain binding glutamic acid-rich protein like 3 /// SH3 domain binding glutamic acid-rich protein like 3 | 2,8310639 | 1,47994 | -5,38876 | 0 |  |
| 211997_x_at | H3F3B | H3 histone, family 3B (H3.3B) | 1,2576878 | 3,07326 | -5,41369 | 0 |  |
| 208750_s_at | ARF1 | ADP-ribosylation factor 1 | 1,8847215 | 2,46045 | -5,43146 | 0 |  |
| 207993_s_at | CHP | calcium binding protein P22 | 2,4189248 | 1,94629 | -5,45652 | 0 |  |
| 1565717_s_at | FUS | fusion (involved in t(12;16) in malignant liposarcoma) | 2,5855105 | 1,8454 | -5,53864 | 0 |  |
| 221423_s_at | YIPF5 | Yip1 domain family, member 5 /// Yip1 domain family, member 5 | 2,4520435 | 2,10953 | -5,70197 | 0 |  |
| 200641_s_at | YWHAZ | tyrosine 3-monooxygenase/tryptophan 5-monooxygenase activation protein, zeta polypeptide | 1,7817788 | 2,85576 | -5,79692 | 0 |  |
| 201264_at | COPE | coatomer protein complex, subunit epsilon | 2,7297676 | 1,98673 | -5,89562 | 0 |  |
| 215399_s_at | OS9 | amplified in osteosarcoma | 2,3943374 | 2,35185 | -5,93273 | 0 |  |
| 200041_s_at | BAT1 | HLA-B associated transcript 1 /// HLA-B associated transcript 1 | 2,4699664 | 2,39313 | -6,07887 | 0 |  |
| 200634_at | PFN1 | profilin 1 | 2,572703 | 2,34801 | -6,15089 | 0 |  |
| 201040_at | GNAI2 | guanine nucleotide binding protein (G protein), alpha inhibiting activity polypeptide 2 | 2,5112801 | 2,4737 | -6,23123 | 0 |  |
| 225103_at | MRPL38 | mitochondrial ribosomal protein L38 | 1,9412445 | 3,06633 | -6,25946 | 0 |  |
| 217294_s_at | ENO1 | enolase 1, (alpha) | 3,3195812 | 1,69764 | -6,27153 | 0 |  |
| 37028_at | PPP1R15A | protein phosphatase 1, regulatory (inhibitor) subunit 15A | 1,191992 | 3,97251 | -6,45563 | 0 |  |
| 220477_s_at | C20orf30 | chromosome 20 open reading frame 30 | 1,7970275 | 3,58028 | -6,72164 | 0 |  |
| 1555730_a_at | CFL1 | cofilin 1 (non-muscle) | 2,7198785 | 2,72037 | -6,80031 | 0 |  |
| 1567107_s_at | TPM4 | tropomyosin 4 | 2,4794622 | 3,11115 | -6,98827 | 0 |  |
| 210125_s_at | BANF1 | barrier to autointegration factor 1 | 2,9700692 | 2,64109 | -7,01395 | 0 |  |
| 1555226_s_at | C1orf43 | chromosome 1 open reading frame 43 | 2,2341916 | 3,57612 | -7,26289 | 0 |  |
| 200001_at | CAPNS1 | calpain, small subunit 1 /// calpain, small subunit 1 | 2,3002954 | 3,8818 | -7,72761 | 0 |  |

Table SIII

| **Gene ID** | **Gene Name** | **Gene Title** | **CC younger (contrast-1)** | **CC median (contrast-2)** | **CC older (contrast-3)** | **q-value(%)** | **Category** |
| --- | --- | --- | --- | --- | --- | --- | --- |
| 222630_at | RFXDC2 | regulatory factor X domain containing 2 | -2,7411597 | -3,192458 | 7,41702 | 0 | CColder |
| 213221_s_at | SNF1LK2 | SNF1-like kinase 2 | -2,1745837 | -3,421025 | 6,99451 | 0 | CColder |
| 203255_at | FBXO11 | F-box protein 11 | -2,3357536 | -2,347275 | 5,85379 | 0 | CColder |
| 212781_at | RBBP6 | retinoblastoma binding protein 6 | -1,6964698 | -2,551227 | 5,30962 | 0 | CColder |
| 231870_s_at | NMD3 | NMD3 homolog (S. cerevisiae) | -2,1117308 | -2,049914 | 5,20206 | 0 | CColder |
| 209748_at | SPAST | spastin | -1,8953519 | -2,236368 | 5,16465 | 0 | CColder |
| 227980_at | HCG12 | HLA complex group 12 | -2,269962 | -1,810569 | 5,10066 | 0 | CColder |
| 203016_s_at | SSX2IP | synovial sarcoma, X breakpoint 2 interacting protein | -1,8543057 | -2,187746 | 5,05256 | 0 | CColder |
| 238949_at | LOC401805 | hypothetical gene supported by NM_144726 | -1,362221 | -2,650313 | 5,01567 | 0 | CColder |
| 212496_s_at | JMJD2B | jumonji domain containing 2B | -1,4024382 | -2,591933 | 4,99296 | 0 | CColder |
| 204671_s_at | ANKRD6 | ankyrin repeat domain 6 | -1,980016 | -1,836502 | 4,77065 | 0 | CColder |
| 226280_at | NIP2 | CDNA FLJ43545 fis, clone PROST2011631 | -2,0674841 | -1,747905 | 4,76924 | 0 | CColder |
| 224718_at | YY1 | YY1 transcription factor | -1,5373267 | -2,210571 | 4,68487 | 0 | CColder |
| 230903_s_at | C8orf42 | Chromosome 8 open reading frame 42 | -1,4785925 | -2,226232 | 4,63103 | 0 | CColder |
| 203966_s_at | PPM1A | protein phosphatase 1A (formerly 2C), magnesium-dependent, alpha isoform /// protein phosphatase 1A (formerly 2C), magnesium-dependent, alpha isoform | -1,5390851 | -2,076464 | 4,51944 | 0 | CColder |
| 202778_s_at | ZMYM2 | zinc finger, MYM-type 2 | -1,6621448 | -1,947035 | 4,51147 | 0 | CColder |
| 225544_at | TBX3 | T-box 3 (ulnar mammary syndrome) | -2,0489389 | -1,546993 | 4,49492 | 0 | CColder |
| 204847_at | ZBTB11 | zinc finger and BTB domain containing 11 | -1,3726178 | -2,154559 | 4,40897 | 0 | CColder |
| 1556060_a_at | KIAA1702 | KIAA1702 protein | -1,5933116 | -1,924682 | 4,39749 | 0 | CColder |
| 240557_at | TSC22D2 | TSC22 domain family, member 2 | -1,3563479 | -2,062693 | 4,2738 | 0 | CColder |
| 214336_s_at | COPA | coatomer protein complex, subunit alpha | 2,10688043 | -0,018292 | -2,6107 | 0 | CCyounger |
| 205214_at | STK17B | serine/threonine kinase 17b (apoptosis-inducing) | 1,71671597 | -4,58E-02 | -2,0886 | 0 | CCyounger |
| 227782_at | ZBTB7C | zinc finger and BTB domain containing 7C | 1,68584019 | -0,128977 | -1,9461 | 0 | CCyounger |
| 229411_at | PNCK | pregnancy upregulated non-ubiquitously expressed CaM kinase | 2,29812188 | -0,154459 | -2,6796 | 0 | CCyounger |
| 202145_at | LY6E | lymphocyte antigen 6 complex, locus E | 2,25855105 | -0,27816 | -2,4755 | 0 | CCyounger |
| 221484_at | B4GALT5 | UDP-Gal:betaGlcNAc beta 1,4- galactosyltransferase, polypeptide 5 | 2,66729056 | -0,840637 | -2,2833 | 0 | CCyounger |
| 217294_s_at | ENO1 | enolase 1, (alpha) | 3,3195812 | 1,6976395 | -6,2715 | 0 | CCyounger |
| 201264_at | COPE | coatomer protein complex, subunit epsilon | 2,72976763 | 1,9867263 | -5,8956 | 0 | CCyounger |
| 1565717_s_at | FUS | fusion (involved in t(12;16) in malignant liposarcoma) | 2,58551053 | 1,8454047 | -5,5386 | 0 | CCyounger |
| 221269_s_at | SH3BGRL3 | SH3 domain binding glutamic acid-rich protein like 3 /// SH3 domain binding glutamic acid-rich protein like 3 | 2,83106388 | 1,4799433 | -5,3888 | 0 | CCyounger |
| 1564494_s_at | P4HB | procollagen-proline, 2-oxoglutarate 4-dioxygenase (proline 4-hydroxylase), beta polypeptide | 3,16320804 | 1,1296507 | -5,3661 | 0 | CCyounger |
| 213606_s_at | ARHGDIA | Rho GDP dissociation inhibitor (GDI) alpha | 3,75828595 | 0,5271049 | -5,3567 | 0 | CCyounger |
| 233878_s_at | XRN2 | 5'-3' exoribonuclease 2 | 2,36028433 | 1,6427888 | -5,0038 | 0 | CCyounger |
| 214359_s_at | HSP90AB1 | heat shock protein 90kDa alpha (cytosolic), class B member 1 | 2,14456853 | 1,6349745 | -4,7244 | 0 | CCyounger |
| 204427_s_at | TMED2 | transmembrane emp24 domain trafficking protein 2 | 2,12214153 | 1,4813338 | -4,5043 | 0 | CCyounger |
| 205321_at | EIF2S3 | eukaryotic translation initiation factor 2, subunit 3 gamma, 52kDa | 2,36735158 | 1,1694109 | -4,421 | 0 | CCyounger |
| 220368_s_at | SMEK1 | SMEK homolog 1, suppressor of mek1 (Dictyostelium) | 2,09894857 | 0,310221 | -3,0115 | 0 | CCyounger |
| 201251_at | PKM2 | pyruvate kinase, muscle | 2,37984804 | 1,0435823 | -4,2793 | 0 | CCyounger |
| 223743_s_at | MRPL4 | mitochondrial ribosomal protein L4 | 2,12319316 | 1,0743447 | -3,9969 | 0 | CCyounger |
| 225507_at | C6orf111 | chromosome 6 open reading frame 111 | 2,24863949 | 0,9196529 | -3,9604 | 0 | CCyounger |
| 238012_at | DPP7 | Dipeptidyl-peptidase 7 | -0,3713733 | 3,0907705 | -3,3992 | 0 | CCmedian |
| 230748_at | SLC16A6 | solute carrier family 16, member 6 (monocarboxylic acid transporter 7) | -0,2280132 | 3,0015456 | -3,4669 | 0 | CCmedian |
| 210105_s_at | FYN | FYN oncogene related to SRC, FGR, YES | -0,5633744 | 3,0005004 | -3,0464 | 0 | CCmedian |
| 1554119_at | C16orf57 | chromosome 16 open reading frame 57 | -0,95092 | 2,8932727 | -2,4279 | 0 | CCmedian |
| 226235_at | LOC339290 | hypothetical protein LOC339290 | -0,1902324 | 2,8743665 | -3,3552 | 0 | CCmedian |
| 228834_at | TOB1 | transducer of ERBB2, 1 | -0,2800935 | 2,7333481 | -3,0666 | 0 | CCmedian |
| 225442_at | DDR2 | Discoidin domain receptor family, member 2 | -0,2349239 | 2,7214194 | -3,1081 | 0 | CCmedian |
| 231952_at | LOC731450 | hypothetical protein LOC731450 | -0,1294213 | 2,5698467 | -3,0505 | 0 | CCmedian |
| 226306_at | C6orf1 | chromosome 6 open reading frame 1 | -0,2723617 | 2,5697413 | -2,8717 | 0 | CCmedian |
| 228746_s_at | CDV3 | CDV3 homolog (mouse) | -0,1700435 | 2,2614408 | -2,6142 | 0 | CCmedian |
| 215253_s_at | DSCR1 | Down syndrome critical region gene 1 | -1,32E-02 | 2,2597755 | -2,8082 | 0 | CCmedian |
| 210753_s_at | EPHB1 | EPH receptor B1 | -0,3456899 | 2,2341471 | -2,3606 | 0 | CCmedian |
| 209398_at | HIST1H1C | histone cluster 1, H1c | -0,1467056 | 2,2223947 | -2,5946 | 0 | CCmedian |
| 207177_at | PTGFR | prostaglandin F receptor (FP) | -0,7052854 | 2,2060673 | -1,876 | 0 | CCmedian |
| 214649_s_at | MTMR2 | myotubularin related protein 2 | -9,26E-03 | 2,1938222 | -2,7307 | 0 | CCmedian |
| 213865_at | DCBLD2 | discoidin, CUB and LCCL domain containing 2 | -9,07E-03 | 2,1279996 | -2,6487 | 0 | CCmedian |
| 209409_at | GRB10 | growth factor receptor-bound protein 10 | -0,8774681 | 2,1119167 | -1,5431 | 0 | CCmedian |
| 203827_at | WIPI1 | WD repeat domain, phosphoinositide interacting 1 | -0,3334584 | 2,0770284 | -2,1795 | 0 | CCmedian |
| 238935_at | RPS27L | ribosomal protein S27-like | -0,3812622 | 2,0211386 | -2,0498 | 0 | CCmedian |
| 228948_at | EPHA4 | EPH receptor A4 | -0,0617032 | 2,0174102 | -2,4446 | 0 | CCmedian |

**Table SIV**

| **176 miRNAs CCyoung-med-old** |  | **71 miRNAs CCyoung-med** |  | **33 miRNAs CCold** |  | **1 miRNA CCyoung-old** |  | **1 miRNA CCmed-old** |  | **3 miRNA CC med** |  | **1 miRNA CCyoung** |
| --- | --- | --- | --- | --- | --- | --- | --- | --- | --- | --- | --- | --- |
| miR-99a |  | miR-92b |  | miR-885 |  | miR-20b |  | miR-668 |  | miR-573 |  | miR-524 |
| miR-98 |  | miR-92 |  | miR-874 |  |  |  |  |  | miR-450b |  |  |
| miR-96 |  | miR-886 |  | miR-762 |  |  |  |  |  | miR-329 |  |  |
| miR-93 |  | miR-760 |  | miR-758 |  |  |  |  |  |  |  |  |
| miR-92a |  | miR-744 |  | miR-675 |  |  |  |  |  |  |  |  |
| miR-9 |  | miR-671 |  | miR-654 |  |  |  |  |  |  |  |  |
| miR-876 |  | miR-650 |  | miR-630 |  |  |  |  |  |  |  |  |
| miR-708 |  | miR-646 |  | miR-622 |  |  |  |  |  |  |  |  |
| miR-7 |  | miR-644a |  | miR-616 |  |  |  |  |  |  |  |  |
| miR-663a |  | miR-637 |  | miR-615 |  |  |  |  |  |  |  |  |
| miR-603 |  | miR-634 |  | miR-590 |  |  |  |  |  |  |  |  |
| miR-582 |  | miR-631 |  | miR-558 |  |  |  |  |  |  |  |  |
| miR-574 |  | miR-625 |  | miR-532 |  |  |  |  |  |  |  |  |
| miR-542 |  | miR-609 |  | miR-520g |  |  |  |  |  |  |  |  |
| miR-520b |  | miR-608 |  | miR-519d |  |  |  |  |  |  |  |  |
| miR-519a |  | miR-605 |  | miR-518c |  |  |  |  |  |  |  |  |
| miR-518a |  | miR-579 |  | miR-517c |  |  |  |  |  |  |  |  |
| miR-512 |  | miR-567 |  | miR-517a |  |  |  |  |  |  |  |  |
| miR-506 |  | miR-552 |  | miR-515 |  |  |  |  |  |  |  |  |
| miR-503 |  | miR-544b |  | miR-493 |  |  |  |  |  |  |  |  |
| miR-499 |  | miR-541 |  | miR-466 |  |  |  |  |  |  |  |  |
| miR-497 |  | miR-525 |  | miR-452 |  |  |  |  |  |  |  |  |
| miR-494 |  | miR-520f |  | miR-448 |  |  |  |  |  |  |  |  |
| miR-492 |  | miR-520e |  | miR-431 |  |  |  |  |  |  |  |  |
| miR-491 |  | miR-520d |  | miR-377 |  |  |  |  |  |  |  |  |
| miR-486 |  | miR-520c |  | miR-301a |  |  |  |  |  |  |  |  |
| miR-485 |  | miR-520a |  | miR-219 |  |  |  |  |  |  |  |  |
| miR-483 |  | miR-519e |  | miR-208a |  |  |  |  |  |  |  |  |
| miR-455 |  | miR-513a |  | miR-202 |  |  |  |  |  |  |  |  |
| miR-451 |  | miR-511 |  | miR-18b |  |  |  |  |  |  |  |  |
| miR-449a |  | miR-504 |  | miR-184 |  |  |  |  |  |  |  |  |
| miR-429 |  | miR-495 |  | miR-130b |  |  |  |  |  |  |  |  |
| miR-424 |  | miR-490 |  | miR-let-7e |  |  |  |  |  |  |  |  |
| miR-409 |  | miR-488 |  |  |  |  |  |  |  |  |  |  |
| miR-383 |  | miR-449c |  |  |  |  |  |  |  |  |  |  |
| miR-382 |  | miR-433 |  |  |  |  |  |  |  |  |  |  |
| miR-381 |  | miR-432 |  |  |  |  |  |  |  |  |  |  |
| miR-378 |  | miR-425 |  |  |  |  |  |  |  |  |  |  |
| miR-376c |  | miR-421 |  |  |  |  |  |  |  |  |  |  |
| miR-376a |  | miR-380 |  |  |  |  |  |  |  |  |  |  |
| miR-375 |  | miR-367 |  |  |  |  |  |  |  |  |  |  |
| miR-374b |  | miR-362 |  |  |  |  |  |  |  |  |  |  |
| miR-374a |  | miR-361 |  |  |  |  |  |  |  |  |  |  |
| miR-373 |  | miR-346 |  |  |  |  |  |  |  |  |  |  |
| miR-372 |  | miR-345 |  |  |  |  |  |  |  |  |  |  |
| miR-370 |  | miR-340 |  |  |  |  |  |  |  |  |  |  |
| miR-369 |  | miR-339 |  |  |  |  |  |  |  |  |  |  |
| miR-365 |  | miR-337 |  |  |  |  |  |  |  |  |  |  |
| miR-363 |  | miR-331 |  |  |  |  |  |  |  |  |  |  |
| miR-34c |  | miR-326 |  |  |  |  |  |  |  |  |  |  |
| miR-34b |  | miR-323b |  |  |  |  |  |  |  |  |  |  |
| miR-34a |  | miR-320c |  |  |  |  |  |  |  |  |  |  |
| miR-342 |  | miR-302e |  |  |  |  |  |  |  |  |  |  |
| miR-33b |  | miR-299 |  |  |  |  |  |  |  |  |  |  |
| miR-33a |  | miR-298 |  |  |  |  |  |  |  |  |  |  |
| miR-338 |  | miR-28 |  |  |  |  |  |  |  |  |  |  |
| miR-335 |  | miR-216b |  |  |  |  |  |  |  |  |  |  |
| miR-328 |  | miR-215 |  |  |  |  |  |  |  |  |  |  |
| miR-324 |  | miR-196b |  |  |  |  |  |  |  |  |  |  |
| miR-320 |  | miR-190 |  |  |  |  |  |  |  |  |  |  |
| miR-32 |  | miR-187 |  |  |  |  |  |  |  |  |  |  |
| miR-31 |  | miR-181d |  |  |  |  |  |  |  |  |  |  |
| miR-30e |  | miR-181c |  |  |  |  |  |  |  |  |  |  |
| miR-30d |  | miR-152 |  |  |  |  |  |  |  |  |  |  |
| miR-30c |  | miR-151 |  |  |  |  |  |  |  |  |  |  |
| miR-30b |  | miR-149 |  |  |  |  |  |  |  |  |  |  |
| miR-30a |  | miR-147 |  |  |  |  |  |  |  |  |  |  |
| miR-302d |  | miR-146b |  |  |  |  |  |  |  |  |  |  |
| miR-302c |  | miR-128a |  |  |  |  |  |  |  |  |  |  |
| miR-302b |  | miR-127 |  |  |  |  |  |  |  |  |  |  |
| miR-302a |  | miR-let-7d |  |  |  |  |  |  |  |  |  |  |
| miR-301b |  |  |  |  |  |  |  |  |  |  |  |  |
| miR-29c |  |  |  |  |  |  |  |  |  |  |  |  |
| miR-29b |  |  |  |  |  |  |  |  |  |  |  |  |
| miR-29a |  |  |  |  |  |  |  |  |  |  |  |  |
| miR-297 |  |  |  |  |  |  |  |  |  |  |  |  |
| miR-296 |  |  |  |  |  |  |  |  |  |  |  |  |
| miR-27b |  |  |  |  |  |  |  |  |  |  |  |  |
| miR-27a |  |  |  |  |  |  |  |  |  |  |  |  |
| miR-26b |  |  |  |  |  |  |  |  |  |  |  |  |
| miR-26a |  |  |  |  |  |  |  |  |  |  |  |  |
| miR-25 |  |  |  |  |  |  |  |  |  |  |  |  |
| miR-24 |  |  |  |  |  |  |  |  |  |  |  |  |
| miR-23b |  |  |  |  |  |  |  |  |  |  |  |  |
| miR-23a |  |  |  |  |  |  |  |  |  |  |  |  |
| miR-224 |  |  |  |  |  |  |  |  |  |  |  |  |
| miR-223 |  |  |  |  |  |  |  |  |  |  |  |  |
| miR-222 |  |  |  |  |  |  |  |  |  |  |  |  |
| miR-221 |  |  |  |  |  |  |  |  |  |  |  |  |
| miR-22 |  |  |  |  |  |  |  |  |  |  |  |  |
| miR-218 |  |  |  |  |  |  |  |  |  |  |  |  |
| miR-216a |  |  |  |  |  |  |  |  |  |  |  |  |
| miR-214 |  |  |  |  |  |  |  |  |  |  |  |  |
| miR-212 |  |  |  |  |  |  |  |  |  |  |  |  |
| miR-211 |  |  |  |  |  |  |  |  |  |  |  |  |
| miR-210 |  |  |  |  |  |  |  |  |  |  |  |  |
| miR-21 |  |  |  |  |  |  |  |  |  |  |  |  |
| miR-20a |  |  |  |  |  |  |  |  |  |  |  |  |
| miR-206 |  |  |  |  |  |  |  |  |  |  |  |  |
| miR-205 |  |  |  |  |  |  |  |  |  |  |  |  |
| miR-204 |  |  |  |  |  |  |  |  |  |  |  |  |
| miR-203 |  |  |  |  |  |  |  |  |  |  |  |  |
| miR-200c |  |  |  |  |  |  |  |  |  |  |  |  |
| miR-200b |  |  |  |  |  |  |  |  |  |  |  |  |
| miR-200a |  |  |  |  |  |  |  |  |  |  |  |  |
| miR-19b |  |  |  |  |  |  |  |  |  |  |  |  |
| miR-19a |  |  |  |  |  |  |  |  |  |  |  |  |
| miR-199b |  |  |  |  |  |  |  |  |  |  |  |  |
| miR-199a |  |  |  |  |  |  |  |  |  |  |  |  |
| miR-196a |  |  |  |  |  |  |  |  |  |  |  |  |
| miR-195 |  |  |  |  |  |  |  |  |  |  |  |  |
| miR-194 |  |  |  |  |  |  |  |  |  |  |  |  |
| miR-193b |  |  |  |  |  |  |  |  |  |  |  |  |
| miR-193a |  |  |  |  |  |  |  |  |  |  |  |  |
| miR-193 |  |  |  |  |  |  |  |  |  |  |  |  |
| miR-192 |  |  |  |  |  |  |  |  |  |  |  |  |
| miR-191 |  |  |  |  |  |  |  |  |  |  |  |  |
| miR-18a |  |  |  |  |  |  |  |  |  |  |  |  |
| miR-186 |  |  |  |  |  |  |  |  |  |  |  |  |
| miR-185 |  |  |  |  |  |  |  |  |  |  |  |  |
| miR-183 |  |  |  |  |  |  |  |  |  |  |  |  |
| miR-182 |  |  |  |  |  |  |  |  |  |  |  |  |
| miR-181b |  |  |  |  |  |  |  |  |  |  |  |  |
| miR-181a |  |  |  |  |  |  |  |  |  |  |  |  |
| miR-17 |  |  |  |  |  |  |  |  |  |  |  |  |
| miR-16 |  |  |  |  |  |  |  |  |  |  |  |  |
| miR-15b |  |  |  |  |  |  |  |  |  |  |  |  |
| miR-15a |  |  |  |  |  |  |  |  |  |  |  |  |
| miR-155 |  |  |  |  |  |  |  |  |  |  |  |  |
| miR-154 |  |  |  |  |  |  |  |  |  |  |  |  |
| miR-153 |  |  |  |  |  |  |  |  |  |  |  |  |
| miR-150 |  |  |  |  |  |  |  |  |  |  |  |  |
| miR-148b |  |  |  |  |  |  |  |  |  |  |  |  |
| miR-148a |  |  |  |  |  |  |  |  |  |  |  |  |
| miR-146a |  |  |  |  |  |  |  |  |  |  |  |  |
| miR-145 |  |  |  |  |  |  |  |  |  |  |  |  |
| miR-144 |  |  |  |  |  |  |  |  |  |  |  |  |
| miR-143 |  |  |  |  |  |  |  |  |  |  |  |  |
| miR-142 |  |  |  |  |  |  |  |  |  |  |  |  |
| miR-141 |  |  |  |  |  |  |  |  |  |  |  |  |
| miR-140 |  |  |  |  |  |  |  |  |  |  |  |  |
| miR-139 |  |  |  |  |  |  |  |  |  |  |  |  |
| miR-138 |  |  |  |  |  |  |  |  |  |  |  |  |
| miR-137 |  |  |  |  |  |  |  |  |  |  |  |  |
| miR-136 |  |  |  |  |  |  |  |  |  |  |  |  |
| miR-135b |  |  |  |  |  |  |  |  |  |  |  |  |
| miR-135a |  |  |  |  |  |  |  |  |  |  |  |  |
| miR-134 |  |  |  |  |  |  |  |  |  |  |  |  |
| miR-133b |  |  |  |  |  |  |  |  |  |  |  |  |
| miR-133a |  |  |  |  |  |  |  |  |  |  |  |  |
| miR-132 |  |  |  |  |  |  |  |  |  |  |  |  |
| miR-130a |  |  |  |  |  |  |  |  |  |  |  |  |
| miR-129 |  |  |  |  |  |  |  |  |  |  |  |  |
| miR-128 |  |  |  |  |  |  |  |  |  |  |  |  |
| miR-126 |  |  |  |  |  |  |  |  |  |  |  |  |
| miR-125b |  |  |  |  |  |  |  |  |  |  |  |  |
| miR-125a |  |  |  |  |  |  |  |  |  |  |  |  |
| miR-124a |  |  |  |  |  |  |  |  |  |  |  |  |
| miR-124 |  |  |  |  |  |  |  |  |  |  |  |  |
| miR-122a |  |  |  |  |  |  |  |  |  |  |  |  |
| miR-122 |  |  |  |  |  |  |  |  |  |  |  |  |
| miR-10b |  |  |  |  |  |  |  |  |  |  |  |  |
| miR-10a |  |  |  |  |  |  |  |  |  |  |  |  |
| miR-107 |  |  |  |  |  |  |  |  |  |  |  |  |
| miR-106b |  |  |  |  |  |  |  |  |  |  |  |  |
| miR-106a |  |  |  |  |  |  |  |  |  |  |  |  |
| miR-103 |  |  |  |  |  |  |  |  |  |  |  |  |
| miR-101 |  |  |  |  |  |  |  |  |  |  |  |  |
| miR-100 |  |  |  |  |  |  |  |  |  |  |  |  |
| miR-1 |  |  |  |  |  |  |  |  |  |  |  |  |
| miR-let-7i |  |  |  |  |  |  |  |  |  |  |  |  |
| miR-let-7g |  |  |  |  |  |  |  |  |  |  |  |  |
| miR-let-7f |  |  |  |  |  |  |  |  |  |  |  |  |
| miR-let-7c |  |  |  |  |  |  |  |  |  |  |  |  |
| miR-let-7b |  |  |  |  |  |  |  |  |  |  |  |  |
| miR-let-7a |  |  |  |  |  |  |  |  |  |  |  |  |

**Table SV**

| **Validated gene target of miRNA** | **Name of gene** | **MicroRNAs** | **Effect** | **Link Info** | **References (PMID)** |
| --- | --- | --- | --- | --- | --- |
| **Inflammatory genes** | |  |  |  |  |
| IL1R1 | interleukin 1 receptor, type I | miR- 122a | Inhibition | it was shown a positive effect of miR-122 overexpression on gene IL1RI expression. | 19015728 |
| OSMR | oncostatin M receptor | miR- 122a | Inhibition | MicroRNA 122 physically interacts with OSMR and decreases its activity. | 19296470 |
| PRDX2 | peroxiredoxin 2 | miR- 122a | Inhibition | miR-122a targets 5'-UTR of PRDXII. | 20859956 |
|  |  | miR-671 | Inhibition | FN1 is a direct target of miR671. | 22637644 |
|  |  | miR-512 | Inhibition | miR-491, miR-671, and miR-512-3p bind to FN1 3'UTR. | 22393236;22637644 |
|  |  | miR-491 | Inhibition | miR-491, miR-671, and miR-512-3p bind to FN1 3'UTR. | 22393236;22637644 |
|  |  | miR-372 | Inhibition | Fibronectin is a putative target of miR-302b and miR-372. | 21490602 |
| FN1 | fibronectin 1 | miR-302b | Inhibition | Fibronectin is a putative target of miR-302b and miR-372. | 21490602 |
|  |  | miR-200c | Inhibition | Fibronectin is a direct target of miR-200c. | 19435871;21501518 |
|  |  | miR-17 | Inhibition | Fibronectin and the fibronectin type-III domain containing 3A (FNDC3A) are two targets that have their expression repressed by miR-17, both in vitro and in transgenic mice. | 19633662 |
|  |  | miR-146a | Inhibition | miR-146a targets the FN 3'-UTR. | 21885871;22363568 |
|  |  | miR-1 | Inhibition | FN1 is a direct target gene for miR-1. | 21924268;22210864;22454450 |
| ITGA2 | integrin, alpha 2 (CD49B, alpha 2 subunit of VLA-2 receptor) | miR-31 | Inhibition | miR-31 directly targets the 3'UTR of ITGA2, ITGA5, ITGAV and ITGB3 integrins and represses their expression. | 21875932 |
|  |  | miR-30a | Inhibition | MicroRNA 30a inhibits ITGA2 expression. | 18668040 |
| **Insulin signalling** | |  |  |  |  |
| INSR | insulin receptor | miR-192 | Inhibition | INSR (insulin receptor) is a predicted target of miR192. | 21829658;23032062 |
|  |  | miR-29b | Inhibition | p85alpha 3' UTR is normally targeted by endogenous miR-29 miRNAs as was determined by luciferase reporter assay and western blotting. | 19079265;20006673;21145728 |
|  |  | miR-17 | Inhibition | PI3K reg class IA gene (PIK3R1) is predicted microRNA 17 target. Decrease of PIK3R expression by this microRNA is confirmed. | 20148420 |
|  |  | miR-486 | Inhibition | MicroRNA 486 probably regulates expression of p85-alpha. | 20142475;20148420;20881268;21824387 |
|  |  | miR-376a | Inhibition | p85 alpha is a putative target of miR-376a. | 22350160;22684007 |
| PIK3R1 | phosphoinositide-3-kinase, regulatory subunit 1 (p85 alpha) | miR-29a | Inhibition | p85-alpha 3' UTR is normally targeted by endogenous miR-29 miRNAs in HeLa cells as was determined by luciferase reporter assay and western blotting. | 19079265;20006673;21693621;22646479 |
|  |  | miR-221 | Inhibition | miR-221 regulates the p85 alpha 3'UTR | 20505758;21501493;21756067;22350160;22931291 |
|  |  | miR-29c | Inhibition | p85alpha 3' UTR is normally targeted by endogenous miR-29 miRNAs in HeLa cells as was determined by luciferase reporter assay and western blotting. | 19079265;20006673;22646479 |
|  |  | miR-122a | Inhibition | it was shown a negative effect of miR-122 overexpression on gene pikr1 expression. | 19015728 |
|  |  | miR-320 | Inhibition | Using bioinformatic techniques, the p85 subunit of phosphatidylinositol 3-kinase (PI3-K) was found to be a potential target of microRNA 320. | 19473196 |
|  |  | miR-155 | Inhibition | p85-alpha is a common predicted target for miR-155 and miR-221/222. | 21364759;21501493;21756067;21989846;22511990;22609116 |
|  |  | miR-210 | Inhibition | IGFBP3 (IBP3) is validated miR-210 target. | 23028679 |
| IGFBP3 | insulin-like growth factor binding protein 3 | miR-125b | Inhibition | miR-125b directly represses Bak1, Igfbp3, Itch, Puma, Prkra, Tp53inp1, Tp53, Zac1, and also cell-cycle regulators like cyclin C, Cdc25c, Cdkn2c, Edn1, Ppp1ca, Sel1l mRNAs. | 21935352 |
|  |  | miR-122a | Inhibition | it was shown a negaitive effect of miR-122 overexpression on gene IBP3 expression. | 19015728 |
|  |  | miR-193b | Inhibition | IBP5 (IGFBP5) has been predicted to be the microRNA 193b target, using at least two algorithms from three ones (TargetScan 5.1, PicTar, MiRanda). | 20357817 |
| IGFBP5 | insulin-like growth factor binding protein 5 | miR-140 | Inhibition | IGFBP-5 is a direct target of miR-140. | 19948051;20466812;21325061 |
|  |  | miR-206 | Inhibition | IGF-binding protein 5 (Igfbp5) is a miR-206 target gene. | 22546853 |
| CRK | v-crk sarcoma virus CT10 oncogene homolog (avian) | miR-126 | Inhibition | Crk is a predicted putative target gene for miR-126. Over-expression of microRNA 126 in a lung cancer cell line resulted in a decrease in CRK protein without any alteration in the associated mRNA. | 18602365;18832181;19262572;20619534;21501493;21563499;22064652;22384141 |
|  |  | miR-132 | Inhibition | Crk is a potential gene target of miRNA 132. | 22499850 |
| CRKL | v-crk sarcoma virus CT10 oncogene homolog (avian)-like | miR-107 | Inhibition | microRNA 107 can target 3' UTR of the CrkL mRNA. | 19688090 |
|  |  | miR-1 | Inhibition | Both Calm1 and Calm2 contain highly conserved miR-1 seed match sequences in their 3' UTRs. | 19188439;21169019;21385380;22210864;23024758 |
| CALM1 | calmodulin 1 (phosphorylase kinase, delta) | miR-99a | Inhibition | Calm2 is a validated target of miR-99a and miR-99b. | 22299047 |
|  |  | miR-99b | Inhibition | Calm2 is a validated target of miR-99a and miR-99b. | 22299047 |
| SORT1 | sortilin 1 | miR-122 | Inhibition | Sortilin is a putative target of miR-122. | 20739924 |
|  |  | miR- 122a | Inhibition | Sortilin Is a Downstream Target of miR-122. | 19296470 |
| **Angiogenesis** | |  |  |  |  |
|  |  | miR-107 | Inhibition | TGF-beta receptor type III has been predicted as microRNA 107 target, using three algorithms TargetscanS, MiRanda and PicTar. | 18061676;18922750;19260130 |
| TGFBR3 | transforming growth factor, beta receptor III (betaglycan, 300kDa) | miR-134 | Inhibition | TGFBR3 is a target of microRNA 134. | 21228099 |
|  |  | miR-21 | Inhibition | Cotransfection with pre-miR-21, but not prescrambled sequencespecifically decreased luciferase levels of the reporter of JMY, TGFBR2, TGFBR3, HNRPK, TP73L (TAp63), APAF1, BMPR2, TOPORS, DAXX, TP53BP2, and PPIF. | 18829576;22960625 |
| ANGPTL4 | angiopoietin-like 4 | miR-29b | Inhibition | miR-29b inhibits metastasis by targeting a network of pro-metastatic regulators involved in angiogenesis | 23354167 |
| VEGFC | vascular endothelial growth factor C | miR-27b | Inhibition | VEGF-C is a predicted target of miR-27b. | 22049531 |
|  |  | miR-200c | Inhibition | Using PicTar algorithm and NCI-60 set of expression data, leptin receptor was predicted to be a microRNA 200c target with 5 binding sites. | 17697356;18483486;19435871;21501518 |
| LEPR | leptin receptor | miR-21 | Inhibition | miR-21 and 130a target LepR and EGR3 in primary human keratinocytes. | 22773832 |
|  |  | miR-130a | Inhibition | miR-21 and 130a target LepR and EGR3 in primary human keratinocytes. | 22773832 |
|  |  | miR-30d | Inhibition | APG5 is a predicted target of miR-30d. | 22058146 |
| ATG5 | ATG5 autophagy related 5 homolog (S. cerevisiae) | miR-374a | Inhibition | APG5 is a target of miR-374a. | 22356768 |
|  |  | miR-181a | Inhibition | APG5 (ATG5) was predicted to be miR-181a target. | 22356768;22442671 |
|  |  | miR-135b | Inhibition | COL15A1 ia s putative target of hsa-miR-135b. | 19795981 |
| COL15A1 | collagen, type XV, alpha 1 | miR-29c | Inhibition | microRNA 29c physically interacts with COL15A1 3' mRNAand decreases its activity. | 18390668;22745231 |
|  |  | miR- 122a | Inhibition | it was shown a positive effect of miR-122 overexpression on gene COL15A1 expression. | 19015728 |
|  |  | miR-941 | Inhibition | miR-941 binds to and inhibits FGF2 . | 23093182 |
|  |  | miR-15a | Inhibition | miR-15a directly targets FGF2 and VEGF to facilitate its anti-angiogenic effects. | 22692216 |
| FGF2 | fibroblast growth factor 2 (basic) | miR-15b | Inhibition | By using TargetScan software it was found that 3'UTR of FGF2 gene is a target for microrna 15b. | 19737397;20564181;21416501 |
|  |  | miR-424 | Inhibition | FGF2 is described as experimentally validated miR-424 target. | 22965126 |
|  |  | miR-16 | Inhibition | FGF2 is a putative target of miR-16. | 21602271;22589088 |
|  |  | miR-302b | Inhibition | Human NR2F2 3'UTR has putative binding sites for microRNAs 302/106, 27a/b, 101, 194, 128, 144 and 30abcde. | 21151097 |
| NR2F2 | nuclear receptor subfamily 2, group F, member 2 | miR-302c | Inhibition | Human NR2F2 3'UTR has putative binding sites for microRNAs 302/106, 27a/b, 101, 194, 128, 144 and 30abcde. | 21151097 |
|  |  | miR-302d | Inhibition | Human NR2F2 3'UTR has putative binding sites for microRNAs 302/106, 27a/b, 101, 194, 128, 144 and 30abcde. | 21151097 |
|  |  | miR-302a | Inhibition | NR2F2 (COUP-TFII) has been predicted to be the microRNA 302a target. | 18710938;21151097 |
|  |  | miR-210 | Inhibition | Tcf7l2 is a direct target of miR-210. | 20492721 |
| TCF7L2 | transcription factor 7-like 2 (T-cell specific, HMG-box) | miR-155 | Inhibition | TCF4 is a direct target of miR-155. | 21460854;22291592;22705797 |
|  |  | miR-204 | Inhibition | AP1S2, Bcl2l2, BIRC2, EDEM1, EZR, FZD1, M6PR, RAB22A, RAB40B, SERP1, TCF12 and TCF4) are direct targets of miR-204. | 21282569 |
|  |  | miR-93 | Inhibition | TCF4 is a mir93 direct target in SUM159 cells. | 22685420 |
| TBX3 | T-box 3 (ulnar mammary syndrome) | miR-363 | Inhibition | By using Target Scan algorithm was demonstrated that TBX3 is a target of mouse microRNA 363. | 18648548;21256124 |
|  |  | miR- 499 | Inhibition | Thrombospondin 4 is the target of microRNA 499 as revealed by RISC RNA sequencing. | 21030712 |
|  |  | miR-199a | Inhibition | Thrombospondin 1is a putative target of miR-199a*. | 22028325 |
|  |  | miR-18a | Inhibition | MicroRNA 18a physically interacts with Thrombospondin 1 and decreases its activity. | 16878133;18779589;20585343;21401928;22028325 |
|  |  | miR-1 | Inhibition | Thrombospondin 1is a putative target of miR-1. | 22028325;23024758 |
| THBS1 | thrombospondin 1 | miR-200a | Inhibition | MiR-200a can directly bind to THBS1 3'UTR and negatively regulate THBS1 expression. | 21698760;22931291 |
|  |  | miR-19b | Inhibition | Thrombospondin 1is a putative target of miR-19b. | 22028325;22768240 |
|  |  | miR-206 | Inhibition | Thrombospondin 1is a putative target of miR-206. | 22028325 |
|  |  | miR-19a | Inhibition | miR-19 targets the potent angiogenesis-inhibitor Tsp-1. | 16878133;19540203;20585343;22028325 |
|  |  | miR-let-7g | Inhibition | Thrombospondin 1is a putative target of miR-let-7g. | 22028325 |
|  |  | miR-194 | Inhibition | miR-194 is a direct regulator of THBS1 3'UTR. | 22028325 |
|  |  | miR-144 | Inhibition | Thrombospondin 1is a putative target of miR-144. | 22028325 |
|  |  | miR-448 | Inhibition | miR-448 targets the Klf5 3'-intranslated region. | 20719859 |
| KLF5 | Kruppel-like factor 5 (intestinal) | miR-145 | Inhibition | miR-145-mediated phenotypic modulation of VSMCs is through its target gene KLF5 and its downstream signaling molecule, myocardin. | 19542014;19720868;21945499;22389628;22931291 |
|  |  | miR-375 | Inhibition | miR-375 specifically targets KLF5 | 21278735 |
| QKI | quaking homolog, KH domain RNA binding (mouse) | miR-574 | Inhibition | miR-574-5p is a potent ribo-regulator for Qkis. | 22490519 |
|  |  | miR-214 | Inhibition | Quaking is a direct target of miR-214. | 22227154 |
| PROX1 | Prospero-related homeobox 1 | miR-181a | Inhibition | miR-181a binds the 3' untranslated region of Prox1, resulting in translational inhibition and transcript degradation. | 20558617;22442671 |
|  |  | miR-27a | Inhibition | miR-27a overexpression in osteoblasts and osteosarcoma cell lines results in dowregulation of TargetScan predicted MAPK14 target mRNA. | 22350417;23034448 |
|  |  | miR- 17 | Inhibition | It was found that Stat3 and Mapk14 are key direct targets of miR-17, miR-20a, and miR-106b and showed that simultaneous overexpression of Stat3 and Mapk14 mimics the alteration of E-Cadherin distribution observed after miR-17, miR-20a, and miR-106b downregulation. | 19559694 |
|  |  | miR- 106b | Inhibition | It was found that Stat3 and Mapk14 are key direct targets of miR-17, miR-20a, and miR-106b and showed that simultaneous overexpression of Stat3 and Mapk14 mimics the alteration of E-Cadherin distribution observed after miR-17, miR-20a, and miR-106b downregulation. | 19559694 |
|  |  | miR-125a | Inhibition | p38alpha is a direct target of microRna 125a. | 20145202 |
| MAPK14 | mitogen-activated protein kinase 14 | miR-200a | Inhibition | Overexpression of miR-141 or miR-200a severely reduced the total amount of p38alpha protein under either basal or stressed conditions. | 22101765 |
|  |  | miR-124 | Inhibition | MAPK14 is described as validated miR-124 target. | 20881268;22007158;22333974;22589088;22929886;22937097 |
|  |  | miR- 124a-2 | Inhibition | MicroRNA 124a-2 physically interacts with p38alpha (MAPK14) and decreases its activity. | 15806104;17254305;19536157;19901979 |
|  |  | miR- 124a-1 | Inhibition | MicroRNA 124a-1 physically interacts with p38alpha (MAPK14) and decreases its activity. | 15806104;17254305;19536157;19901979 |
|  |  | miR- 20a | Inhibition | It was found that Stat3 and Mapk14 are key direct targets of miR-17, miR-20a, and miR-106b and showed that simultaneous overexpression of Stat3 and Mapk14 mimics the alteration of E-Cadherin distribution observed after miR-17, miR-20a, and miR-106b downregulation. | 19559694 |
|  |  | miR- 124a-3 | Inhibition | MicroRNA 124a-3 physically interacts with p38alpha (MAPK14) and decreases its activity. | 15806104;17254305;19901979 |
|  |  | miR-141 | Inhibition | Overexpression of miR-141 or miR-200a severely reduced the total amount of p38alpha protein under either basal or stressed conditions. | 22101765 |
| EFNB2 | ephrin-B2 | miR-20b | Inhibition | EFNB2 is a target of miR20b. | 22438230 |
|  |  | miR-199a | Inhibition | Caveolin-1 is a putative target of miR-199a-5p. | 20300586;21363966 |
|  |  | miR-107 | Inhibition | Caveolin-1, a critical regulator of the insulin receptor is a direct target gene of miR-103/107. | 21654750 |
|  |  | miR-103 | Inhibition | Cav1 is a direct target of miR-103 in both mouse and human cells. | 21654750 |
| CAV1 | caveolin 1, caveolae protein, 22kDa | miR-133a | Inhibition | miR-133a directly binds to CAV1 mRNA. | 21109942 |
|  |  | miR-375 | Inhibition | Real-time PCR analysis confirmed the inhibition of CAV-1 gene expression by mir-375. | 19289822 |
|  |  | miR-203 | Inhibition | Caveolin-1 is a predicted target of miR-203. | 21814748;22421148;22552153 |
|  |  | miR- 34b | Inhibition | Caveolin-1 is described as a potential microRNA 34b target, and its inhibition by this microRNA was demonstrated in experiment. | 18519671 |
|  |  | miR- 133a-2 | Inhibition | miR-133a directly binds to CAV1 mRNA. | 21109942 |
|  |  | miR- 34c | Inhibition | Caveolin-1 is described as a potential microRNA 34c target, and its inhibition by this microRNA was demonstrated in experiment. | 18519671 |
|  |  | miR-27b | Inhibition | miR-27b and miR-224 target EDNRA 3'UTR. | 20827281 |
| EDNRA | endothelin receptor type A | miR-200c | Inhibition | EDNRA was predicted to be miR-200c target. | 22144583 |
|  |  | miR- 224 | Inhibition | miR-27b and miR-224 target EDNRA 3'UTR. | 20827281 |
|  |  | miR-218 | Inhibition | miR-218 targets Robo1 mRNA by binding to its 3'-UTR and suppresses Robo1 mRNA translation | 20300657;20947829;21385904;22007158;22931291 |
| ROBO1 | roundabout, axon guidance receptor, homolog 1 (Drosophila) | miR-208a | Inhibition | ROBO1 is validated miR-208a target in DM2 patients. | 22768114 |
|  |  | miR-381 | Inhibition | ROBO1 is validated miR-381 target in DM2 patients. | 22768114 |
| **TGF beta signalling** | |  |  |  |  |
|  |  | miR-9 | Inhibition | The 3' untranslated regions (3' UTRs) of TGFBI, TRIM2, SIRT1 and BTBD3 are repressed by miR-9 and -181c, either alone or in combination. | 21720722;22911744 |
|  |  | miR-744 | Inhibition | The TGF-beta1 137-nucleotide UTR is a direct target of miR-744. | 21991303;22350160 |
|  |  | miR-663a | Inhibition | MiR-663 targets TGFbeta1 transcripts. | 20637737 |
|  |  | miR-485 | Inhibition | TGF-beta 1 is a predicted target of miR-485-5p. | 22053178 |
|  |  | miR-29b | Inhibition | MiR-29b decreased the expression of TGFbeta1 at the promoter, transcript, and protein levels. | 21273536 |
| TGFB1 | transforming growth factor, beta 1 (Camurati-Engelmann disease) | miR-21 | Inhibition | TGF-beta 1 has been predicted to be the microRNA 21 target, using Miranda algorithm. Antisense-microRNA 21 resulted in a significant increase in Renilla activity from pRL-TK reporters bearing the microRNA21 MREs from the TGFB1, PDCD4, RASA1 and RASGRP1 genes. | 18823650;19264808;19534746;21192833 |
|  |  | miR-214 | Inhibition | TGF-beta-1 is a predicted target of microRNA 214 regulation. | 20332227 |
|  |  | miR-199a | Inhibition | TGF-beta-1 is a predicted target of microRNA199a regulation. | 20332227;21552493 |
|  |  | miR-194 | Inhibition | TGF-beta-1 is a predicted target of microRNA 194 regulation | 20332227 |
|  |  | miR-181c | Inhibition | The 3' untranslated regions (3' UTRs) of TGFBI, TRIM2, SIRT1 and BTBD3 are repressed by miR-9 and -181c, either alone or in combination. | 21720722 |
|  |  | miR-142 | Inhibition | TGF-beta-1 is a target of microRNA 142 regulation. | 20332227 |
|  |  | miR- 34a | Inhibition | TGF-beta-1 is a predicted target of microRNA 34a regulation. | 20332227 |
|  |  | miR- 187 | Inhibition | TGF-beta 1 is microRNA 187 target. | 20332227 |
|  |  | miR-335 | Inhibition | MAPK1 is a predicted target of miR-335. | 21490936;22382496 |
|  |  | miR-320 | Inhibition | ERK2 is described as a predicted microRNA 320 target selected from the top 7% of potential targets listed in the miRanda, TargetScan, or PicTar databases, which play positive roles in osteoblast differentiation. | 18784367;19070389 |
| MAPK1 | mitogen-activated protein kinase 1 | miR-224 | Inhibition | CDC42, CDH1, PAK2, BCL-2 and MAPK1 are important targets of miR-224 in HCC. | 22989374 |
|  |  | miR-181a | Inhibition | MAPK1 was predicted to be miR-181a target. | 22442671;22956783 |
|  |  | miR-143 | Inhibition | MAPK1 was predicted to be miR-143 target. | 22942087;23029228 |
|  |  | miR- 122a | Inhibition | MAPK1 is a direct target of microRNA 122. | 20842632 |
|  |  | miR-96 | Inhibition | (ACVR2B, CACNB4 and MYRIP) were predicted and validated as common miR-96/miR-96* targets. | 22038834 |
|  |  | miR-96 | Inhibition | (ACVR2B, CACNB4 and MYRIP) were predicted and validated as common miR-96/miR-96* targets. | 22038834 |
|  |  | miR-455 | Inhibition | ActRIIB is a direct target of miR455-3p. | 22143896 |
| ACVR2B | activin A receptor, type IIB | miR-215 | Inhibition | miR-215-5p binds to and inhibits ActRIIB . | 22431721 |
|  |  | miR-200c | Inhibition | miR-200c-3p binds to and inhibits ActRIIB . | 22431721 |
|  |  | miR-194 | Inhibition | ACVR2B mRNA is a predicted target of miR-194. | 21696393;22431721 |
|  |  | miR-192 | Inhibition | ActRIIB is a predicted target of miR-192. | 21840938;22431721 |
|  |  | miR-141 | Inhibition | miR-141-3p binds to and inhibits ActRIIB . | 22431721 |
